# Transcriptional Response of ATP-Binding Cassette (ABC) Transporters to Insecticide in the Brown Planthopper, *Nilaparvata lugens* (Stål)

**DOI:** 10.3390/insects11050280

**Published:** 2020-05-02

**Authors:** Zhao Li, Tingwei Cai, Yao Qin, Yunhua Zhang, Ruoheng Jin, Kaikai Mao, Xun Liao, Hu Wan, Jianhong Li

**Affiliations:** Hubei Insect Resources Utilization and Sustainable Pest Management Key Laboratory, College of Plant Science and Technology, Huazhong Agricultural University, Wuhan 430070, China; Lucky_edmund@163.com (Z.L.); caitingwei@webmail.hzau.edu.cn (T.C.); qinyao1246282025@163.com (Y.Q.); zhangyunh6688@126.com (Y.Z.); jinshirley@126.com (R.J.); maokk@webmail.hzau.edu.cn (K.M.); liaoxun2012@163.com (X.L.); huwan@mail.hzau.edu.cn (H.W.)

**Keywords:** ABC transporters, detoxification, *Nilaparvata lugens*, insecticides

## Abstract

The ATP-binding cassette (ABC) transporter superfamily is one of the largest groups of proteins and plays a non-negligible role in phase III of the detoxification process, which is highly involved in the response of insects to environmental stress (plant secondary metabolites and insecticides). In the present study, in *Nilaparvata lugens*, we identified 32 ABC transporters, which are grouped into eight subfamilies (ABCA–H) based on phylogenetic analysis. The temporal and spatial expression profiles suggested that the nymphal stages (1st–5th) and adult males showed similarity, which was different from eggs and adult females, and *NlABCA1*, *NlABCA2*, *NlABCB6*, *NlABCD2*, *NlABCG4*, *NlABCG12*, *NlABCG15*, and *NlABCH1* were highly expressed in the midgut and Malpighian tubules. In addition, *ABCG12*, which belongs to the ABC transporter G subfamily, was significantly upregulated after exposure to sulfoxaflor, nitenpyram, clothianidin, etofenprox, chlorpyrifos, and isoprocarb. Moreover, verapamil significantly increased the sensitivity of *N. lugens* to nitenpyram, clothianidin, etofenprox, chlorpyrifos, and isoprocarb. These results provide a basis for further research on ABC transporters involved in detoxification in *N. lugens*, and for a more comprehensive understanding of the response of *N. lugens* to environmental stress.

## 1. Introduction

ATP-binding cassette (ABC) transporters are transmembrane proteins widespread in various organisms, and are found in a variety of organelles, including the endoplasmic reticulum, Golgi, lysosomes, and plasma membranes [1,2]. Eukaryotic ABC transporter proteins include two forms: full transporters contain two transmembrane domains (TMDs) and two nucleotide binding domains (NBDs); half transporters contain one NBD and one TMD, and homo- or heterodimers are required to constitute a functional pump in half transporters [3]. Almost all NBDs contain several conserved motif sequences, such as the ABC signature, Walker A, Walker B, D-loop, Q-loop, and H-motif [4,5]. Based on the sequence similarity of the NBD domain, ABC transporters can be classified into eight subfamilies, A–H [6,7]. To date, ABC transporter genes have been identified in some insect species, including *Drosophila melanogaster*, *Plutella xylostella*, *Bombyx mori*, *Anopheles gambiae*, *Bemisia tabaci*, *Tribolium castaneum*, *Laodelphax striatellus, and Helicoverpa armigera* [8,9,10,11,12,13,14,15].

According to their function, ABC proteins can be divided into import proteins, export proteins and nontransport proteins [16]. In the ABC transporter subfamily, the ABCB, ABCC, and ABCG subfamilies are associated with multidrug resistance in both prokaryotes and eukaryotes [17]. In humans, modulation of *ABCB6* expression levels in human glioblastoma cells resulted in a concomitant change in cadmium sensitivity [18]. Furthermore, overexpression of the *ABCG1* gene can promote intracellular cholesterol to extracellular transport to accelerate extracellular lipoprotein synthesis [19]. Similarly, ABC transporters also play an important role in insect detoxification metabolism [20]. In *Bemisia tabaci*, ABC transporter subfamily G expression was significantly upregulated after exposure to imidacloprid [21]. Moreover, the *AsABCG28* gene of *Anopheles sinensis* is associated with pyrethroid detoxification [22]. When ABC transporters were inhibited via verapamil, the sensitivity of *H. armigera* to abamectin and indoxacarb was enhanced significantly, indicating that the ABC transporters were involved in the detoxification of *H. armigera* [15]. Recently, 13 ABCG subfamily genes were identified in *N. lugens*, and the results indicated that some ABCG transporter genes were able to respond to insecticide stress, such as thiamethoxam, abamectin, and cyantraniliprole [23].

The brown planthopper (BPH), *Nilaparvata lugens* (Stål), is one of the most notorious pests of rice crops and is widely distributed in Asian countries [24,25,26]. Due to heavy and frequent application of chemical insecticides, *N. lugens* has developed different levels of resistance to various insecticides [27,28,29]. Based on the latest data from APRD, *N. lugens* has developed resistance to 33 compounds with 419 reported cases, and *N. lugens* was ranked 11th of the top 20 most resistant species [30]. Resistance leads to an increase in the use of insecticides, which places considerable pressure on the environment [31,32]. The main cause of *N. lugens* resistance is the enhancement of detoxification and metabolism [33,34,35]. To date, many studies have reported on the roles of cytochrome P450s, carboxylesterases, glutathione *S*-transferases, and UDP-glycosyltransferase detoxification metabolism, but only a few reports have focused on ABC transporters.

In this study, we first identified and characterized the superfamily of ABC transporters from the transcriptome databases of *N. lugens*. Second, the spatiotemporal expression profile of ABC transporters was analyzed using qRT-PCR. In addition, the gene expression profiles of *NlABCs* after exposure to insecticides were also obtained. Finally, we determined the sensitivity of *N. lugens* to six insecticides before and after inhibitor treatment. These results provide a basis for further research characterizing the role of ABC transporters in the process of insecticide stress in *N. lugens*.

## 2. Materials and Methods

### 2.1. Insects and Insecticides

The population of *N. lugens* was initially collected in the Hunan Academy of Agricultural Sciences and has been maintained in the laboratory at Huazhong Agricultural University for more than 13 years without exposure to any insecticide. Insects were reared at 27 ± 1 °C and 70–80% relative humidity (RH) on rice seedlings with a photoperiod of 16:8 h light/dark. Sulfoxaflor (97.9%) was supplied by Dow AgroSciences Inc. (Indianapolis, IN, USA). Nitenpyram (95.8%), clothianidin (96%), chlorpyrifos (98%), etofenprox (95%), and isoprocarb (97.4%) were purchased from Hubei Kangbaotai Fine-Chemicals Co., Ltd. (Wuhan, China). Verapamil, a water-soluble inhibitor of ABC transporters [36,37], was purchased from Aladdin Biochemical Technology Co., Ltd. (Shanghai, China).

### 2.2. Collection of Tissues

Insects (*N. lugens*) at different developmental stages—eggs, first instar nymphs (N_1st), second instar nymphs (N_2nd), third instar nymphs (N_3rd), fourth instar nymphs (N_4th), fifth instar nymphs (N_5th), adult males and females were obtained from *N. lugens* reared on fresh rice seedlings. In addition, a total of 600 healthy 5th instar nymphs of *N. lugens* individuals were dissected on ice under a stereomicroscope (Zeiss, Jena, Germany) for head, epidermis, fat body, midgut, and Malpighian tubule collection. The fat body, midgut, and epidermis were collected and washed with phosphate-buffered saline (PBS; 140 mM NaCl, 27 mM KCl, 8 mM Na_2_HPO_4_, and 1.5 mM KH_2_PO_4_, pH 7.4). The collected tissue samples were stored at −80 °C for further RNA extraction.

### 2.3. RNA Isolation, cDNA Synthesis

Total RNA was extracted from three pools of tissues, different stages, and survived induction nymphs using RNAiso Plus (Takara, Shiga, Japan), following the manufacturer’s instructions. DNA contamination was removed by treating RNA extraction products with gDNA digester (Yeasen, Shanghai, China). The quality and concentration of RNA samples were checked spectrophotometrically at 260/280 nm using an NP80 UV–Vis spectrophotometer (IMPLEN, Munich, Germany). One microgram of total RNA was reverse transcribed to first-strand cDNA by using Hifair™ II 1st Stand cDNA Synthesis SuperMix (Yeasen, Shanghai, China).

### 2.4. Gene Identification and Phylogenetic Analysis

To identify open reading frames (ORFs) encoding putative ABC transporters, we used the highly conserved NBDs (as defined by PF00005) and 32 ABC genes in *N. lugens* as queries to search against the updated GLEAN gene in the *N. lugens* genome (Accession NO, PRJNA177647) by local BLASTP, with an E-value threshold of 10^−5^. Each potential ABC transporter was further analyzed by the programs Pfam and SMART to identify any NBD and TMD domains. The “Compute pI/Mw” (http://au.expasy.org/tools/pi_tool.html) in SWISS-PROT (ExPASy Server) was used to calculate molecular weight and theoretical isoelectric points [38]. The protein sequence alignment for NBDs of *N. lugens* and *Laodelphax striatellus* (*L. striatellus*) were aligned using ClustalW, and then subjected to phylogenetic analysis using the maximum likelihood method with 1000 replicates by MEGA7 [39].

### 2.5. qRT-PCR Analysis

Quantitative real-time polymerase chain reaction (qRT-PCR) was performed in a MyIQ2 real-time PCR detection system (Bio-Rad, Hercules, CA, USA) using UNICON™ qPCR SYBR Green Master Mix (Yeasen, Shanghai, China), following the manufacturer’s protocol. Briefly, the qPCR reaction mixture (10 μL) included 5 μL SYBR Premix Ex Taq, 3.5 μL ddH_2_O, 0.5 μL each of forward and reverse gene-specific primers, which are listed in Table S1, and 0.5 μL cDNA template. The qRT-PCR reaction was as follows: an initial incubation at 50 °C for 2 min and then 95 °C for 2 min, followed by 40 cycles of 95 °C for 15 s and 60 °C for 30 s. The guanine-N (7)-methyltransferase gene (*Nl18s*) expression remained constant among all of the strains; therefore, the mRNA levels of the ABC transporter genes in tissues, different instars, and survived induction 3th instar nymphs were normalized to the level of expression of *Nl18s*. The 2^−^^ΔΔCT^ method was used to obtain the relative quantifications [40]. Three independent biological replicates that included three technical replications were carried out for each test.

### 2.6. Bioassay

The LC_50_ for each insecticide (inducted concentration) was carried out using third instar nymphs, according to a previously reported rice dipping method [28]. This concentration was used to induce insecticide stress in 200 3rd instar nymphs. Fifteen rice seedlings were grouped together, dipped into a concentration of insecticide dilutions for 30 s, and then air-dried at room temperature for more than 30 min. Cotton moistened with water was used to wrap the roots of rice seedlings, and they were then placed in a 500 mL plastic cup. Fifteen third-instar nymphs were collected for each replicate. Three replicates of each concentration and six replicates of each insecticide were carried out, with rice seedlings dipped in water containing 0.1% Triton X-100 as control. The surviving nymphs were selected after 48 h for RNA isolation.

The solutions of verapamil were determined using third instar nymphs, according to a previously reported rice dipping method [28]. The solutions of verapamil (200 mg/L, approximately LC_15_, shown in Table S2) were diluted to the required concentrations with 0.1% Triton X-100 solution. The rice-seedling dip method was used for the bioassay described previously [32]. The insecticides were diluted to generate seven or eight serial dilutions (sulfoxaflor, 0–6.4 mg/L; nitenpyram, 0–3.2 mg/L; clothianidin, 0–12.8 mg/L; etofenprox, 0–640 mg/L; chlorpyrifos, 0–120 mg/L; isoprocarb, 0–240 mg/L) with or without verapamil solution, as described previously. Rice seedlings dipped in water containing 0.1% Triton X-100 with or without verapamil solution served as controls. Treated insects in plastic cups were kept at 27 ± 1 °C and 70%–80% RH with a 16:8 h light/dark photoperiod. Mortality was scored after exposure to isoprocarb, ethofenprox, and chlorpyrifos for 72 h, and to sulfoxaflor, nitenpyram, and clothianidin for 96 h.

### 2.7. Data Analysis

Abbott’s formula was used to control mortality for mortality data obtained. The slopes with standard errors, and LC_50_ values with 95% confidence intervals, were calculated by DPS software 7.05 (ZheJiang University, HangZhou, China). Expression levels of NlABC genes in different tissues and stages were normalized by the expression of the lowest gene and converted into logarithmic data. Expression levels of NlABC genes in response to different insecticide treatments were normalized by the expression of the respective gene in untreated nymphs. Then, the data were subjected to a one-way ANOVA, followed by Fisher’s least significant difference (LSD) multiple comparison test using the SPSS 22.0 software (SPSS Inc., Chicago, IL, USA) with more than three repeats. Difference significance was checked at *p* < 0.05.

## 3. Results

### 3.1. Identification of ABC Transporters in N. Lugens

**Figure 1:**
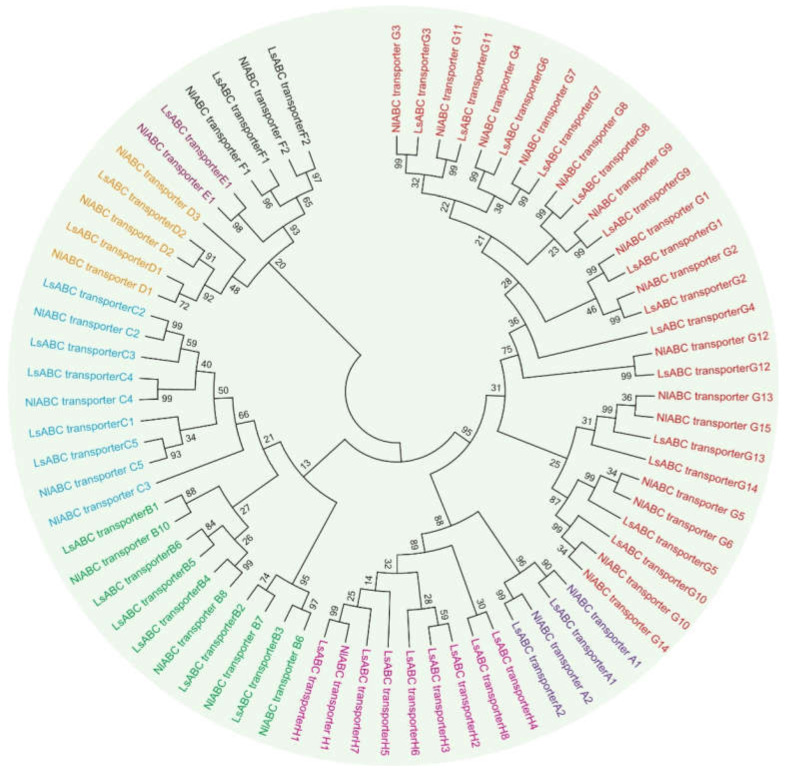
Phylogenetic tree of the *Nilaparvata lugens* ATP-binding cassette (ABC) transporters. Amino acid sequences of nucleotide-binding domains (NBDs) from *N. lugens* (Nl) and *Laodelphax striatellus* (Ls) were aligned using ClustalW and analyzed by the maximum likelihood method in MEGA7 [39]. The bootstrap consensus tree, inferred from 1000 replicates, is taken to represent the evolutionary history of the taxa analyzed. The bootstrap values are shown on the branches. Each color represents one ABC subfamily: A–H.

To identify the ABC transporter genes in *N. lugens*, we searched the full-length transcriptome of *N. lugens*, and 32 unique ABC transporter segments were identified according to the specific subfamilies to distinguish their belongings. Based on the phylogenetic analysis of NBDS, the ABC transporter gene superfamily in *N. lugens* was grouped into eight subfamilies, named A to H (according to the homology of *L. striatellus*) (Figure 1). Each ABC transporter gene had one or two conserved NBDs, which have several characteristic motifs, including the Walker A and B motifs common to many nucleotide binding proteins and others, such as the ABC signature, stacking aromatic D, H, and Q loops, which are unique to the family (Table S3). Briefly, of the 32 ABC transporters, 6 are full transporters, 19 are half transporters, 3 have two NBDs, and 4 have one NBD but lack TMD segments. Subfamily G had the most genes with fifteen, followed by subfamily C and subfamily B with four. Three genes belonged to subfamily D. Subfamily A and subfamily F had two genes. One belonged to subfamily E, and one belonged to subfamily H. The name, accession number, length, position based on the AA sequence, topology, molecular weight, and theoretical pI are listed in Table 1.

### 3.2. Expression Profiles of ABC Transporters in N. Lugens

**Figure 2:**
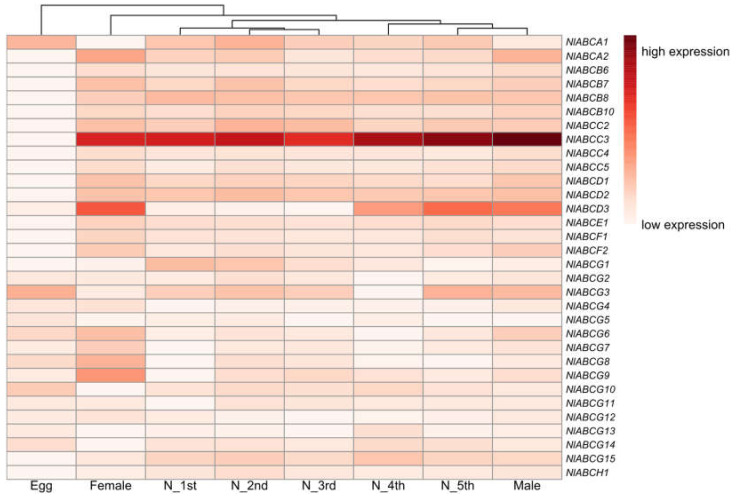
Relative expression levels of ABC transporters at different developmental stages in the *N. lugens* life cycle, as determined by qRT-PCR. Letters on the right are the gene names for the 32 ABC transporters. Developmental stages are given at the bottom. The mRNA levels, represented by normalized log10 (∆CT) values, are shown in the gradient heatmap. The color from light to dark indicates the expression level from low to high.

**Figure 3:**
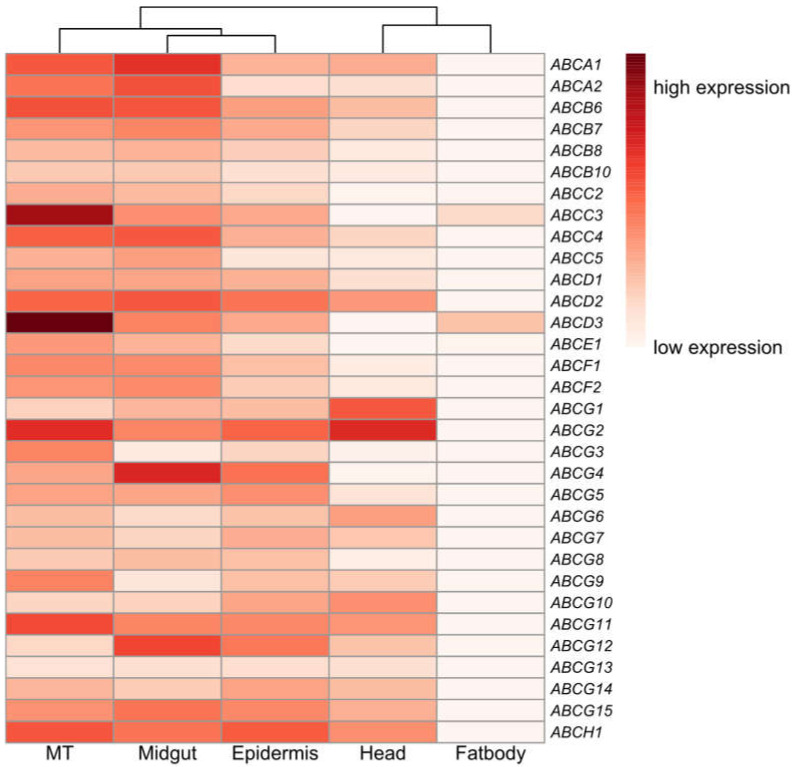
Relative expression levels of ABC transporters in different tissues of 5th instar *N. lugens*, as determined by qRT-PCR. Letters on the right are the gene names for the 32 ABC transporters. Tissue types are given at the bottom; MT, Malpighian tubules. The mRNA levels, represented by normalized log10 (∆CT) values, are shown in the gradient heatmap. The color from light to dark indicates the expression level from low to high.

To determine the expression profiles of ABC transporter genes in *N. lugens*, RNA from different developmental stages (eggs, nymphs, adults) and different tissues (head, epidermis, fat body, midgut, Malpighian tubules) were extracted. As shown in Figure 2 and Figure 3, the expression patterns of each gene differed among developmental stages. Most genes showed developmental-specific expression. The egg stage is clustered with nymph stages and adult males. Female adults are clustered with all other samples. The expression levels of *NlABCA1*, *NlABCG3*, and *NlABCG10* in the egg period were higher than those in the other stages. *NlABCA1*, *NlABCA2*, *NlABCB8*, *NlABCC3*, *NlABCD2*, *NlABCG1*, *NlABCG3*, and *NlABCG15* were highly expressed in the first instar and second instar, and two ABCC subfamily genes (*NlABCC2* and *NlABCC3*) were highly expressed in the third instar nymphs. *NlABCE1* was highly expressed at the 4th and 5th instar nymphs and at the male and female stages. Moreover, a large number of ABC genes were highly expressed in the midgut and included members in subfamily A (*NlABCA1*, *NlABCA2*), subfamily B (*NlABCB6*), subfamily D (*NlABCD2*), subfamily G (*NlABCG4*, *NlABCG12*, *NlABCG15*), and subfamily H (*NlABCH1*). Numbers of subfamily A (*NlABCA1*, *NlABCA2*), subfamily B (*NlABCB6*), subfamily C (*NlABCC3*, *NlABCC4*), subfamily D (*NlABCD2*, *NlABCD3*), subfamily G (*NlABCG2*, *NlABCG11*), and subfamily H (*NlABCH1*) were highly expressed in the Malpighian tubules. In epiderma, only *NlABCD2*, *NlABCG12*, and *NlABCH1* showed high expression performance. In contrast, almost all ABC transporter genes show low expression patterns in fat bodies. These results imply that perhaps due to different functions, the expression patterns of ABC transporters in different stages and in different tissues are distinguished.

### 3.3. Influence of Six Insecticides on ABC Transporter Expression

**Figure 4:**
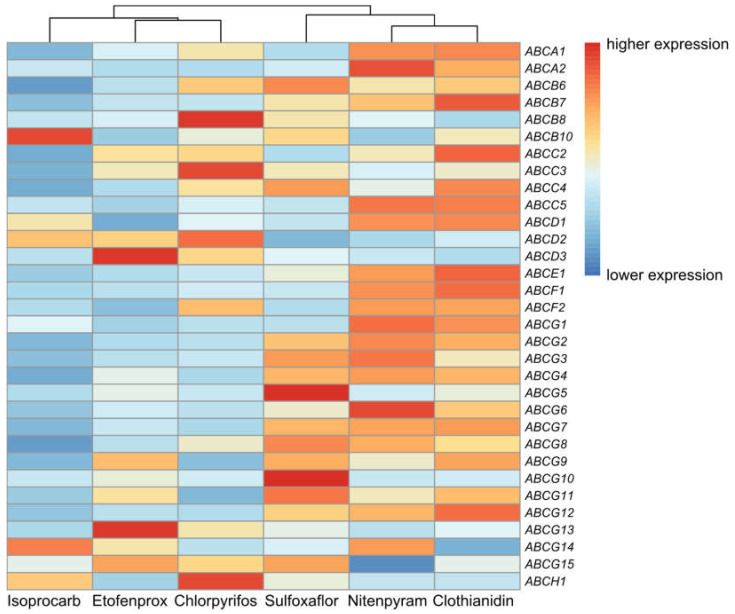
Relative expression levels of 32 ABC transporters in *N. lugens* nymphs treated with insecticides, as determined by qRT-PCR. Letters on the right are the gene names for the 32 ABC transporters. Treatments are given at the bottom. The mRNA levels were normalized by the expression of the respective gene in untreated nymphs. Blue indicates downregulated, and red indicates upregulated. The more intense the color, the more pronounced are the changes in expression levels.

**Figure 5:**
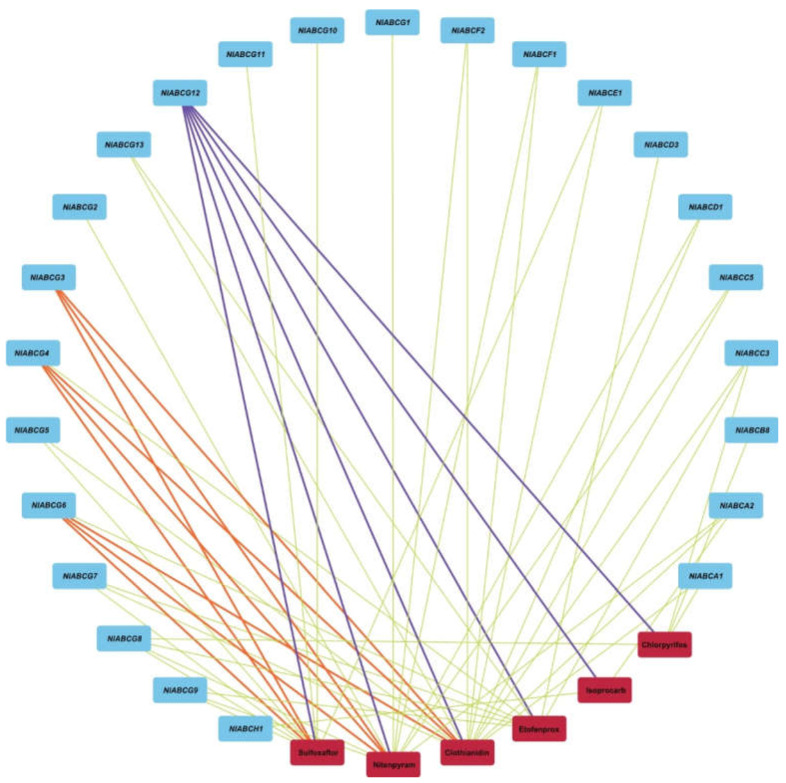
Summary of genes that were significantly upregulated after exposure to different insecticides. The link indicates that the gene was significantly upregulated after insecticide induction. The orange line indicates the genes that are upregulated after induction by three neonicotinoid insecticides. The purple line indicates genes that are upregulated after induction of six insecticides (*p* < 0.05).

To investigate whether the expression of ABC transporter genes was affected by insecticides, we determined the response after insecticide treatment (Figure 4). In response to sulfoxaflor, ten genes belonging to subfamily G (*NlABCG3/G4/G5/G6/G7/G8/G9/G10/G11/G12*) and one gene from subfamily E (*NlABCE1*) were significantly overexpressed. One gene from subfamily G (*NlABCG14*) showed downgraded expression. Subfamily A (*NlABCA2*), subfamily C (*NlABCC5*), subfamily D (*NlABCD1*), subfamily F (*NlABCF1/F2*), and subfamily G (*NlABCG1/G2/G3/G4/G6/G8/G9/G12*) were significantly overexpressed after treatment with nitenpyram. In contrast, two genes, *NlABCD3* and *NlABCG15,* showed significantly downregulated expression. After exposure to clothianidin, subfamily A (*NlABCA1/A2*), subfamily C (*NlABCC3/C5*), subfamily D (*NlABCD1*), subfamily E (*NlABCE1*), subfamily F (*NlABCF1/F2*), and subfamily G (*NlABCG3/G4/G5/G6/G7/G12/G13*) were significantly overexpressed, and subfamily D (*NlABCD3*), subfamily G (*NlABCG14/G15*), and subfamily H (*NlABCH1*) were significantly downregulated. Facing the stress of the pyrethroid insecticide etofenprox, nine genes, *NlABCC3* from subfamily C, *NlABCD3* from subfamily D, and *NlABCG4/G6/G7/G8/G9/G12/G13* from subfamily G, were significantly overexpressed, and none showed significant reduction. Organophosphorus insecticide chlorpyrifos induction caused upregulated expression of genes, such as *NlABCA1*, *NlABCB8*, *NlABCC3*, and *NlABCG12*. When treated with carbamate insecticide isoprocarb, the expression of ABC genes *NlABCA2*, *NlABCG12,* and *NlABCH1* from subfamily A, subfamily G, and subfamily H were significantly upregulated. In contrast, *NlABCC2*, *NlABCD3*, and *NlABCG6* were downregulated. Overall, after exposure to three neonicotinoid insecticides, four ABC transporters from subfamily G, *NlABCG3*, *NlABCG4*, *NlABCG6*, and *NlABCG12*, were all significantly upregulated. After exposure to six insecticides, which belonged to neonicotinoid, pyrethroid, organophosphorus, and carbamate, *NlABCG12* from subfamily G was significantly upregulated compared with the control (Figure 5). None of the ABC transporter genes simultaneously showed downregulated expression after insecticide induction.

### 3.4. Synergistic Effects of Verapamil on Insecticides

The putative inhibitor verapamil was used to verify the effects of ABC transporters in *N. lugens* defense against insecticides. The results show that the sensitivity of *N. lugens* to insecticides changed when verapamil was used in combination with insecticides (Table 2). Susceptibility to nitenpyram, clothianidin, etofenprox, chlorpyrifos, and isoprocarb was significantly increased in the verapamil treatment group, compared to the control (based on nonoverlapping fiducial limits), and the synergism ratio was 4.67, 1.92, 2.79, 1.57, and 2.01, respectively. The bioassay of sulfoxaflor showed no significant changes in the verapamil treatment group, compared with the control group. These results suggest that ABC transporters were involved in the detoxification of insecticides in *N. lugens*.

## 4. Discussion

In the present study, we estimate that there were 32 putative ABC transporter genes in the monophagous hemiptera species *N. lugens*, conservatively. The results showed that the 32 ABC transporters we identified were subordinate to the A–H subfamily through phylogenetic analysis, similar to, but slightly fewer than those species reported previously, such as *Laodelphax striatellus* (40 ABC transporters), *Lygus hesperus* (65), *Drosophila melanogaster* (56), and *Anopheles gambiae* (52), *Tribolium castaneum* (73), *Apis mellifera* (43), *Bombyx mori* (55), *Daphnia pulex* (65), *Tetranychus urticae* (103), *Homo sapiens* (48), *Helicoverpa armigera* (54), and *Plutella xylostella* (82).

In the phylogenetic tree, a clade of two ABCA genes in *N. lugens* was identified, which includes two TMDs and two NBDs called full transporters, similar to the ABCA subfamily gene of *T. castaneum*, which participates in the wing development process [10]. In the ABCB subfamily of *N. lugens*, four half-transporters were identified. Some members of the ABCB subfamily of insects are thought to be involved in the metabolism of insecticides and play an integral role in the formation of resistance. The *D. melanogaster* ABCB subfamily participates in the formation of cadmium tolerance [41]. We also identified four full-transporter ABCC subfamily genes. The ABCC subfamily may play a role in a variety of physiological activities, such as enhancing the tolerance of *Pediculus humanus* to ivermectin [42,43]. Interestingly, almost all insect species have two ABCD genes (half-transporters), but in our results, we identified three ABCD genes. ABCD subfamily members are involved in the transport of long-chain branched acyl-CoA molecules to peroxisomes in humans [44]. One and two genes were identified from the ABCE subfamily and ABCF subfamily in *N. lugens*, respectively. Members of the ABCE and ABCF subfamily contain only two NBDs and lack TMDs, so ABCE and F proteins do not have transport functions. However, the human *ABCE1* protein plays an important role in nuclear protein biosynthesis and transcriptional regulation [45,46]. Injection of dsRNA of *T. castaneum ABCE-3A* and *ABCF-2A* can increase the mortality rate of nymphs [10]. We identified 15 genes from the largest ABC subfamily. Almost all ABCG transporters contain one NBD domain and one TMD domain. The ABCG subfamily has been confirmed to participate in multiple life activities in living organisms, such as synthetic pigment precursors, courtship behavior, uptake of uric acid, and transport of biogenic amines, lipids [47,48,49,50]. In addition to these functions, insecticide caused changes in the gene expression levels of the ABC transporter G family [51]. Recently, the ABCG subfamily was also reported in *N. lugens*. Research shows that the ABCG transporter is able to respond to insecticides, such as thiamethoxam, abamectin, and cyantraniliprole [23]. We identified only one ABCH gene in *N. lugens*, and this family gene function has rarely been reported. Injection of *dsTcABCH-9C* in *T. castaneum* will show lethal effects [10]. RNA interference (RNAi) demonstrated that *LmABCH-9C* is needed for desiccation resistance in *Locusta migratoria* [52]. Taken together, our findings hypothesized that we had found most of the ABC transporters in *N. lugens*, which belonged to ABCA–ABCH. The primary structure of ABC transporter genes suggests that ABC transporters in *N. lugens* are structurally and functionally similar to other insects.

We found that the complex expression of the ABC gene at different developmental stages and in different tissues may be due to differences in nutrient requirements and physiological behavior at different developmental stages, as well as the specific functions of different tissues. Different functions lead to different substances that need to be transferred. Our study found that the expression pattern of ABC transporters in all nymphs’ stages and male adults were more similar than that of eggs and female adults. This result is consistent with the theory of insect metamorphosis [10]. Our results also found that ABC transporter genes were highly expressed in the midgut and Malpighian tubules. The midgut and Malpighian tubules are considered to play an important role in physiological process. For instance, the midgut-specific ABCG gene, BmABC005226, was regulated by 20E at the transcriptional level [9]. In desert locusts, ABC transporters in Malpighian tubules could remove xenobiotic substances from the hemolymph [53].

It has been reported that ABC transporters are involved in transport or resistance of various insecticides [54,55,56,57]. We found that most of the ABC transporter genes changed their expression after exposure to insecticides in *N. lugens*. In our study, chlorpyrifos upregulated a number of ABC genes, such as *NlABCA1*, *NlABCB8*, *NlABCC3*, *NlABCG8*, and *NlABCG12*. Similar to our results, ABC genes from subfamilies A, B, C, D, and G had upregulated expression after feeding on chlorpyrifos-treated *H. armigera* [15]. Furthermore, our study showed that 15 ABC transporter genes were upregulated after clothianidin treatment. Similarly, 13 ABC genes were upregulated in the deltamethrin-resistant strains in *L. striatellus* [13]. These results indicate that ABC transporters are not strictly targeted to different insecticides. In addition, we found that in the face of neonicotinoid insecticide stress, such as sulfoxaflor, nitenpyram, and clothianidin, up to a total of 19 ABC transporter genes were significantly upregulated. Similar research found that 11 ABC genes were upregulated in imidacloprid-resistant strains [13]. From our results, we also found that *NlABCG3/G4/G6* and *NlABCG12* were all upregulated after treatment with sulfoxaflor, nitenpyram, and clothianidin. This may be because they belong to the same class of insecticides and have a common chemical structure. Similarly, ABCG4 gene was upregulated after treatment with permethrin in *Anopheles gambiae s.s.* and *Anopheles stephensi* [58,59]. Verapamil combined with bioassays demonstrates the contribution of ABC transporters to the resistance mechanisms of several insects [36,37,60,61]. We determined the LC_50_ of the six insecticides in the presence of the inhibitor verapamil. Our results showed that the LC_50_ values of nitenpyram, clothianidin, chlorpyrifos, chlorpyrifos, and isoprocarb were all significantly decreased. Interestingly, many ABC transporter genes showed upregulation under sulfoxaflor stress, but the sensibility to sulfoxaflor with inhibitor was increased slightly. It suggested that ABC transporters were activated in response to sulfoxaflor stress, and led to a partial reduction in toxicity to sulfoxaflor in *N. lugens.* Combined with our previous experimental results, insecticide induction upregulated the expression of the ABC transporter in brown planthoppers, and we concluded that the ABC transporter is not only involved in responding to insecticides in *N. lugens*, but also plays a non-negligible role.

## 5. Conclusions

In this study, 32 ABC transporter genes were identified in *N. lugens*. The spatiotemporal expression profile reveals the expression pattern of ABC transporters in *N. lugens*. In addition, this study provides evidence of a functional role for the ABC transporter during the insecticide stress response of *N. lugens*. Our results provide a theoretical foundation for future studies on the detoxification of insecticides by ABC transporters in *N. lugens*.

## Figures and Tables

**Table 1:** Details on the 32 ABC transporters identified in *Nilaparvata lugens*.

Gene Name	Accession Number	Length (aa)	Positions Based on the AA Sequence		Topology (SMART)	Molecular Weight ^b^	Theoretical pI ^b^
			Predicted TM ^a^	Predicted ABC Domain ^a^			
*NlABCA1*	FLNC_seq_9841	1636	1:77–375	1:507–654	TMD-NBD-TMD-NBD	184385.85	7.20
			2:865–1260	2:1333–1476			
*NlABCA2*	FLNC_seq_144369	2263	1:506–868	1:943–1096	TMD-NBD-TMD-NBD	254493.05	7.64
			2:1257–1818	2:1891–2033			
*NlABCB6*	FLNC_seq_724	834	1:251–529	592–741	TMD-NBD	94308.65	7.26
*NlABCB7*	FLNC_seq_104535	755	1:166–451	1:516–664	TMD-NBD	83592.21	9.27
*NlABCB8*	FLNC_seq_75020	685	1:114–391	1:457–607	TMD-NBD	75115.2	8.95
*NlABCB10*	FLNC_seq_17028	725	1:147–414	1:482–635	TMD-NBD	79890.01	9.48
*NlABCC2*	FLNC_seq_37996	1397	1:97–380	1:488–623	TMD-NBD-TMD-NBD	156380.03	7.32
			2:798–1074	2:1139–1286			
*NlABCC3*	FLNC_seq_40930	1486	1:139–405	1:467–601	TMD-NBD-TMD-NBD	168557.59	5.98
			2:774–1018	2:1107–1254			
*NlABCC4*	FLNC_seq_170922	1437	1:197–460	1:561–695	TMD-NBD-TMD-NBD	159725.86	8.48
			2:857–1137	2:1205–1353			
*NlABCC5*	FLNC_seq_108959	1489	1:301–567	1:633767	TMD-NBD-TMD-NBD	165683.07	7.92
			2:941–1186	2:1269–1415			
*NlABCD1*	FLNC_seq_143415	675	1:130–403	1:530–663	TMD-NBD	74724.64	9.44
*NlABCD2*	FLNC_seq_49967	676	1:73–341	1:473–615	TMD-NBD	54941.38	9.64
*NlABCD3*	FLNC_seq_70646	300		1:70–214	NBD	33315.71	9.27
*NlABCE1*	FLNC_seq_32474	609		1:110–252	NBD-NBD	68510.06	7.79
				2:376–499			
*NlABCF1*	FLNC_seq_17408	588		1:54–213	NBD-NBD	65907.28	6.47
				377–506			
*NlABCF2*	FLNC_seq_42497	620		1:95–253	NBD-NBD	70313.61	7.01
				2:409–541			
*NlABCG1*	FLNC_seq_72048	408	1:331–541	1:42–186	NBD-TMD	71025.02	8.66
*NlABCG2*	FLNC_seq_16091	632	1:377–587	1:86–231	NBD-TMD	75896.09	9.20
*NlABCG3*	FLNC_seq_109711	681	1:403–610	1:85–231	NBD-TMD	75775.11	7.80
*NlABCG4*	FLNC_seq_10454	677	1:343–551	1:48–193	NBD-TMD	68808.99	8.83
*NlABCG5*	FLNC_seq_116166	971	1:649–866	1:363–505	NBD-TMD	106439.26	9.35
*NlABCG6*	FLNC_seq_10454	614	1:343–551	1:48–193	NBD-TMD	68808.99	8.83
*NlABCG7*	FLNC_seq_133740	712	1:439–642	1:68–214	NBD-TMD	79592.84	7.14
*NlABCG8*	FLNC_seq_65464	631	1:352–559	1:53–200	NBD-TMD	71071.5	8.52
*NlABCG9*	FLNC_seq_30333	608	1:336–544	1:26–172	NBD-TMD	68417.12	9.10
*NlABCG10*	FLNC_seq_35578	643		1:38–184	NBD	71030.7	9.12
*NlABCG11*	FLNC_seq_107711	326		1:41–191	NBD	35396.02	8.62
*NlABCG12*	FLNC_seq_148301	622	1:338–545	1:33–182	NBD-TMD	70006.1	8.70
*NlABCG13*	FLNC_seq_4034	723	1:446–653	1:71–220	NBD-TMD	82248.2	7.86
*NlABCG14*	FLNC_seq_35578	643		1:38–184	NBD	71030.7	9.12
*NlABCG15*	FLNC_seq_32119	760	1:483–690	1:108–257	NBD-TMD	85694.68	6.57
*NlABCH1*	FLNC_seq_44684	702	1:314–692	1:42–174	NBD-TMD	78743.34	5.53

^a^ TMs and ABC domains were predicted using the Pfam (http://pfam.xfam.org/search/sequence; EMBL-EBI, Hinxton, Cambridgeshire, UK); ^b^ Molecular Weight and Theoretical pI using the “Compute pI/Mw” (http://au.expasy.org/tools/pi_tool.html) in SWISS-PROT (ExPASy Server).

**Table 2:** Susceptibility of *N. lugens* nymphs to insecticides after treatment with inhibitor.

Insecticide	Treatment	Slope (± SE ^a^)	LC_50_ (mg/L)	95% CI ^b^	Synergism Ratio ^c^
Sulfoxaflor	DMF	2.35 ± 0.27	4.27	3.53~5.21	1.18
	Verapamil	2.07 ± 0.27	3.62	2.87~4.94	
Nitenpyram	DMF	1.25 ± 0.22	1.26	0.91~1.96	4.67
	Verapamil	1.24 ± 0.22	0.27	0.17~0.37	
Clothianidin	DMF	1.51 ± 0.23	5.65	4.30~7.91	1.92
	Verapamil	1.89 ± 0.24	2.95	2.26~3.69	
Etofenprox	DMF	2.61 ± 0.26	208.96	171.23~248.92	2.79
	Verapamil	2.45 ± 0.33	74.85	74.87~104.35	
Chlorpyrifos	DMF	3.18 ± 0.37	65.66	55.17~82.39	1.57
	Verapamil	2.69 ± 0.29	41.94	35.23~50.18	
Isoprocorb	DMF	2.42 ± 0.29	99.48	81.57~121.25	2.01
	Verapamil	2.13 ± 0.28	49.38	35.16~62.94	

^a^ Data are presented as the mean (standard error). ^b^ CI, confidence interval. ^c^ Synergism ratio = LC_50_ of insecticide/LC_50_ of insecticide + inhibitor.

## Supplementary Materials

The following are available online at https://www.mdpi.com/2075-4450/11/5/280/s1, Table S1: Primers for the ABC transporter genes and 18S gene for RT-qPCR, Table S2: Susceptibility of *N. lugens* nymphs to verapamil inhibitor, Table S3: NBD motif analysis of *N. lugens*.
