# Supplementary material for: Transcriptional Response of ATP-Binding Cassette (ABC) Transporters to Insecticide in the Brown Planthopper, Nilaparvata lugens (Stål)
Source: Insects. 2020 May 2;11(5):280. doi: 10.3390/insects11050280 (PMC7291042; doi:10.3390/insects11050280)
Supplement: Supplementary file 1 [file insects-11-00280-s001.pdf]

Table 1. Primers for the ABC transporter genes and 18S gene for RT-qPCR.

Gene Name	Forward (5′–3′)	Reverse (5′–3′)
NlABCA1	ATAATGCCCGGCGAGTATGG	ATTACGCACTGACAAGCCCA
NlABCA2	GGAATGTGCCTAGTCGGCAA	CGTCGGCTCGTCAAGGAATA
NlABCB6	GGAGCGGTCGAGTTCAGAAA	GTTCTGCCCATCCACCAGAA
NlABCB7	CAAGGGCAGGACGTCGATTA	GTTGTACAGTGAGTCGGGCA
NlABCB8	AGGAGAGAACTGGCGAGCTA	TATAGCGACACCACACAGCC
NlABCB10	GCGGGAGAAATGGAACCTCA	CAAAATTGCACAGTCCGCCA
NlABCC2	TGCATATTCGCGCACTACCT	AACGGGATGGCAACCAGTAG
NlABCC3	CAATCTACATGGGCGCGTTG	CCATCGTATACACGTCGCCA
NlABCC4	AGGGCTTGCCATTGCCTATT	GGGGCTTCGAAGTAACACCA
NlABCC5	TGCACGCGTACAGCAGGTTT	GACACGGGCAGATCGTAGAG
NlABCD1	GGCTTTGTCATTCAGGTCGC	GTCGAAGAGTGGCTTGGTGA
NlABCD2	GTTGCTGTTTCGAACGGCAT	TTCGGTGACTCTTGCCGTGA
NlABCD3	ATTGGGGTAGTCGGCTCAAG	GCTCTCAAACTGCCAGAAAGG
NlABCE1	GCCTCGCAGTAAAGCAACAG	TACGCACAACAGGACAGGAC
NlABCF1	AACTCTCGTGTCGCCATTGT	CATCAGGTATTCGGACGGGG
NlABCF2	CGCCAGGCTCAAAGTAAGGA	CTGGACCATGATGACCGGAG
NlABCG1	ACCAGCCGTTTCAGTTCCTTTG	GCCGCCGTAGATGGACGATATG
NlABCG2	TGTCCAGGAGAGGAGCCGTTAG	CATTCTGGTGAGCGTGGTCAGT
NlABCG3	AGACAGGCATCGTTGCACCAGC	CGGCTCGTGAGTTGTCAGGTAC
NlABCG6	GGATGAGCCAACTACAGGACTT	AAGGAAGCACTCGGTTGATGAA
NlABCG5	AGACGGCAAGAACAACGAACCT	TGTCCAGAATGGCACGACCTTC
NlABCG6	GAGTACTCCCCGGTCACTCT	GCGTCCAGTTGAAGGGTACA
NlABCG7	GGCTCGGTGAAGGTGAACATCC	TCGGAGACGGAGTAGGTGAGGT
NlABCG8	TGCTGCCGTTCCTGTCCTCAT	TGGACTCTTGGCGACCATGCT
NlABCG9	ACGAGTGGCTTGGACAATGTGT	GGCTGGCACTTGGCTGATGTAT
NlABCG10	GAGTACTCCCCGGTCACTCT	GCGTCCAGTTGAAGGGTACA
NlABCG11	CTCAAGCACGGTTCAACTGGTT	TCGTCCGCCATTGATGAGGTAA
NlABCG12	GGCTGGTGCTCTGCTCAATGTC	GCTGCCGCAACTAAGCGTCTT
NlABCG13	GGAGAGCTGTTGGCTGTCAT	GCGCTTGTTAAGGCGTTCTT
NlABCG14	GGCGAAGTGTGGCAAGTCTCA	GGCTCGTCCAATAGCAGCAGAA
NlABCG15	TCACTTGCCTCACAGACCAC	ATGACAGCCAACAGCTCTCC
NlABCH1	CGCAAGGAGCTCGGATACAT	GAAGCCCTCTGTTGGCAGAT

Table 2. Susceptibility of *N. lugens* nymphs to verapamil inhibitor.

Chemical	N	Slope (SE)	LC_{15} (mg/L)	LC_{50} (mg/L)	LC_{95} (mg/L)
verapamil	315	2.62 (0.29)	203.93	507.82	2160.49

Table 3. NBD motif analysis of *N. lugens*.

Gene Name	Walker A	Q-Loop	ABC Signature	Walker B	D-Loop	H-Loop
NlABCA1	GHNGAGKT	PQH	LSGGMKRKLCLA	ILILDEP	SGLD	DHG
NlABCA2	GHNGAGKT	PQH	LSGGMQRKLSVA	TVILDEP	SGVD	AHG
NlABCB6	GPSGSGKST	PQD	LSGGEKQRVAIART	VLLDEA	SALD	AHR
NlABCB7	GGSGTGKSS	PQD	LSGGEKQRVAIARA	LIFDEA	SSLD	AHR
NlABCB8	GTSGNGKST	NQE	VSGGQKQRIAIARA	LILDEA	SALD	AHR
NlABCB10	GSHGSGKST	SQE	LSGGQKQRVAIARA	ILLDEA	SALD	AHR
NlABCC2	GLVGAGKSS	RQN	LSGGQRARINLARAV	YLLDDP	SAVD	THQ
NlABCC3	GPTGGGKSS	IQN	LSLGQKQQICLARAV	YLLDDP	SALD	LHY
NlABCC4	GHVGSGKTS	IQN	LSGGQKQRVALARAL	YFLDDP	SAVD	THQ
NlABCC5	GRVGCGKSS	SQT	LSGGQKARVSLARAV	YLLDDI	SAVD	THH
NlABCD1	GPNGCGKSS	PQR	LSGGEKQRMACRQAI	ALLDEC	SAVS	THR
NlABCD2	GPNGCGKSS	PQR	LSGGEKQRIAMARLF	AILDEC	SAVS	SHR
NlABCD3	GSRDSGKTA	IQK	LSIPEKQLILIASSF	LIVQEA	AQES	TIL
NlABCE1	GTNGIGKST	PQY	LSGGELQRFACAMVC	FMFDEP	SYLD	EHD
NlABCF1	GPNGHGKTT	VQE	FSGGWRMRVSLARAL	LLLDEP	NHLD	SHD
NlABCF2	GLNGSGKST	AQE	FSGGWRMRIALARAL	LLLDEP	NHLD	SHS
NlABCG1	GPSGAGKST	MQE	LSGGEKKRLSIALEL	IFLDEP	TGLD	IHP
NlABCG2	GPSGAGKST	MQE	LSGGQRKRVSVGLEL	IFLDEP	TGLD	IHQ
NlABCG3	GPSGAGKST	MQE	LSGGQKKRLLVAVEL	MLFDEP	SGLD	IHQ
NlABCG4	GPSGAGKSS	QQD	LSGGQKKRLSIALEL	MFLDEP	TGLD	IHQ
NlABCG5	ATQVDEGRA	RSD	LTDSEAQRLSLACHL	LLLDRP	RSMD	IQP
NlABCG6	GPSGAGKSS	IQQ	LSGGQKKRLSIALEL	MFLDEP	TGLD	IHQ
NlABCG7	GPSGAGKST	MQD	LSGGQKKRLSIALEL	MFFDEP	SGLD	IHQ
NlABCG8	GPSGAGKST	TQD	LSGGQRKRLSIALEL	LFLDEP	TGLD	IHQ
NlABCG9	GPSGAGKSS	QQE	LSGGQKKRLSIALEL	LFLDEP	SGLD	IHQ
NlABCG10	GSKGSGKRA	SHR	LTLSEYRRLAIGIQL	LLLDEP	ANLD	MEK
NlABCG11	GPSGAGKSS	MQD	LSGGQKKRLSIALEL	MFLDEP	TGLD	IHQ
NlABCG12	GPSGAGKTT	TQE	LSNGQKKRISIGVEL	LFVDEP	SGLD	IHQ
NlABCG13	GPSGCGKTT	LQQ	LSGGEKKRANIACEL	MLLDEP	SGLD	VHQ
NlABCG14	GSKGSGKRA	SHR	LTLSEYRRLAIGIQL	LLLDEP	ANLD	MEK
NlABCG15	GPSGCGKTT	LQQ	LSGGEKKRANIACEL	MLLDEP	SGLD	VHQ
NlABCH1	GPSGCGKTT	PQE	LSGGQQRRVSFAVAL	LILDEP	VGVD	THY

Gene Sequence

NlABCA1

ATGACTCTGTCTCACCAGCTTCAAGCCATCTGGTGGAGAAATTTGATTTTGAAGAAAAGAGGATGGTTTGCTTCCATCTGCGATCTTTCTATTCCCTTGATCAGTCTTTGCCTATTAT

TCTATTTATATTTCAAGGGTGTCATAGGCAAAGAGCCCGGTGTAGTTCCGGAAATTGTATTCCCTAGAGAGGTATTTAATGAAGACTGTCTTCACTATCAAGGATATTCTAACGTTA

GTCTGCTATACACCAATCAAAATGATCCAAATGTCAAGGGAATCATGGAATACATCAACAGAACTAATTCCATCGATGGTCTTTTCGCTTATTTCAAATCAATTGGATTCGACAGT

GAACATGATATGGAAAATTGGGTACTTGCAAATATTAATGAAAGCGACTATAGGCAAATAGCGTATGGAATTGTATTCAACAAAGTGGATGAAAAGGAATTTTCATATAAATTG

AGATCTTCTTTACCGGACGATTATCCTAGTTTCAGATCAGAGGATGCAGGTCCAGATTTCTACACCGTAAAATGTTTCCTGGATTTGCAGAAAGGTCTTAGTGAAGGATTCTTAAA

AGTTCAAGGAAAAACAATTCCTAATAATATTGACTTAGGCATTCAAAGGTTCCCGGTTCCTCAATATTTGAAACCAGGTGCACTGTGGGAATTGCTGGAAACAGTGTTGACGTAC

ATAATGTTCATATCAGTTATGATCACTACAAATCTCCCAGTTCCAAAAATTGTTTCAGAAAAAGAAACTGGAGCCAAGGAGCTGATGCGACTGATGGGGATACGGCGTGAGGTGC

TGTGGGGAGGCTGGCTGATGAACATGCTCACGTTCGGCCTGGTCAATGTCACCTACACCAGCGTTCTACTCAAAGTGCAGATCAAAGAAGACTTCTCTGCTCTCCTCCCAAAGAC

AAACATCATTCTTCTCTGGATTGTTCTTGTGCTATTCCTTGTAACAATGGGCTTGTATGCTTTGTTCTTCTGCTGTTTGTTCAAGAAACCTCTATTGGCGAATTTTGTCGTAAAGCTGG

CACATTTGTGTCCGTTTTATGCCTTGGCTGGGTCAAAGTTATTGGATGACAAAGGAAAGTTGTTGAATATCTTGGCATGTATAGTGTTTCCACAATTCAACTGGAGTAGAACTGCAA

ATCTGATTGTTAGCTTCGAAAAACAATATGATGGTATAAGCTTTTTCAATCTTTTCAAAGAGTGTGATCAGGCTGCGGTCAGAATTGAAATGATTTGGCTCTTACTGAGTTACTTGC

TGAGTTGTGTCTTGTACTCCATCTTGATTTGGTATTTCGATAGTATAATGCCCGGCGAGTATGGTATTGCCAGACCTCTCTACTTCCCATTCCAGATGTGCTCTCGAAAGAAGAATA

ATGTTATAAAGGAAGATACGGATTTGAACGAGACTTTCTTTGAGAAAACAGGTCCCAACACCAAAGTGGGCTTGTCAGTGCGTAATTTGATGAAGGTGTTCAAGGGCGATGTGTA

CGCTGTGAATGGAGTCAACTTGGATGTCTATGACGGACAAATTATAGCTCTGCTGGGTCATAATGGCGCTGGAAAAACTACAACCATGTCAATGATCACAGGTATGTATTCTCCA

AGCTCTGGAAAAATCATCGCATCTGATAATGGTTCGACTTATAATATTTTCGATAATATGGATAAATTCAGAAAAAGTCTTGGATTGTGTCCTCAACATAATTTAGTAATCCCCTAC

TTGACAGTTTTGGAGCATTTAACCTTCTTTGGCATGTTGAAGGGATTGGATAAGAAACGAGCTGAAAGTGATGGTCTGAATTGGCTTCGTAGATTCAACATCCTTGATAAGAAAAA

CAACTTTCCACCAAAATTATCTGGAGGAATGAAGAGGAAATTGTGTCTTGCAATTTCACTGATTGGAGACCCCAAGATACTGATATTGGATGAGCCAACATCTGGCTTGGACCCT

GAATCAAGACGAGAATTGTGGGATAGTTTACTGGAGCTACGTCAAAATCACACGATCATCATAACCACCCATTTCATGGAAGAAGCTGACGCTCTTGGAGACCGAATAGCTATTA

TGGATCATGGCAAAATAATATGCTGCGGTAGTCCAATGTTTCTCAAGAAATTATATGGTACGGGATACAACCTACAATTGCTTACCGTTCCTAGTGCAAATAGGGACGCTATTACA

CAAATGATCAGGAGTGTTATACCAAACGGTTCATTGAAGACAAGCCAACAAGGTCAACTAACCTATTCCTTGCCAATTGAAGAATCGAAGAAATTTGCCGAGCTTTTTGAGAGCC

TTGAACAAAGCAAAACATCTTTCGGTATCACGAGTATTGGAATTTCGCTAACAACTATGGAGGAAGTATTTCTGAGAGCTGGCGTCGACACTTCTCAGCTTGTTCAAAATGATGGC

ATGGATGAGCCTGACGGCAATCGTGGATCGAATGGCAATTCAATGAGAAGAAGCTCAAGCACGGAATTTCTATCACAGAAAAAACTGAGTGGTCTGCCATTGATTGAACAGCAT

TTTGCATTAATTTTCAAGAAGAAGTTCCTTTGCTACTGTCGATCACTTTTCAGCAACTTGATTTACCTAATCATCGCTCTGGCATGTATATGTGTTGTTGGATGGACAATTAGAGGTA

CCAAAATGTCTGTCTCCACAAGATTGATAAATTTGGATCTGAATGACATGTACAGTCACACGAAAGTTGTGATAACGTATAAGGAGACGGGCGAAAAGTATGCCGAACTCTATA

ACAACTTGGTACCTCATGGAATGGAAAGACTCTACATCGATGAGAGTACATCAAAGACTGACATGAATCAGACACTGCTTGACATAGGCCGAGGAAATATAGAAGCCTACAAA

AAAGATTACGTGATTGCATCGAGTTGGGATTCAAAATCAGCAACAGCGCTGTACTCAGGTTTCAGCTACCACGGCTCAGCCATCTCAGTGAACATGCTGCTGAATGCTATCATCA

AGTCAACCGGACTGGAGACCACGCTCTCCGCTTCCTATCAGGCTCTTCCTCTCAAACAGTCTCAGAAGGATTCGCTCTGTGATCCCATGCAGAAAATGTCATTGTCGATGTCATTTT

TAATATTCATACCATTCGGACTGCTATTCTTCCTGGGAAATATGGCCTTGTTTCCATTACAAGAGCGATTGAATGATGCAAAACAACTTCAGCTGATGACCGGCCTATCTCCGATCA

CTTATTGGCTGGCAATATTCATCTGGGATCTGCTAATGTATGCTATCCTGATGGCATTAGCTGTTGGTGTGTTAGCAATATTTGATTACCCAATTGGCTTTGTGATAGCTTACCCGTC

TCTGCCGGTTTTCGTGTTACTGCTTTCTCTGTTTGGACTCAGCGGAATTGTGTATGCATACTTTTTCACGTTCTTGATGAGTTCCAGTGCAGCTGTGTCTCGTTTCGTTACCGTCAATGT

TTTCCTTGCTTTTGCTCTGCCTCTGATTGCTGTTATACTCAGTCTTATCCCTGGAATCGTCAACGAATTTTTAACGGATGCATTTTTGTACGCTATCCGGCTGATACCACCATGTTGCC

TGGGAATTGCCATGGTCAAATTCGCCATTCTCAGCTCCGATCATTTGTCTTGCTTGCACTGCAACCCAATGGGCCAATGTACAGAGGAACTGAACTATTTTGGATGGGATGAGAAA

CATCTATCATACGAAATCTTGTTCCTCGGAATCAGTTTCTTCATCTACGTGTTCTTCATTGCGTTGGTAGAGAGCCAATTATGGGAAATGATCTACGAGTATATCACTGGACTAATTT

ACAGCACAAAAATGGATGTAAGATCATTGGATGACGACGATGCTGATGTAAAAGACGAAAGAGATAAGGTTGACGCTGGCAGGAATGGTACAGGAGCGCCGACGCACCGTGTG

ATGGTGGTGGACGGTTTGGCCAAGCGGTTCGGGCGGTTCCAAGCGGTGCGAGGGGTGAGCTTCCAGGTTGGGTCGGGCGAGTGCTTCGGCCTGCTCGGAGTCAATGGAGCCGGA

AAGACCACCACTTTCAAGATGCTCACTGGCGCCCTCCTCCCCACTGCCGGCACTGCACGAATCACCAACTACAGTCTGCAAAATGATCGATCCAAGTACTTGTCGCAAATAGGAT

ACTGCCCTCAGTTTGATGGAATCAATGGTCTGATCACCGCCAGAGAAACACTGGTCCTGATTGGTCAGCTCAGAGGAATGCCAAAGCGAGTTGCTGAAAAACAAGCTGCCTATTG

GATCGATCTTCTAGGTTTGACGGAGTACGCAGACAGACAGTGCTGCAACTACTCTGGCGGCAACAAGCGGAAGCTGAGTGTGGGCATGGCTCTGATGGCCGACCCAGTGCTGCTC

TTCCTCGATGAGCCAACCAGCGGCGTCGACCCCGTTGCTCGCAGGAAGCTCTGGCTGGTGCTCGCCAAAATACAAAAGGCCGGGCAATCTGTGCTGCTCACATCTCACAGCATGG

ACGAGTGTGAAGCTCTTTGTAACAGACTGACCATTATGGTGGGAGGCCAAATGAAGTGTATTGGAAACATTCAGTATTTGAAACAGCGCTTTGCACAAGGCTTCACAGCCATTTT

AAAAATTCAACAAATGTATGGACAACATCAGCTGGAACGTGAGTCAACACTCAAACAAGAATTCTCCAGGAGGTTTGGCTCCTGTGAAATAATCAGCGAAACAATTGGAACTCT

TCAATATCACATTAAGAACACCGACATTCCGTGGTCTCAGCTTTTCAAAACAATGGATGATTTGAAGAACCAGTTTGATATTGTTGAGGATTACACGATCAGTGAGACAACTCTCG

AGCAGGTGTTTATTTCGTTTGCCAAAGAAGACAGGAGATGA

NlABCA2

ATGTCGTACTTTTTGCAATTGAAAATGCTGATCTGGAAAAATCTGCTTATAAGGAAAAGACAAAAGTTTCGATGTATCGTAGAAGTGATCTGGCCTCTCTTTCTTTTTCTAATTCTG

ATGTGGGTCAGAACAAAGGACTTGCGTGAAGACATTCATGAATGCCATTTTGTGCAGAAAGCCATGCCAACGGCGGGTGGTCTGCCATTTTTGCAGAGTTTTTTCTGCACGGCAA

ACAACACCTGCTACAAGTATCAGAACGATTCAGAAATTTCCAGAGAATCGATGAGCAGCTCATTATTGGTGAATCTGATCAACAGCTTCCAGAAGTTCACTGAACAAGGCGGTCA

GTCTCAGATGCAGAATTTGATCCTGGACAGCAGGCAGATGACTCAGATTGCTGCTAAACTTGCTGCCAGCCAGGGTGAACTGAAGCTGGGTCCCCTGATTAGGAATAGAACCGCC

TTCAAAGAAGACCTGGACGAGAGAAATGTGTCTCTTAGTCAGTCTGCCATCGACTCTCTTCTCTCTTCTCGTCTGGCCTTTCATTCTTTGTCACCGGACCAGTTGACATTGTTGAGAG

AGAACGCCTCAGTCATTTTGTGCGACGAAACACTGATGCAAGACGTCATAGTTGAACAGCAGCAGAATAATGGCAACAGTAGCGCTCTCTACGGTGAACTTTGCGAACTAAGCA

CCGACGAAGCTCAAAACTTTGTTGCGGCTGTCATAAGGCACAGCAATTCGGCCACATTGGCTCAACAGTTGCAGACAATGATGGAGACTGGTGCGGTGAAGTCGTTCACGTTTGA

AGACTGGGAAGAGGCGGGGATACTACAAAAGAGACTGCAATTCAACTATCAGAAGTTGATGTCATACAACTATGTTTTTGATGATACAATGGCGGTCTTTGAACAGTACCGTAAC

ATCTCGCAGAACTCCCAGAACTCATCTTGGGTGGAAGAGATTGTCGAAATCCTCAACGTGCTGCTGTGCGCCCGAAATTCAACCAAAGAGCAGGAGAAAAACTCACTAGATCCC

AGCGGAGAATCGGCCAGATTCGATGTACTTAACGAACAAATGAAGAAACGCATTGAAACTAAGTACGAGTATGACCCGTCCGTGTCCAAAGAGTGCAACGGTCTTTTCAAAATG

TTCGAAGAGAATCCGATTTTGGCCGGAGTGTGGAAAATCTACAAACCGTTCATCAGAGGTAAAATCCTGTACGCACCGGAATCGAATGCAACGTTGCGGGTCATGCAACGGCTC

AATGCAACCATCTATTCGATTAAAGAGAGCATCATGCTGGTTAATGAGACTGTACAACTGCTGCCACTAATTAGGATGGCACTGTTGGAGAATAGCATTGGAATGGAGGCTATCA

AGAAACTAATAGATTCCCAAGTAGGAGCATCAGTACTAAATAATACACTTGTGAAAGACTCATTGAATCTAGTAACCAGCGATAATGAATTGAAGGGAATGCAGGCGTTCGTTA

AAAAGTATCTTGACGATTCTAATGAAAGTAATAGGTCAAAGTCTTTGGAGGATGCTCAGAAGCTAATTGACAAACTTCTATCCTACGTTAATTGTATCGACTTTGACAAGATCAAG

CCTTTCAGTAGTGAAGAGTCGGCGACCAAGTATGGAATGAAGTTGCTAGAAAAGAATGAACTATTTGCTGTGATAATTTTTGAAAAACAAGATGGTGACCGATTGACTCCGTTTG

TCACCTATAAAATAAGGATGAGCTCGGATCGCGTGGACAACACCGAGTTCACACGTGACAGCCGCTGGCGCCCCGGTCCACGCATGCGCCCCTACATCGACCTCAAGTACCTCA

GCATGGGTTTCGGCTACCTGCAGGACCTCATCGAGCACTACATCATAGCCGAACACACTCCCCACCTCAACGCCTCCCAGCTGCCCGGCATTTATCTGCAGCAGTTTCCCTATCCA

TGCCACATCAACGACAAATTCATAATAGCGATCTCGGCCACATTTCCATTGTTCATGATGCTGTCGTGGGTGTACACTTGCGCGATGATTGTCAAGTCGATTGTCTACGAGAAGGA

GGAGCGGCTGAAGGAGACGATGCATGTGATGGGGTTGGGAAATGGCATCCATTGGGTGGGCTGGTTCATAGACAGCATTCTGCCCATGATGCTCACCATTCTCCTGCTCACGCTT

ATACTCACCTATGGCAAGATATTGCGCAGCGCTGACCCGACTCTGATCTACTTCTTCCTTCTGATCTACTGCGTGGCGACGATTGCGCAGTCGTTCCTGATTTCAGTGTTCTTCTCGC

GCGCGAACCTGGCCGCCGCTTCGGGTGGCATCATATTCTTTGTGCTCTATCTGCCCTATCCGTTCATGGTGCGATGGATGTCCATACTGCCGCCCTACATCAAAGCTATTATGTGTCT

ATCATCGAACGTAGCATTTGGAGTGGCGACTTCCTACTTTGCGTTCTACGAGGAACAAGGCACGGGGGCGCAGTGGGCCGAAATCAACTCCAGTCCACTCTACGGCGACAAGTTC

AACCTGCTCTATGTCATCTATCTGCTCCTGTTCGACACCTGTATCTACCTCCTGCTCACCTGGTATCTCGAAGCTGTCATGCCTGGTCTGTACGGTATTCCAAAGCCGTGGTACTTTC

CTTTCACAACTTCCTATTGGTGTGGAACCACTAAAGGAGTGACGGACATCAATGTACTGAGAACAGGCAGTATAGCGAACGAGAACTGTGAAAGCGAGCCGCTGAAACTGAAGC

AGGGTATCACCATCCAGAGTCTGTCGAAGGTCTACTCGAACGGCAAGGTGGCTGTTCGTGACCTCTCACTCAACTTCTACGAGGGACAGATCACCTCGTTCCTCGGTCACAACGG

AGCCGGCAAGACTAGCACTATTTCGATGTTGACTGGCCTCTTTCCTCCATCTAGTGGTACGGCAAAGATCTACGGCCTCGATATAAGAACAGACATGGATGCCATACGTAAAAGC

ATGGGCATGTGTCCTCAGCACAATGTGCTATTCAACTTACTGACAGTCGAGGAGCACCTGTGGTTCTACGCGCGTTTGCGAGGCGGTCGCGCCGGTGACGGGAGCGGAGGGGAGA

CCGTGAAGCGAGCAGTGCGCGCCGAGTGTGATCAGATGATCACGGACCTGGGTCTGCCGCACAAGCGGCGATGTCTGGCCAGTCATCTTAGCGGCGGAATGCAGCGGAAGCTAT

CGGTGGCCATCGCGTTCATTGGCGGATCCAAGACAGTGATTCTTGACGAACCGACCTCAGGAGTTGATCCTTATTCGAGGAGATCTATATGGGAACTACTCACCAAGTACAAGAA

TGGACGGACAGTAGTGCTGACCACTCACTATATGGATGAAGCCGACATACTTGGTGACCGTATTGCAATAATAGCACATGGCAAACTGCAGTGCTGTGGCTCTAGTCTCTACTTG

AAGAACCGATTCGGCAGTGGCTACTATCTCACCATTGATATCAGGGAGGACAAAATTTCTTCGGGCCTCTCTTCTCCAGAAATAAGAAAAATCACACAACACATCAAAGAGATA

GTGCCTTCAGCCAACTTCCACGAGCACATAGGCACAGAACTTATCTACGTGTTATCGCATTCTGACTTGGAACGATTCAAGAAGCTGTTCAGTTTCCTTGAAGATTTCAAAACTGA

TCTATTCATCAATTCATATGGCATTTCTGACACGTCTCTTGAAGAGATATTTCTACGTGTGGGTTCTCAGAATGATGAAGCTATGGATGCTGATGACGAAAGAGATTCATCATCATG

CTTCAATAAATTGACAAGTTTCTTCTATAAGAAGAAACCAACTAGGAACTCTTGTGTACACAGGCCCGATGACCAACATACATCAGTGCCAGTAATCGATGAAGGTGAAAACATA

AAGCCGGAAGGGAACAAACACTGGAGACAGTTCCTGGCTCTGCATGTGAAAAGATTCCATCATACTAGAAGAAACCGGAAAGCGATCTTCTCAGAGTTAATCCTGCCGGGCCTG

TTCGTGTGCATGGCTCTGTTGGTGACGTCAATCGTGCCGAAACTGCAGCAGCGACCACCGCTGACGCTGGCGCCATCTGTGTACGCCCCGCCGCACTACACCTTCGTCAGCCTCCA

AGGTGTGACGTCACCGGAGGGCAGCTGGCAAGCGGCAATTGATGCCAACCTCACTGGGCCGCTAGGACTGGGCACCGCCTGTCTCAACAGTGAACGCAGTTATGACGAGACCTG

TGCAGGCAAGTCGTACGACTTTACGCCGGGCGACCTGAACGTGCCGTCGTTTGGAGCGCCCTGCTCGTGTGACACGGGCGCTCAGCTTTGTCCCTCCGATCCTGAAAGGCCTGTAC

CGCCATCTTTAAAGTTATCATCCCATGACGTTATGTTCAACCTGACTGGAAGCAATATATCGGATTGGATTCTGAAAACTTGGAAGCAGTATCAGAAAACGAGAATGGGAGGTTA

TTCGTCAGGTTTTGAGTCACCCGTGCCTGTAGTAGAATTCAAGTCAACCAATATTTCACCCATGGACTTGACTCAGAACCCCTACGTGGACATTCACTATAAAACTAATAATGTCA

AGGTTTGGTTCAACAACAAGGGCTGGGCGTCTTCCGTTTCTTATATGAATGCCATCAACAACGTGATTCTGCGCAGTAATCTGCCCGCCAACCAGTCACATCTGGCGCACCAGTAT

GGCATCAGAGCCATCAATCATCCCATGAACTTCACCGAAAAACAGATGAACATCGAACTAATCAAGGAGAGTGGAAAATCACTGCTTCATGCCATTTCGGTGTTGTTTGCTCTGA

GCTTCGTGCCCGCTTCGTTCCTGGTCTACCTGATCGAGGACAAGGTCTCCAACAGCAAGCATCTGCAGATGGCATCTGGTGTCAACCGCCTCGTCTACTGGTTGCAGGCGTACGTC

TGGGATATGTGTTGCTACCTGTTATCAGCCGGCCTGTGTGTGTTCATATTTCTGGTTTTTAACGAGCAAAACTATGTGTCATCAAGAAACTTGGCTGGTTTTGTTATTCTACTCACCTT

CTACGGGTGGTCGATAATTCCGCTGATGTACCCGGCGAGTTTCGTGTTCTCTGTTCCGAGTTTTGCGTTTGTGGGCTTGGCCTGTGCTAACATATTCATTGGCGTCATTACCACTGTC

ACAACTTTTGTGCTCGATGTATTTGAAGATGAAGACTTGAGAGCAGTTGATGACGTATTGAAGGAGGTGTTCCTCATATTCCCTCACTTCTGCTTGGGAGACGGCCTCATGAAGCT

GGCAGCCAATCACATCTACTATGTTGCCCTACAACAGTACATAAATATCGAAGTGGGAAATGAAATCTTCAGATGGGACTGCTTGGGCAAGAATCTATTCTGCATGTTTTTATCTG

GCATTTTCTACTTTCTGCTGACTTTGGTGCTCGAATTCCGAGGTCTGCCGTTCTTGACAAGCAGTTCGGCTGGCGGTGCTAAGAATGTAGTTGTTAGTCCTGAGGACGGATTGGGCA

AGGAAGACGAGGATGTGCGTGCGGAACGCATTCGTATCAATCAAAATCAAGCCATTGACGACATCCTAGTCATCAAAAATCTCACCAAGGTATATTCGCGTTTCAAAGACTCTCG

GCCGGCTGTAAACCAGGTTTGTGCCGGAATCAAGAGGGGAGAATGCTTTGGACTTCTTGGTCTCAACGGTGCCGGAAAAACTACCACCTTCAAGATGCTAACGGGCGCGCTGAG

GCCGAGCGGAGGTGACGCTCTGATTGGAGGATGCAGCGTGTCACGTGACCTGGACAGGGTGCGCGCGCAGCTCGGCTATTGTCCGCAATTTGACGCATTGGATCCTTTGCTCACA

CCCCGCGAACATTTGGTTTTCTACGCGCGGCTTAGGAATGTGCCTAGTCGGCAACTGCAGTCGGTGGTGGATGCCAGTCTAATGAAGCTGGGTTTAGGTCATTATTCGGACAGATG

CGCCGGCACGCTATCTGGTGGCAACAAACGGAAGCTCTCTACTGCCATTGCTGTACTTGGCAATCCTCCTGTCATATTCCTTGACGAGCCGACGACAGGCATGGACCCGGTGGCG

CGTCGCTTCCTTTGGCAGTGTGTACAGCGACTGACTCGCGAGGGAGGCCACAGTGTCGTGCTGACGTCACACAGCATGGAGGAGTGTCAGGCGCTCTGCACTCGCCTCACTGTCA

TGGTCAACGGCCAGTTCAAGTGTCTCGGCTCCAGTCAACATCTCAAGAACAAATTTGGCGGCGGCTACAGTTTGTCGCTGCACTGTGCTGATAGCGGTGGTGAGGGTGGCGGTGG

AGGGGGAAGCACCAGCTCTGACACAGTGGCTGATGTCAAGAACTACATCAGCAGCAAACTGCCTGAGGCCTCACTACAGGAGCACCATCACACTCGGCTCAGGTATCAGCTGGC

TTCGAAGCAACAGTCATCCCTCCTAGCTCACGTGTTCCAAGTGATGGAGGAAGCGAGAGCCACTGGACTAGTCTTGGACTACTCTCTTTCTCAGACCACTCTCGAAGATGTATTTTT

ACAATTTGCCAGTGAACAAGGCGAAGCAGATGAAGATTTGAATACAAGGCACAGACCTGCCATCATACGGAATTGCTTCAAATGCTGCTCAGCCACGGTGCGATCTATGTCTAAT

AGACTACTTAAAAGGATTTCACTGCAAATTCTAAGTTTTTGGAAAGAAGGCTCGGTATTTTGGCACAGCCCAACCGGATCAAAACGATGTAA

NlABCB6

ATGCTGTATTGTCCAGAAGGCATATCTCTAATCCAGATCTGGCTGAATCATGGCATCTCGGAGTGCTTTGCAGCCACGCTCTCCTCCTACCTCCTTGGAGTTTATATTCTACTATTTG

GAACCATGCAAGTTTGGATGTACAAAAGGTATGGAACTCTGGCTTCCGCTCCGCTGCACCAATCAACGCTCTACTATCTCCAGTTCTTTCTCTGCTTTTTACTCAGTGCGTTGGCATT

GAGCAGATTTCTATTGCAGGTTTTATTACTGAATCCAGGCGCTATTTATGGATATATGTTGATTTGGACCAGTGTCAGTGTTCTCATCTATCCATATTCGGCTGTGCTGATCTGGCTT

GAATGCAACTACCATCTTCCTTCTGTGCCACCCAAGGGTCATGGCATAATCTTGCTTGTCTTTTGGACGCTGAATTTTATCACAGAGAATCTAACTTTGTTGAACTTGGCTCGACAT

GACTGGTGGTTTCATCCAACAAAACTATCGGACAAAATAGAGATGGCCTTATTTGTGGGAAGATACGTCACCTGCTTACTGCTGTTTGTTCTTGGACTTCGAGCACCTGGTATTACC

ACGAGGGATTACTTCAATGCGCATGAGTATACCAACCGAATTCTGCCTCCTAACAGAAGTAACTCACAAAGATCTTCTTCGTGGCTGGGAAATGCATATCATCATATGTCCGTATT

ACTTCCATTCCTTTGGCCAAAGAAGAGTACTGTCCTACAAATCAAGCTGATTATTTGCATATTGCTGCTATTTATTGCGCGAATAATAAACGTATTTGTTCCTCTCTACAGTAAATAC

ATTGTCGATAGCTTGAAGACAGTGCCTCTGACATTCAGATGGGATCTGGTTGTAACCTTTGTTGGAATGAAGTTCCTTCAAGGTCAGGGCACCGGAGGAATGGGACTCTTGAATAA

TCTGAGGTCGTACCTCTGGCTCGCAATTCAGCAGTATACATCTAGAGAAGTGCAGGTGAGTCTGTTTGCTCACCTGCATAGTCTGAGCTTGAACTGGCATCTGAAGCGGAAAACTG

GTGAGGTGCTAAGAATAATGGACAGAGGAACTGACAGCATCAACAGCCTGCTGAGCTCCCTGTTCTTCAGTATCATTCCGACTCTCATTGATATTATCATCGCTGTCATTTTCTTCG

CCACATTCTTCAACTCTTGGTTTGGTGCTATTGTATTCACCACCATGGCGCTGTATCTAGCTTTCACAATCATCATCACTGAATGGAGAACCAAGTTTCAGCGTAGCATGAATCTGG

CTGATAATGCCTGCAAGGGAAGGAGTGTGGACTCGCTTCTGAACTTTGAAACAGTCAAGTACTATGGCGCTGAAGCATATGAGGTGGAGGCCTACAGAGAGTCAATCCTTCAATA

CCAGGAAGAAGAATGGAAGAGTGTTGTGTCACTGCAACTTTTGAATACGTTGCAAAACACTGTAATATCAGGAGGACTGCTGGTGGGATCATTGTTCTGTGTCTACTTGGTCACAA

TAAAACAGGAGCTGACGGCTGGTGACTATGTTCTTTTTGCGTCCTATTTGATTCAGCTCTATGTTCCTTTGAACTGGTTTGGAACGTATTATAGAGTAATTCAGAAGAACTTTGTTGA

TATGGAAAACATGTTGGACTTGTTGTCGGAAGAAGCTGAGATTCTGGATGCGCCTGGAGCAATGCCATTGGCAATCACGAAGGGAGCGGTCGAGTTCAGAAATGTTTCCTTTGCTT

ACACTGAGGAAAGACTGATCCTCCGAAACATTTCATTCACTGTACCACCTGGAAAAACAGTTGCCTTGGTTGGTCCTTCAGGAAGTGGAAAGAGCACCATAATTCGTCTTCTTTTC

CGTTTCTACGATGTGGACCAGGGCGCCATTCTGGTGGATGGGCAGAACACGAAAACAGTGAGGCAGTCATCTCTCCGAAGAGCAATTGGCGTTGTGCCCCAAGATACGGTTCTCT

TCAATAATACAATCAAATTCAACATCCAGTATGGCAGGGTGGCTGCCCCTGATGCTGACATCATTGAAGCTGCCAGATCAGCTGACATCCATGAGAGGATCCTCACATTCCCCGA

AGGATACAACACACAGGTCGGTGAGAGGGGATTGAAACTGAGTGGCGGGGAGAAGCAGCGGGTTGCAATAGCGAGAACCATTCTCAAATCGCCAATCGTTGTGTTGCTAGATG

AGGCCACCAGTGCGCTCGACACCCAAACTGAGCGCAACATCCAGTCTGCATTGTCTCGAGTCTGTGCGAACAGAACCACCATAATAGTTGCGCATAGGCTCTCCACAATAATCCA

TGCCGATGAGATTCTAGTCCTCAAAGATGGAGAAATCGTAGAGAGAGGAAGACACGAAGAGCTGCTGAACTTTGGTGGCATGTACCACTCGATGTGGGAGGCTCAGCTGAAGAA

CGACGACATCCAAGCTGACAGTGACCAGGTGAATGCTGCAGGCGACACGGATCAAAGCTAA

NlABCB7

ATGTTGATTTTGTGTTTGGACTCAGCTTTTAGAAATTCTATAAAAAGATTGCCAAAAACTACACTATCTGTACAGTTCTACTCTAAATGTTCTAAAGTAACAATATCTACTGCCTTCA

ACTTGGGGAATAATTTTCATTTCAAGAATGGAGAAAATTTGTCAAGAACAGTGTTACATAATTTTAGACGATATGCCGCTAGCTGTGATTCAAAAGACTCGAGAAAAGATAAAAG

AAAACAAAATGTATTGGTTGATAACGTAAAAGGAACTGTTATTGTACCTAGTCATGGTTTAAAAACTAAGGGGCTGAAATCATTCTTTCCTTTTCGACAGTCAAAAAGAGATTGCT

TTCATCCTGGGGTATCAGCGCTCGACAGGACTTCAATCAAGGTTGGTAATGAAAATGATGTGACAGGGAAAGACATGATCAAAGCTATGTTGACTTACATCTGGCCAAAAGATGA

TCAATCTATACGTAATCGAGTATCTGTAGCAGTAGGTCTCCTAGTTTCTGCTAAATTGATGAATGTGGCTGTACCATTTTTTTTCAAGTATGCTATAGATGACTTGAACTCAAAGGTG

GCTGCTGCTAACAACGGGCAAGCACTTTTGGACTTTGCTACTCCCACCGATGCGGTACTGACGACTTCTGTTGCATTGCTCATTGGGTATGGTATTGCCAGGGCAAGTGCAGCTGG

ATTCAACGAATTGCGCAATGCAGTATTCGCAAAAGTTGCTCAGAATTCAATCAGACGAATATCGAAGAACGTATTTCTCCATCTACACAATCTGGACCTCAGTTTTCATCTGTCTA

GACAGACCGGCGCGTTGTCTAAGGTTATAGACCGAGGTAGCAGAGGTATAAATTTCGTTCTGTCTGCAATGGTATTCAACATTGTTCCTACAGTCTTTGAACTAGCATTGGTGAGC

TCCATATTGGGAGTGAAATGCGGCGGAGAATTTGCAGCAGTTGCTTTAGGTTGTGTGGGAATCTACAGTGCTTACACATTCTCAGTTACCAAATGGAGAACAAAGTTCAGGGTGTT

TATGAATAAAGCTGAAAATGAAGCTGGCAACAAAGCGATCGATTCACTCATCAATTATGAAACAGTGAAATATTTCAACAACGAACTGTACGAAGCGAAACGATACGACGAAT

CATTGAAGAAATACGAGGAAGCCTCTCTGAAGACTAGCACCAGTTTGGCTATGTTGAATTTTGGGCAGAACGCAATTTTCAGCAGTGCTATGGCGCTGATAATGGTGCTTGCGGCT

CGAGAAATATCTCAGGGTCATATGACAGTCGGTGACCTAGTAATGGTGAATGGCCTGCTGTTCCAACTCTCTTTACCACTCGGATTTCTGGGGTCAGTCTACCGCGAAGTGAGACA

AGCGCTTATCGACATGAAAACAATGTTTACATTGATGGCCAAAGAGCCAGAGATAAAGGATTCTCCTGGGGTATTACCTTTGGTTCTGAAAAACGACAAAAGTATCGAATTCAGA

AATGTTTGTTTTCAGTATGTTGATGGAAAACCGATATTCAATGATTTATCTTTTGTTATACCTTCGGGTAAACACTTTGCAATAGTTGGTGGATCTGGTACAGGGAAATCGTCGATG

ATAAGGTTGTTGTACAGATTCTTCGAGCCCGACTCCGGAGAAATCCTGATCGGCGGTCAGGACATAAGTATGGTCGATCTACAAAGTTTGAGGAAATCAATCGCGATTGTACCTC

AAGATACTGTACTGTTCCACGACACCTTGTTCTACAACGTACACTACGGCGACTTCTCGAAAAGTGAGGAGGAGGTGTATAACGCGGCGGTGATGGCAGATCTTCACGATTCGGT

GACCACTTGGCCGGAAGGCTACAAGACACAAGTTGGAGAAAGAGGACTGAAGCTGTCCGGAGGAGAAAAACAGCGAGTCGCCATTGCCAGGGCGATTGTCAAGGACTCCCCAA

TTCTGATATTCGATGAGGCCACATCTTCGCTGGATTCGATCACGGAACAGAACATCCTGAACACGGTGCGACGTGCCACCAAGGGCAGGACGTCGATTAGCATCGCGCACAGGTT

GTCCACTGTCATGGATGCTGATGAGATTCTCGTCCTGGAGAACGGCAAACTGAGAGAGAGGGGAACTCACGATCAGCTGTTGAATTTGCCCGACTCACTGTACAACCACCTGTGG

CAGCTGCAGCACTCTGTTGGAGTCGAGGATGAACCAAAACGGAAGGTGGATTCAGCCAAACAAGGGTGA

NlABCB8

ATGTTATGGAGATTTGTTTGTAACCCTTCTCATCAAACTAATTTTGGCAGTGTAAGGAAATTATGGCATCAATTGAGCAGAGAAGTGAGATCAACCAAGTTTTCAAAAGACTTGAA

AGCGAAACCATCAGAATGTTCGAGTGGAGCTCGACTGACGGGGAGACCTGCCATTCAGATTTGTGTGGTAGCATTCACAGGGATCGGCATCGGAGTAAGGAGCCTGGCAGGCAT

CCCAAGAGCTGAGTGCGCAGGTGTTCGCTACATTGAAAGAAAGAAAAATTCGGAAGCTAAATTCAGTTGGAGTCAATTTTTCAAACTTCTCTGGCCAGAAATATGGAGTCTTCTG

ATTGCAGTTGGCGCTGCCATCATTGTTGCAGTTCTCAACGTGCATTTGCCAATCTATCTCGGAGGACTGATCAATGTGATGACAAGATTTTTAAAGGATAACGAATCTGTGGATTAC

GTAACAGAAATGAGAGGGCCTGTGATTCGCCTCATATCTCTTTATCTACTCCAGTCTGTAGCCACATTTGTGTACATATCGGTTCTTTCTGGAGTTGGAGAGAGAATAGCAGCCAG

CATGAAAAAAGAGGTGTTCGCCTCTATTATGCGTCAAGAACTCTCTTTCTTCGATAAGGAGAGAACTGGCGAGCTAATTGACTGTCTCACAACAGACGTACAAGAATTTAAAAGT

GCATTCAAGCTATGCGTTTCTCAAGGATTAAGGAGTCTGACTCAGATTGGTGGGTGTGTGGTGTCGCTATACTTTATCTCTCCAAAAATGACTGGGATGATGGTTGTTGTTGTTCCA

GCAATCATTGTAATTGGTACAACGCTGGGGGCCATTCTGCGTGGCATATCTAAAGAAGCTCAAAAGCAGGCGGCGAGAGCTATAACAATAGGCGAAGAAGCAATAAGCAACAT

TCGCACAGTGCGTGCTTTCGCCAACGAAGACAAGGAATGCGAAATATTCGGCGATGAGGTGGATGAGAGCGCTCGTTTGCATGAGAAATTGGGATTTGGAATCGGATTGTTCCAG

GCAGGTACGAATTTGTTTCTGAATGGAATAGTTTTGGGTACACTGTATTGCGGTGGCTACCTGATCGTATCAGAGCAAATGACAGCCGGTGATCTGATGTCGTTCCTAGTTGCCAC

GCAAACAATTCAAAGGTCGATGGCCCAGTTGTCGCTGCTCTTCGGCAATTTCATCAAGGGTCTTCAGTCGGGAGCTAGGATTTTCCAGCTTATCAACCAAGAATCGAGTATTCCAA

TCGCTGGAGGAAGTACTATACCTTATCATTCACTCATAGCTAATGTCGAATTCAAGGACGTAACATTCTCATATCCTACACGACCAGAACAGGTAATTCTGCATAATTTCAATCTG

ACTTTGCCTGCCGGCAAGACTGTAGCCATAGTTGGCACTTCTGGTAACGGAAAATCAACAGTAGCAGCATTACTTGAAAGATTTTACGATGTGGATGAAGGTTGTATTACGGTTGG

AGGTGTGGATATACGTGAATTGGATCCAAAGTGGCTGAGAGGCAGAGTCATTGGTTTGATCAATCAGGAGCCTATTCTATTCGCCACATCTATCATGGAGAACATTCGCTATGGA

AAACCAACAGCGAGTGATGAAGAGGTTTTTGAAGCAGCAAAGAAGGCGAACGCTGATGGCTTTATTAGTAAATTTCCTCAGGGCTACAATACAGTGGTTGGGGAACGTGGAATT

ACTGTATCGGGAGGCCAGAAGCAAAGGATAGCAATAGCTAGAGCTCTTCTGAAGAATCCTTCCATCTTGATTCTGGATGAAGCCACAAGTGCATTGGATGCTGAATCCGAAAAA

GTTGTACAGAAAGCTTTGGATGAGGTTTCAAAAATGAAGACAGTGTTGGTCATAGCCCACCGGCTCAGTACTATTCAGAATGCAGATTTGATAGTAGTTCTGAAGAATGGTGTCAT

TGTGGAGAAAGGGAATCATGAAGAACTGATCAGGAGAAAAGGACATTACTGGAATTTGATGAACCAACAAGATGATCAAAGGACGAAAGGTTGA

NlABCB10

ATGTGGTGCGGCATATATCCGACACTGGTTTTCAACAGTCGCTCAAAACTGAAGATGATTTTCCTACCGATGCGGACTTTGAGTTGGGCTAGCCGTCTGCCGACTGTCAGCAGACG

GACGTCTGTTATGCTCTGCAGACCGTCTGTTGTGCCGAGGACATCTGTGATCGGAGCGATCCTCTGCAGACGGTGCGCAGACTGTGCCAGGCCCAATGTGTGGCTGTTACGGCAAC

CGTACACTTCGCGTCTGCAGAGTTCTGTTTCTAATAATTCGTGGTGTTTGTTTTCGAATCGTTTGCTAGTGCAGAACTCGAGGCGGTATGTGGCTAACAGGCCCGTGTTAGATGCGG

CAGTCAAAACTCAGAAAGTCTCCCTAGGCGGCCGTGAGATGAAGAGATTGCTCTCCTTAGCCAAGCGGGAGAAATGGAACCTCACTAGTGCAATATTCCTCCTTCTGATCTCAAG

TGCAATCACAATGTCGGTGCCATTCGCTCTAGGCAAAGTTATAGATCTGATCTACACGTCGGATGTGGTCAAAATGAAAGAGAATCTACTCAAGCTCTCTCTAGTGTTGTTTTGTGT

TTTCCTTGTTGGCGGACTTTGCAATTTTGGACGTGTCTATTTGATGAATGTCTCAGGTCATCGTATCACAAGAGACCTGCGTGAGAAGGTGTTCCGGTCGATAATGAAGCAAGACAT

AGCGTTCTTTGACAGCAACAAAACGGGCGAACTGATCAACAGGCTATCAGCTGATTCGGCGCTGGTCAGTCAGTGTGTGACCATGAACATCTCAGACGGACTTCGGTCAACTGTG

ATGGCCTGTGCTGGCGTTTCTATGATGTTCTACATGTCTACAGAGCTGGCTCTAGTTGGACTGGGAATAGTGCCTCCTGTAGCTGCAATTGCTATAGTTTATGGACGATTTGTTAGA

AAAATAACCAAATCCGTGCAAGGAGCACTGGCAGAGTCGACGCAGGTGGCCGAAGAGCGTATCAGCAATATTCGAACTGTGAAGGCTTTCAGTCAAGAAAGGAAGGAAATTCA

GGCCTACAGTGAGAAGATGGACAAGGTACTCAACCTCTCCATTAAGGAATCGTTGGCTAGGGGAGGATTCTTCGGAATGACAGGCTTCAGCGGCAACGTGATAATCCTGACAGT

GCTGTACTACGGCGTATCAATGGTGGCCAAACAGGAGTTCACAGTTGGCAATTTGTCTGCCTTTCTGTGGTACGCTGCCTATGTGGGAGTCAGTCTAGGAGGACTGAGCAGCTTCT

ATGCCGAGATGAACAAGGGTCTTGGAGCCAGCACACGGTTGTGGGAGCTTATAGATCATGTGCCCGGCATACAGGATACTGGTGGTCTGATACCCAGTGAACCATTGCGCGGTG

ATATTAAGTTCAAAAACATCACGTTCTCCTACCCAAACAGGCCTGAGAGCACAATTCTAAACGGCTTCAACCTCAATGTGGACAGCGGATCTATTATAGCAGTTGTGGGACATAG

TGGATCTGGGAAGTCTACACTCTTCTCTCTACTAATAAGACTGTACGATCCAAGCTCGGGTATCGTTCTCATAGATGGAATGCCATTGCAGTCTTTCAACCTCTCCTGGCTGAGATC

GCATATTGGAGTAGTCAGTCAGGAACCAGTATTATTCTCGGGAACAATTCGTGAGAACATAGCCTACGGGGTTGATGACCCTTCCAGTCTATCGAATGAAGACATAGAGATGGCT

GCAGCCGAAGCCAATGCTTACGACTTCATCACCAGAGACTTTCCTGAGGGATTCGAGACCAGGGTGGGAGAGAGAGGCATCATGCTCTCAGGAGGACAGAAGCAGAGGGTTGCT

ATCGCCAGAGCATTAGTCAAGAATCCAAGAATTTTATTGCTGGATGAAGCTACCAGTGCCCTTGATGCAGAAAGTGAACATTTAGTACAAGAGGCTTTAGAAAGAATAATGAAA

GGACGTACTGTTCTTACGATAGCTCACAGACTGTCGACTATCAAAAATGCAGATCAAATAGCCGTATTAAAGGATGGAACTGTGGCACAAGTGGGAAGTTACGATGAACTGATG

AGAGATGAGTCCGGAGCTTTCAAGAAACTTGTGCAACACCAGACGTTCCAAGCTACTTCCCCGATCTTGAAAGATGTTGAAATGTTGAAGGTTCTCTAA

NlABCC2

ATGGAGAAGGATAAGAATGAGAGAGATGAGAGGCTTCCACATCCGAGGGCGAAAGCCAACTTTCTGTCCGCTGCTACTTTCAGCTGGACCTTCCCTATGTTTCGTTTGGGATATA

AAAGAGACCTGGAGGTGACTGATTTGTATGCAACACTCAAAGAACACTCATCGAATAATCTGGGAGACAAATTTGAAAGGAAATGGAACGAGGAATATTCAAAGGCAACCAAG

AGAAAAAAGGAGCCCAGTTTGACGAAAGTGGTGTTCTCTGTATTTGGAGGAGACATCGCTTTCTTTGGATTCTTCTTACTTTTCATTGAAGTGGTTGTAAGGGTAGCCCAACCGCTG

TTACTAGGCAGACTGATCAGATACTTCACACAGAAGCAGATGTCTGCGGAGGAAGCTGGGCAGATGGCCGAAGCACACGAACTGGTGACGGAGCGGGACGCCTATCTGTATGCA

GTGGGGCTGATTGCATCGTCTGCACTTTCCGTCGTCATGATCCATCCCTACATGATGGGAGCCATGCATATCGGCATGAAGATGCGGGTCGGGCTCTGCTCGCTCATCTACAGAAA

GGCACTGCGATTGTCGAAAACGGCACTGGGTGAGACAACAGTGGGTCAGATTGTGAACCTGTTATCGAACGACGTGAACCGGTACGACATTGCGTGCATATTCGCGCACTACCTG

TGGATCGGGCCACTCGAAACTGTCATTGTCACCTACTTCCTCTGGCAGGAGATCGGCGTTGCGTCGCTAGTCGGAGTGGCTGCGCTACTGGTTGCTATCCCGTTACAAGCCTGGCTT

GGAAGGAAGAGTTCCGTATACCGTTTGCGAACAGCCATCAGAACCGACGAAAGAGTACGACTGATGAACGAAATCATTTCTGGCATTCAGGTCATCAAAATGTACGCTTGGGAA

AAATCTTTTGCCAACATGGTTTCTAATGCTCGCAGGAAAGAACTGAACGAAATACGCAAAACATCTTACGTGAGAGCCCTGTTGTTATCATTCATAATTTTCCATTCAAGACTGGC

TGTGTTCATGAGTATACTCGCCTATGTTATCACTGGGTCGAGTATTAATGCTGAAAAGGTGTTTACTGTGACTTCGTTCTTCAATATCTTGCGACAAACCATGACAATATTTTTCCCG

CAAGGTATTGGACAGATTGCCGAAGCTATGGTTTCAACCAAACGATTACAGAAATTCATGATGTACGAAGAAATCCAGAATACTTCGTACACTTCTGCAGCGACCAACAACAATT

CCGAGGATAATGGCAAACGGATGAACGGTGTGGACAAGACAAAGAGCGACAACACTAAGGAGAATGGGACCAGTAAGGAGAACGGACCAATGGGGGTTAGTGTGACCATTAA

TCCCAAGGAATCGGGGGAAGCCGCCATTGTAATGGACAAACTCACTGCAAAATGGACACCGGATCTGACCGAGAACACCTTGAATAATATCAATCTCGAACTGCCGCATGGCAG

TTTGATGGCTGTTATTGGATTGGTCGGAGCTGGCAAGTCGTCGCTGCTGTACGCGATCCTGCGCGAACTGCCGCTGTCGTCCGGTTCGCTGACTTTAAACGGCGGCCGACTGTCGTA

CGCGTCACAGGAGCCGTGGCTGTTCGCGGGCAGTGTTCGCCAGAACATTCTGTTTGGCGAGCCGTACGATCGCGAACGCTACCGGGAGGTGACGCGCGTCTGCGCACTGCGACCC

GACCTGGAAATGATGCCCTACGGCGACAGGACGGTGGTGGGTGACAGGGGTGTGTCGCTCAGTGGCGGCCAGCGCGCCAGGATCAATTTGGCCAGAGCAGTATATAAGAAAGCT

GACATCTACCTTCTGGATGACCCTTTATCAGCAGTCGATACTCACGTCGGAAAGCATTTATTTGAAGATTGTATATTGGGTTACTTGAAAAACAAAACACGGATTTTGGTGACGCA

TCAGCTTCAGTACTTGAACAATCTTGAGCACATTATTATGTTGGATAATGGAAACATATTAGCGCAAGGCAACTACAAACACATCCAGTCATCAGGAAAAGATTTGGCCAATCTG

ATGGCAAAACATACGGAGAGTGATGACTCTGATCCAACTAGTAGTCACGTTCAGAGACAGTCCTCAGTCCAGAGTATCGCATCCTCGCTGGATGACTCGAAAATGCAAGAAGAG

CCGCCCGAAACAAAGGAGACACGCAGCGTCGGTACAGTGGGTGGAAGGGTGTACAAGAGCTACATCTCTGCCACCGGAAACTTCTTCATGGTAATCTATTGCCTGCTGCTCTGCA

TTCTCACACAGCTGTTCGGATCTGGAGGCGATTTCTGGATTACTTACTGGGTCAATCTTGAAGAACAGAAACTACTTCGTGAGCAGCAGGGCGTCGATATTCATTTGAATCTGACA

ACAAATTCAACATCAGAAGTGACCAAATCGAATAACGGGACGTACATTTATGCTGATGGAGATTACCTACTGAGGACCACTCCATGGTTCGACTTGACTCAGCGGACCTGCATGT

ATATCTTCACGTTTTTTATCGCAGGTGTTGTTTTGGTGACTCTGATCCGTTCGTTCACGTTTGTGCGCATGTGCGTAAGAGCTTCCATGACTTTGCATGACAACATGTTCAACTCGGTA

ACTCGCGCTACTATGAAATTCTTCAATACTAATTCATCAGGACGTATTCTGAACAGATTCTCAAAAGATATGGGCTCGATTGATGACATGTTACCAGGTGCTATGATTGATTGTCTC

CAGATTGGCTTGACGATAGTGGGTATTATAACAGTGGTGGCGCTTGTCAACTATTTGCTGTTGATACCCACGTTTGTGATTGCCATCATATTCTACTATTTAAGACGTTTCTATCTGG

CAACGTCCCGTAGTATCAAGAGGCTTGAGGGAGTCACTCGCAGTCCTGTATTTTCGCATTTGACTGCTTCCCTGCAAGGACTGTCTACTATAAGAGCGTTCAATGCTCAGGATAAA

CTAAGAGAGGAGTTCGATAATCATCAGGATTTGCATTCGTCAGCTTGGTACATGTTCGTAGCAACAAGTCGCGCCTTTGGATTCTGTTTGGACCTTTTCTGTGTTATCTACATTAGTT

TGGTGACTTTGAGCTTTCTCCTACTGGGAACTGAGACTTTTGGTGGAAACGTGGGTCTGGCTATTACACAATCAGTGGCCCTAACTGGAATGTTCCAATGGGGAATGCGACAATCG

GCCGAATTGGAAAACCAGATGACTTCTGTCGAGAGAGTGCTCGAGTATACCAACCTGGACAGCGAGCCTCCTCTCGAGTCTCCTCCAGAAAAGAAGCCACCTCCAACGTGGCCA

AGCGAAGGAAAAATCGAGTTTTCGAAAGTTTATCTGAGTTATGTGGATGAAGAGCCACCGGTGCTCAAAAACCTCAACTTTGTCATCAAAGCTGGAGATAAGGTGGGAATAGTA

GGTCGTACCGGCGCTGGCAAGTCGTCGCTAATTGCGGCACTGTTCCGTCTGACTCCAACCAGCGGTGACATCCTCATTGATGACGTCAGCACGGCTCAGCTCGGTCTGCACGAGGT

GCGTTCGAAAATATCCATCATCCCGCAGGAGCCGGTCCTGTTCTCGGGCACCATGCGCAAGAATCTGGATCCATTCGGCGAGTATCCTGACAGTGTGCTGTGGAATGCTCTCGAA

GAGGTTGACTTGAAGGATGCAGTGTCCGAATTGGCTGGTGGATTGCACGCGAAAATGTCGGAAGGTGGAACTAATTTCAGTGTCGGACAGAGACAGTTGGTATGCCTGGCCAGA

GCCATAGTTGGCAACAACAAGATACTAATCATGGATGAAGCTACTGCCAACGTTGACCCACAAACGGACGCTCTTATTCAAAAGACAATTAGAAGGAAATTTACGAATTGCACA

GTTCTGACCATCGCTCACCGACTTAACACTATTATGGACTCCGATATGGTTCTAGTTATGAGTGCTGGATCTCTTGTTGAGTACAATCATCCTTACCTTCTTTTGAAAAATTCTGACG

GAATTTTCTTCAAAATGGTTGAACAGACTGGAAAATCAACTGCCGAAGCTCTGCACAAAATTGCTGCTGACTGTTACAAAAAATACATAGATGAGGATGACGAAGACGATATCT

ATAACAGACGAGCCAGCAGTGTAACAGATCTTTAA

NlABCC3

ATGAGCTTGGAGAAAGACCATCAATCAGAAATATTCCAGCCAGTTTCCTACCTCGCCTACTATTTATTGATCCTATTGAATTTCATCTTGACGTTCTTCAAGGATAGCTTACCCAAA

GGAAAGAAGTACAATGATAGTATTGGGGAAATGTGTCCTGATGCTATTGCTGCCTATCCTTCGGTACTTGTAGCATCTTGGATCAGGCCCATTCTTAGAAAGGCCAATAGAGGAA

AGATTTATGAAGAAGATCTTTTCGAATTAAAAGATGAACTAGCAACTGAAAACATAATTGGTGAATTTGAGAAACATTATACTGATGTGCAAGAAGAGAAACAACAAGATGGTT

CCCAATGGAAGATAACTCGATCAAAACTCTTTTTGGTTTTTGCCAAGTGTTTTATTCGAACTTTTTTGTTTGGCCTTCTACTGGCGACTTTGTATCTGATCGCTTATTTCATGAATCCT

CAAGTTCTCAGACTGATATTGGTTTATTCCGATCATGAAAGAAGAATTTGGGAGAATGTGGCTTATCCAGCTATTGCATGCTGTTTAGCATTGAGCCTGACGATTTTGCAGAGTCAA

TACCTGATGAGACTTGGAATGATTTCGCTGAAAACAAGGGTCATCTTCATTTCAGAGATTTTCAAAAAGGCATTGAAGTTGTCAGTTGAATCTAGGAAGAATGAATCAACTGGTG

AAATAGTGAATCTGATGAATGTAGATGTGCACCGAATAACCGAAACAATCAAATATGTGAACCGAATCTGGGCAACTCCACTCAGGATATTTCTCGCCATGTATTCACTCTACTTT

GAAATTGGATATTCTGTGATTGCTGCGTTTGTACTCCTGCTCACATTCATTGTGATACAAATTATAATGGGATTCTATACGAAATACTATACTTCCAAGTGTATGCATATGAAAGAC

AATAGAATGAAAATTTTATCAGAAGCCCTCTCACACATGAGAGTATTGAAATTCTATGTTTGGGAGCCTAGTTTTCTGAAAATGATCAACGAACTGAGAAGTCGTGAAATATACT

ATCTCAGAAAACTATACTATATACATATAACATCCGTATTCCTATCAACCTGCACACCGTTTTTGATGACTTTGGTATCTTTCTCATATTATGTTCTTGAAGAAGGCAACATTTTGAC

GGCTGAAGTTGCGTTTGTGTCGTTGGTTCTGTTTGAAATGCTGCAATTGCCCTTGTCTAGACTCCCAAATGTGATTTTGGGTACAATACAGCTTGCAGTATCGTACAATAGGATAGC

CCGATTTTTAAATGCTGAAGAGTTGGAGGTAAAAATGGATGTGCGTCATGATGGGAAAGATGATATTGGAACAGCATCAATTATCAATGGCTACTTTGCTTGGGGAGACTCAATT

GTTTTGAAGCAAATATCGCTCAATCTGCCCAAGGGCTGTTCGAAAGCTATTTTCGGACCAACTGGTGGAGGAAAATCTTCCATTTTGGCTGCATTGGCTGGAGAAATTCTCAAAAT

GAGTGGATTTGTTTCAACAAAGGGTAAAATTGCTACAGTATCACAAGTGCCATGGATCCAAAATGAGTCTGTGCGAGATGTCATATTATTTGGTAAAGCTTTCGATAACAAGCTCT

ATGACAAAACCATCAGGGCCTGTTATCTACATGATGATCTCGAATCTTTCACTAGGGGGGATCTTACACTTATCGGTTCCAAGGGATTGAATCTGAGTTTAGGCCAGAAACAGCA

GATCTGTTTAGCAAGGGCAGTTTACAGTGAAGCTGATGTCTACCTACTCGATGACCCTGTTTCAGCTCTAGATGAGCGTTTGGCAAAACGAGTTTTCAGAAGAGTGATAAGCGACC

AAGGAATTCTAGCTGGAAAGACAAGACTGGTTGTGACTACAAATCTGGATTATTTGCATTACTTTGACTCAGTTATATTGATTGAGAATGGAAAAATTGTTGCAGAAGCTGAGTAC

AAACAATTATTGAAAACTGGGAAACTTCTATCTCTTCTGAGGAATTGGAGTGATTTGAAACTAATGAGAGATCAAAAACAATTCTGGGCAGTGAGAATAAGTGATGACGCGAAA

AGAAAAAGTTCGATACTTCTGAATGGTAAAGACTCTGCAAAAAACTACAACGTCGATACAATGGTGGTTGGCAGCTCACCTTTCTACAGACCCAGTTTGTATGCAGAACAATTCA

CTGGCACGTCGACTTCAATATTATCAGGCTCTGAAGAGAATGAATCAATTTCTGCTGGAAAGTTGAGGTGCTCAGTTTTCTGGAAATACTTGCATATAATGGGACGCTCAAACGTT

ATCAACAGTGTTGTATTTGGGATTCTTGTGGAAGTTTTCTACTTTCTGGGCACTTTTTGGTTGGCCAGTTGGACCCAGCAACACGACAACCCAAACATCAAGCAGGAGAGCATCTTC

TATGTCTCTGTGTACGCTTCATTTGGAATAGGTTTTCTTGTAAGTATGGCAATATTCTCGTACTCAATCTACATGGGCGCGTTGAAAGCCAGTATATCCTTCCATAATGAGCTTTTGA

ACTCAGTTTTCCGTTTTCCAATGTCGTTTTTCGATCAAACTCCACTGGGCCGTTTATTGAATAGATTCGGTGGCGACGTGTATACGATGGACTCACAATTGCCTTTGAATATGCAATA

CTATATTTCCACATCATTTAGAATCATGGGTGTGCTGGTTATCACTCTACAGTATTATCCAATAGTTTTTCCAGTATTCGTTACTGTCATTCTCAGCTATTTACTCCTCCAGAATTATT

TCCTGAATACGTCAAGACAATTAGCACTTTTAGAGACTAAGTCAAGAACTCCTATTTTTTCACATTTCGAAGAAGCGCTTTCGGGTGTAACAGTGATCAGAGCATACAAAGCTGAA

GAGAAATTTATTGGTGTTAACAGATCGAATATCCAAAAGAATCTATCAATAGCTTACCACACGGTCATTGCTAACAGATGGTTGGATCTGAGAATGGAGACTATAGGCCACATAT

TTCTCTTCTTCGTTATTGGATGTGTGCTATTCACACCATATTTCAACGCATCAATGATTGGCTTAGCCATTTCATATGTAATTCAGATGACAAGTAACTTAAACATTCTTGTACAGCA

TGCATCATCAATCAATTCTGATTGTGTATCTGCTGAAAGAATCAACGAATATATTGAGAGAACAAAGGAGCCAACTTGGGACTCAGCGCCCATAGATGTACCACAGGATTGGCCC

AAATATGGGGCTATCAAATTCGATAAATTTCTTGTGCAATATCAAAATAGCCGTCATATTGCACTCAATGAGATTTCATTCACTGTTTTGCCCAATGAGAAAATAAGCATAGTTGG

CCGCACAGGGTCTGGGAAGACTACACTTATGCATTCATTATTCAGAATAATTGAGGGAAAAGGCGGCTCAATACTTATAGATGGTGTGGATATATCTCAAGTTGGACTCGGACGA

CTAAGAAGCAGTTTGAACATTATACCTCAGGATCCAGTCCTTTTTTCGGGAACTTTACGCATGAATCTCGATCCTGGCAACGAATTTTCCGACAGTGAATTGTGGCAAGTGTTACA

CGAATCCCATCTCGGCCATTTGGCCACCGAGTTGAGTGATGGTCTGAATCATGTTGTAGAAGGCACCAGTAGTTTCAGTGTAGGTGAACGGCAAATGATATGTATTGCCAGAGCA

CTTCTGAAGAAGAGTAATATTCTGATTATTGATGAGGCCACTTCCCAAATCGACTGGTGGATTGAGAGCTTGATGAAGAAAGCCATCAATGAGCACTTCAGCCAGTGCACCATTTT

ATCAGTCGACCATCGTTTGAAAAATGTTCTCGATTCGGACAGAGTGCTGGTTCTATCGAAAGGAAGAGTTGTAGAATTGGATACTCCACAGAAGCTACTCGAAAATCATGATGGC

GTCTTTTACTCTCTAGCAGTTGAGGCTGGCCTAATACGTCCGGAGCAGGAATTAGATAATCTTAATCTTATCAATGATAAGACTGAGTTGAAGAAAACACCAAGTTCGGAACAAG

TTAATTCGATCGTATCAGGTGAATCTAGTCTAGTGAATGGACAAATATTGGATGAGTTGGAAGAAAATATTGAAGAAAGTGTGATCGAAATGACAAATCTAGCTTCCAATTACAG

AATAACTCCATCAACTTCAGAAAGTTCATCAACCAATGCAGAAGAACCGGAAACGAAGAATCAAGAGATCGACCTCAATTCTCAGAATATAAAAACTCAAGTCGCTAGAGAAG

TCATTTCAGAAATAATAGATAATGCAACTTCATTGAGACTGGTTGAGGAAGAAAATGCTCTCATTCACAATGACGTAGAAACTTCATCTTCCACAGTACAAGATATTCCTGAGATA

GAAATTGATGATACAAGTGAACCAAAATTGAATGAACAGAAACTTCCCTGA

NlABCC4

ATGGTTGATAAGGATGGCAGCAAATCTGTTAACTATGGTGACAATAATACACACACATATGAGACTGTACAGATGACAGACAATCATAGCAGTAGAGACTGGGATTCTGACCAA

GAATCTCCCGATGATAACGAAGCAGTATGGATGGACGTGGATTTGAAAGGAAGTGTTGACGATGGACTTGGCACAGCGGCACACACTCTTTCGTCCAGAAATTGCTATCACTATC

GAGGGGCTTATCAGAAATCGCCATTCCAATATGTTCAGAAGAAAGGTTTCTCAAGATACAAGCACTCACTCAGGACACTCATACCGGTTCGACTCAAACCGAAAGAAAAAGAAA

AATTGGATGTCGATAGCGCAGGTCTCTTGTCATTCATAACGTTCAGTTGGCTCTCGAAATACATGTACAGAGCTTACAGGAAAGGGCTGGATTTGGATGATATTCCCGAAGGATCT

CCTTATGATAGTTGTGATTATAATTGTCAAAGACTGGAATCGTATTGGAGTGCTGAAGTTACTAGGCATGGCCCAAAGGATGCATCTCTTGGCAGAGTGGTATGGAAATTCATACG

AACTCGAGTGATTTTTGCTTCAATAATATTCGGCTTCAATTTATTATCTGGATTTATTAGTGTTACAATCTGTTTACGATCTCTTATTGTATTCGCTGAAAATGAAGACGCTCCAATAT

CGGAGGGTATAATGTGGGCTGTACTATTGACTTTAAACGAAATACTTCGAACCATTTTTTATTCGACAACTTGGGCTATCAGCTACAGGACAGCAGCTCGTCTACGCTCAGCTTGC

CTTGCTTTGCTGTATAAAAAAGTTATTCGTCTAAATTCACTTGGAAGCAGTTCCATTGGCCAACTAATCAATATCTTCGCCAATGATAGCCAACGAATATTTGACATGGTCCTATTT

GGACCTATGATAATTGGTGGACCAGTAACTATGACGTTGGGAATGTTGTACATTGTATGGCTGCTTAGTCCATGGGCGCTCAGTGGAATGCTACTTTTCATCGTATTTTATCCAGTG

CAGTACCTCATGTCTAGATGCACTGGCTACTTCCGTGGTAAAACTCTGTCAGTATGCGACACAAGAATAAGATCGGTTTCTGAAATTCTTCAATGCATTAAACTGATCAAAATGTA

TGCATGGGAATCAACTTTCGCTGATAATCTCTTCGCAATACGCAAAAAAGAGATGAGACTGCTGCATTTGACAGCTTACTGCCGCAGCTTGACAATGTCTCTGGCCACCACTTTCC

CAATTGTATCGGCCATAACAACGTTCCTCTCTCATCTAGCCGCAGGAAACAACCTCACAGCTTCACAGAAAATACTTATTATGGATGAAGTGAAAACTAATATTGGTCGTTCAATA

AACAAGACGCAAGCCGTGTGCATTGCCAATGGTTCATTCTCGTATAATTCATCAGTACCTGAAGATGAACATAACAAAAACAAATCTTCAAGTAAAAAGATTAAATACAATCCTA

AAGATAAGCACAATGGGATGGAAATTCAGAGTAACAACACTGATCAAAAGGGTCAAGTGAATGTTCTAACTGGAATAAATTTCAGTGCTGCTAAGGGACATTTAGTTGGTATTTG

TGGTCATGTCGGATCTGGGAAAACATCTCTACTGCAAGCTTGCCTTGGCCAATTGAAACTGACGACTGGAAGAGTTTATAGAGAAGGCTCGTGTGCGTTCGTGAGTCAACAGGCC

TGGATTCAGAATGCAACTCTGCGAGAGAACATTCTATTTGGAGAGAAATTTGTACCATCTCGATACTGGAAAGCACTGAATGCTTGCGCTCTCCACGATGACGTGAACTCACTGGT

TGGTGGAGATGAGACGGAGATAGGAGAGAGAGGCATGAACTTGTCAGGAGGCCAGAAACAGCGAGTTGCTCTGGCTCGGGCTCTCTACGCTGACAGAGACATATATTTCCTGGA

TGATCCTTTGAGTGCAGTTGATGCCAATGTGGGAGCTCACCTCTTCAATCATTTGATCCTAGAGGCTTTGAAGGATAAAACTGTTATCCTTGTTACTCATCAGATACAGTTTTTGAAT

AGATGTGATGAAATCTACGTGCTGAAAGATGGGCAGATCGTTGAAAAGGGAACTCACGGAAAACTAATAGCCATGGATAAGGAATATGCAGCCATGGTGAAGACATGGGAACA

ATCGCACCAAGAATCTCATGAATCCAACAATAGCAAAGACGATGATGGTGATATTAATCTTGACGACTCCTTGGGAGCAGTTAAGAGTGAGTCGATGAATAATGCAAATAACGA

ATCTTCATCCTCAAAAGCAAAAACTAACGGTACATCTGTGGAGCAGCTAGGAGTACTGACGAAAGTGGAGAAAGTGGAGAAAGGTTCGATAAACGTGGACACTTACCTGAGCTA

CATGAAGGCGGCCGGCGGCTATTCGCTGTCGTTGCTGCTCATTCTGATCATGCTGCTCAACATCGGCTGCCTCACCTTCAGCTCTTGGTGGCTAGCCTTCTGGATCAAGGAAGGCA

GTGGGGGTAAGCAGATAACTGTTGGAAATGATACCATCACTAGCTCAAATATTCTAGACAACGATAACTATACCTTTTACAGAAATGTGTATGGTGGTGCAATCGGTGTCATTATG

TTGAGCAATTTGGTTCGAAGTTTCGCTTTCTCCAAGATTACTTTGACTGCTTCAAAAAATCTGCACAATCAGTTGTTTTCAAAAGTGTTGTACGGTCCAATGGTCTTCTTCGAACAGA

CTCCAGTCGGAAGAATTCAAAATTTGTTCGCCAAAGATATTGATGAAGTTGATGCAAGGATTCCAGATACGTTAGAAAGTTTACTTCAAGATGGCTGGAACCTTATTTTTGCCATT

TTGACTATTTGCTTGGTATTCCAATGGTTCATCGTACCTCTATTCATCTTATTGATCATCTATTACTCTGTGACTAGAATCTTCAGGGTGGCAATAAGGGACTTGAAAAGGTTCGAGA

ATACCACTAGATCGCCAATTTTTAGTTCAATCTCAGAAACTGTATCTGGGCTGGATACAATTCATGCATTTGAAAAAGAAGCAGAATTCACTAGAAAGTTTTCAAAAATATTTGAT

CAGAATACGACGTGTGTTTACATGTGTTGTATTGCTATGAGATGGCTTGCATTGAGGATTGATTTTTTATCAGTGATTGTCACTGGTATAACAGCAACGTTTGTGATAGTATTCCACG

GTCAAGTTGCGCCAGCCCTGGCAGGGCTTGCCATTGCCTATTCATCCACCATCAGTGGCATTTTCCAGTACACCATACGACTGTTGGCTGATGCTGAAACCAGATTCATATCCGTC

GAACGCATAATGGGATATGTTCATTCCTTGAAGAAAGAAGGTGGCTATGGTGTTACTTCGAAGCCCCCTGACGATTGGCCAGCCAAAGGATCAATCAAATTCGAAGACGTGTGCT

TACGCTATAGAACTACTCTGCCTCTCGTACTCAAGAATATATCATTCAAAATTCGCCATGGCGAAAAAATAGGTATCGTTGGCAGAACAGGCTCAGGGAAAAGTTCGCTGACTGT

GGCGTTGTTCCGCCTGGTCGAGTTGGCCAGTGGATCGATATCCATCAACAAGGTCAACATCGCTTCCGTCAACCTAGATCTCCTTCGACAGAAAATTTCCATCATACCTCAAGACC

CAGTGCTCTTCACGGGGACAGTCAGGAGTAATCTTGATCCGTATGGAAAAGCAACGGATACTGAACTGTGGCAAGTATTGGAAAAGACTCATTTGAAAGAGCGAATTTCAATGG

GATCTGGAGGATTGGACTCGGACGTGGGACAGTCTGGCGACAAACTGTCAGTTGGCGAAAGGCAACTACTGTGTCTTGCTAGAGCTCTACTAAGAAAATCAAAGATTCTAGTTCT

GGATGAGGCTACAGCAGCAGTCGATCCTGACACAGAAGCAGCCATTCAAGGCACAATCAATGGGGAATTCGATGACTGCACAGTTCTGACCATAGCACATCGCTTGGTCACTGT

CAAATCGTGCGATCGCATCATCGTGATGGACGATGGAATGGTAGCCGAAATGAACACCCCTTCAGCTCTTCTAGATGATCCGAATTCGAGATTTTCAAAAATGATTGAAGCGGCT

AAAGAGGCAGCTAAAAGTGAGAATATTTCTGTAAAGTGA

NlABCC5

ATGCGGTGGAACTGGAATTGGACAGAGTTTTGTGGCACAAGCGACGGCTTCGTGACATGGGATTCGCAGCTGGGGGATGTGGGCATGTGTTGTCAGGCCCTGTGCCTTCATATGC

CCGCTCTCTTCCTCTTGGCCATGCTCTCGTCGCACTACTGTGGCAAGCAAGCTGGCTGGGTTGTGCGCTCCACCACTGAGATCTGCGTTCTGCGCATTCGCTGTGCCATAGCCATCC

TTCTTGCATTCACGCCTATCGTTCGCAGTCTGGTTGAACTCAATGACTTCCATTTCACTCTCTACCCTGTCAACCACACACTCGTGGCTGTTGAGAGCTTCTCTTGGCTTGTACATTCC

ATGTATATTCATGCACTTCGTCACAGGTTGGGTCCTTCATTGCGAGGGCCGACAGCTATGAACGTGGTGTTGACGCTGAACTATTCTCTGGCCCTGATGACGCTGCGGTCGCAGTC

GCTGCTCCACATGTGGGCGGCTCCCATGGCTTTCGCCATTGTCCGAGTTGTACTGCAAACCACATACATCATCACCCTGATGCCTTCCTCTTCAGCCGCTGGTCCTGGTGGCTACAT

ACCATCAATCGCCAATCTTCAGGCAACGGAATCTTCACCACTGCTGCACGCGTACAGCAGGTTTCGCGAGGACATGGACCCAGGCTACTTGGGGGTGGCGATCGAAGGTTGGGG

GTGGATGTCGCAGCTGTTGCTGTCGTGGGCGGGGCCCCTCATGGAGAAGGGGGCCGTGGGTGACATCCACACGCCCGATGACCTCTACGATCTGCCCGTGTCGCTGTCCGCCTCTC

ATCTGCATGACCAACTCGAAACCTCGTTCACCGATCGATCGCTCTTCAAAGCGTTACACAGATGCTATTGGAAAGAGTTTTACGGAATTGGAATATTGAAGTTTGTGGCCGATGTT

TCTGGATTTCTCGGACCGCTTCTGCTCAATAGACTCGTGCTATTTGTTGAAAATAGGAATGAGCCTATCCACTATGGCTACTTGTATGCGGCTGGTCTTTTCATGTCATCTTTGACAG

TTGCTTTTTGCACAACCCATTTTAACTTCCTCATGGGAAAAGTTGGAATGAAGATTCGAGGATCCCTCATCACAACAGTATACAAAAAAACGTTGAAGCTCAATTCTATTTCTTTAT

CGGAATTCAGTATTGGAGAGATCGTCAACTTTATGAGTACAGATACGGATCGTATTGTTGGTGCTTGTCCAAGCTTTCATGCGTTTTGGAGTATTCCATTTCAACTAGCCGTGACTC

TCTATCTGTTATTTCGACAAGTTGGAATATCATTTCTTGCTGGCCTAATTTTCAGCATAGTTTTGATACCAATCAACAAATGCATTGCTTCTTGGATTGGAAACCTGAGCAAGAAAA

TGATGGATGAGAAAGATAAAAGGGTGAAAATGTCCGCCGAAGCTTTGCGGGGGATTAAAGTTATAAAAATGCATGTCTGGGAGGCATATTTTGTCGAAAAAATAATGGGAATCA

GAAATAAAGAAATGAAATACTTGAAAGGGCGTAAATACCTGGATGCGATGTGCGTTTACTTTTGGGCCACAACTCCAGTTCTTATTGCTCTTCTCACGTTCACTGTCTACACGTTGC

TTGGACACAAGTTGAATGCAGCAACTGTATTCACCAGTATTGCTCTTTTCAACATGCTCATCAGTCCATTGAATGCATTCCCATGGGTTTTGAATGGCCTTACAGAAGCTTGGGTTT

CATTACAACGAATTCAGAAGCTTGTGGAGAAAAAAGAACAAGAACTGAGTGAGTACTACAGTGATATGCCTACTGTTGAGTCAGTGAGTGAAGGAGCGTTAGCAGTTCATGGTG

GTCATTTTCATTGGGGAACTGGAAATTTCCAGCTATCTTCCATCAACCTGGCTGCTATCAAGGGTCAACTGATAGGAATAACAGGACGAGTTGGTTGTGGCAAGTCATCATTTTTG

ATGGCACTTCTGGCTGAAATGGAGAAAACAGTTGGATTTGTGGCTGTTCCTGATGCTGAACATGGATTTGGCTTTGTTAGTCAAACACCATGGCTTCAGCACGGCACCATCAGGGA

CAACATTCTCTTTGGCAGGCCATTCGATCACAACCGTTATAAGAAAGTTTTGAATGCATGTGCATTGAATGAAGATTTGCAGATATTGCCTGGAGGCGATCTAGCCAATGTAGGAG

AGAGGGGTGCATCACTCAGTGGAGGACAGAAGGCTAGAGTTTCATTGGCAAGAGCTGTCTATCATGACCGTAACGTGTACCTTTTAGACGACATTTTCTCAGCTGTTGATCCACGG

GTTGGGAGGCACATTTTCCACTTTTGTATTAATGGGCTTTTAAAAGAGAAAACGAGACTGCTCTGTACCCATCACACTCAATTCTTGCTTGGAGCTGACCAGGTTCTAGTTCTTGAG

GAAGGCTCTATAATCAAACAAGGTCGCCCTCAAGACGTACTACCTGACTACGAGGAGGACATGACCCAGAGCCAGCTGATGGAGACGGAAGTGGCGGGCTCCCCTACTGCGTCG

ATCGCCGGTGCCGCCTCCATCGCCTCCACGGAGCGAGCTCCACCAGTCGAGTCGGGGGTAGAGGCGGAGATGGAGGCGGAGACTGAAGAGTGGAGTGCCCGTGGCTCCGTGTCA

TGGAGTGTCTATTCGCGCTACTGGTCGGCCATCGGCTATGGACTGTCGGTTGCCATTCTTGCGTCCATCTTCTTCATGCAGGCGTCTCGTAACGCCACTGATTGGTGGCTAGCGCTGT

GGGTCACCAATGCTCGTGGCTGGAATAGTACTGTCGGCTTGACTTTGGACTGGGAAGATAGCGACTCAGACTCTCGCAGGTATCTGACCGTTTATGCGTGCATAGCTGGGGTCAAC

TCACTTCTAACTCTGTTCAGAGCTTTTCTATTTGCTCTGGGTGGCATAACAGCTGCTTCAAAACTGCACAATGCATTGCTCAATACTATTATATCCGCTCAGAGTACATTTTTCGATG

TCACTCCAGCAGGAAGGATATTGAATAGATTGTCATCGGACACTTATACAATAGATGACTCCTTACCTTTCATTGCCAACATATTCCTCGCACAGCTATTCGGTCTCATAGGAACA

GTGATAGTGACAGTCTATGGATTGCCTTGGATCACGCTACTCCTACTGCCTCTCATTCCAATCTACCTGTGGATTCAGAATCATTACCGGCTGACGTCCCGAGAACTGAAACGACTT

TCTACCACATCACTGTCGCCGCTATACTCCCATTTCAACGAGTCCCTCGAGGGTCTAGTCACTATCAGAGCTTTCAGAGCCTCTTCTAGATTTTGGAGACATAATGAGCTCTGTCTT

GAAAGCAACCAGAAAGTGCAGCTAGCCAGTCTTATTGCTAATCAGTGGTTAAACCTTCGCCTGCAATTGATAGGAGTTGCAATGGTTACCAGTGTCAGTGTACTCTCCATCTTCCA

ACATGCCTACAATATTGCTAATCCAGGTCTGCTAGGTCTAGCACTGTCGTACGTGCTGGGCGTGACGGGTCAGCTGGGCGGAGTGGTCTACGCGTTTGCCGAGACAGAGCGTGAA

ATGGTGAGTGTGGAGCGAGTGGACCAGGCTCTACGCCAAATCCGACCCGAGCGATTCGCCAGCAAGGTGGACCAGGCTCTACGCCAAATCCGACCCGAGCGATTCGCCAGCAA

GGTGGCGCCGCCATATGCCTGGCCCAGTCAGGGCGTCATCATATTCAACAACGTCTACCTCAGATATCGCCAATGTCTTCAACCGTCTCTGAGGGGTATTTCATTCTCAACTCGCC

CTGCCGAACGTATCGGCATAGTTGGCCGAACAGGTGCCGGCAAAAGCAGTCTACTGGCTGCTCTCTTTCGTCTGGTAGAACTCAGCTCAGGCCAGATTATTATCGATACAGTCGAC

ATAGCTATGATTGGTCTACAACAGCTCAGGTCTCGTTTGGCGGTGATCCCACAGGAGGTGTTTGTGTGGGGCAGCAGCCTCCGCGAGAACGTGGATCCGGGAAGTGTGTGGGGCG

ACGCAGACGTGTGGCGAGCCCTGGAGCGATGTCATCTCGCCGCAGTCGTTCGCGCCCAGGGCGGCCTCCACATCCCACTCCAGCCCAACAAGACCCTCTCCAACGGCCAGGCTC

AACTGTTGTGTCTTGCGAGGGCGCTCCTCAAGAATGCCAAGATCCTATGCATTGATGAGGCGACAGCGAGTGTTGACGAGGAAACTGATAGAAAGATTCAGGAAACAATAAGGA

CATCATTTCGACAGTGCACTGTTTTGACAATTGCCCATCGCATCTCCACCATTATGAATAGTGACAGGGTTCTCGTGATGGGCGATGGAGAAGTTCTGGAATTTGACACTCCTGAA

ATTCTTCTAGAAACAAAAACGTCTCACTTCTGTAAACTTGTTCACAAAGACTTTGAATAA

NlABCD1

ATGCCTACTGTGATATCAAAATATTTGGACGGAACTACCCGTAAATATAATATTCCTAAAGAATATGTTACCAGAGGTATTGTTTCGGCTGCAGTTCTGATTTATGGTTGCAAGTTG

AGCTACCCCTATTTGAAAAGTATTTGTGGTGAACGGACTCCCAAATCCAAGAAGAAGTTACTGAGCAATGGTGCTAATAATGGTAAACTTGTGAATGGTTTTGGGAATGGTGTATC

AGATTCTGAAGATGAAAAATCTACGAACAACAATAAAGTTGCAATCCCCAACGGTAATGCTCTTAAAAAACACACAAAAGATGCGAAAAGTGTTAAAAGGAAAGGAACAAAA

ACGCCAGGTTTTAATATGGAGTTTGTTTATCAACTGATAAAACTGTTGCAGCTTATGGTTCCTAGTGTTTTTTGTCATGAAACAGGAATTCTAGCAGTGCATACTCTGGCTCTCTTTA

CTAGAACATTTATGTCGATTTATGTTGCAAGTATGGAAGGCCAAATGGTCAAGTTTATAGTGCAGCGAGATGTTAGAAATTTTGCATTTATGTTACTGAAGTGGCTTGGAGTTGCTC

TTCCGGCTACCTTTCTCAACTCAACAATTCGATTCTTGGAAAATAAACTGGCTTTGTCATTCAGGTCGCGACTGGTGGACCACGCATACGGCCTGTATTTCAGCGCGCAGACGTATT

ACCGGGTGAGCAACCTGGACGGGCGCATCGAGAACGCAGACCACCGCCTGACCGAGGACATAAGCGCGTTCGCCTCGTCGGTGGCGCACCTCTACTCGCACCTCACCAAGCCAC

TCTTCGACTGCGCGCTCATCGGATTCGCGCTGGCCCGATCCAGTCGGCAGATGGGCGCTGCTGTTGTCCCAGGTCCCCTGCTAGCGGTGATAGTGATTTCGCTGACGGCGCAAATT

CTGCGAGTGCTGTCGCCGAAGTTCGGTGCCCTGGTGGCCGTGGAGGCAGACCGGAAGGCTTATCTGCGTCACATTCACAACCGGGTCATCACCAACGCGGAGGAAATCGCTTTCT

ACGGCGGACACAAGGTGGAGCTATCGCATTTACAGAAGGCCTACCAGGCATTGGTAGCGCACAAGAACAAGATATTTGTGCAGCGACTGTGGTATGTGGTGCTGGAACAGTTCC

TCATGAAGTACGTGTGGAGCGGCACAGGCATGGTCATGGTGTCACTTCCTATTATCATGGCTACACGAGCTGTGAATGGTGAGAGCGACATAACCGGTGACAAGATCAGTGAGA

GAACGCAGTACTTCACAACTGCCAGGAATTTACTTCTGTCGGGAGCTGATGCTGTTGAAAGGCTTATGTCTTCTTACAAGGAAGTGGTTGAGTTGGCAGGCTACACGTCACGAGTG

GGGGAGATGCTAGATGTGTTCAGTGAGGTGAGCTCGGGCAAGTACACGCGCACCATAGTCGCCTCCAAGCAGCATCCCAGTCTGCACTACGCAGACGGCCGTCTTCTAGTCAAA

GGTGTGATAAGCGACAGCGTGGACGGCAGCATCAGTGTGCGAGACATGCCGATCGTGACTCCGAACGGAGACGTGGTGGTGCCGAGCCTCACACTGAGCATGTCACCCGGTGAC

CACCTGCTCATCACAGGCCCCAACGGCTGCGGCAAGTCCTCCCTCTTCCGCGTGCTCAGTGGCCTCTGGCCCGTCTATGCCGGCCACCTCATCCGCCCGCCCAACTGCTCCATGTTC

TACATTCCGCAGAGGCCATACATGACGCTAGGCTCACTGAGGGAGCAGGTGATCTACCCGGACACAGTAGAGGAGTGCCGGCAGAAAGGAATCAGCGACTGCCAGCTGGAGCA

GTGCCTGGAGAGGGTGTCGCTCGCCCACCTGGTGGCCAGGGAGGGGGGATGGGACGCCACCGCCGACTGGAAGGATGTCCTCTCCGGTGGCGAGAAGCAGAGGATGGCATGTC

GCCAGGCTATTCTATCACAAACCGGCGTTTGCATTACTGGACGAGTGTACAAGCGCAGTGAGCATTGA

NlABCD2

ATGGCTCCATCGCTCAGTAAATATGTAACGAAAAAAACTGTTTTTGGTGCCGCTAGTGTATCGGTTATCCTATGGCTGTTAACACGGAGGAAAAATCAAAGTAAATCCAAAGCCA

GGAGGCCTCCCCATATGGATATTCAGTATATGATTGGAGACAAATCGCAATCGAAGACAAAAGCTCAAGTGGACGCCATTTTTTTCAAGCAGATCCGTCACATTCTGAGCATCGC

GATAAAGGGCGTGCTCACACCTGAAGCCGGCTTTCTGCTGCTAGTGGCTGGCTCCCTGGTGGCCCGATCGCTATGCGACATATGGATGATACAGAATCACACATCTACCGAAAAT

GCAATCGTAACAATGGATAAGAGTCGATTCAGAAAACATCTACTGGAGTTTTTAATAGCTATGCCTGTGATATCTTTGGTTAATAATGTATTGAAGTGGGGAATTGGAGAAATTAA

ACTTAGATTAAGGACTAGGATAACGCATTATCTGTACGATGAGTACTTAAAGGGCTTCACCTACTACAAAATGTCGAACCTGGACACGCGGATAGCGAACGCCGATCAGCTGTTG

ACGACGGACGTTGATCGATTCTGCGACTCTTTCGCCGACCTCTACTCCAACATCTGCAAACCTCTGCTCGACATATTCATCTATGTCTATCAACTGACTACAAATGTGGGCGGGGC

CACTCCTGGACTTATGATGGTCTACCTTGTCTTCGCCGGCATGTTCCTCACTTATTTGCGACGACCAGCCGGTAGGATGACAGTACAAGAGCAGAAACTAGAAGGAGAGTTTAGGT

ACATAAACTCTCGACTCATTGCCAACTCAGAGGAGGTGGCCTTCTATATGGGAAACAATCGAGAGAAACTCACACTGTTGGCTGCTTTCCAGAAACTGACAGACCACATGAGAC

ATTTCTTGGAGTTCAGGGTGCTAATGGGTACAGTTGATAATTTGGTTGCCAAGTATTTGGCAACAATTGTTGGTTTCTATGCGGTTAGTATGCCTTTCATGGATGCCTCTCACCTTGT

GCTAGCTCATGCTTCGAAGGATGACAGGTTCAAGCAATACTACGCGTCAGGTCGTATGCTTGTGAAACTGGCGGAAGCAATCGGTCGACTGGTGTTGGCCGGCAGGGAGATGACC

CGACTGGCTGGGTTCACGGCAAGAGTCACCGAAATCATCACCGTCCTTAAGGACATCAACCAAGGACACTACAAGCGCACCATGGTTGACAGCAAAGGATCCACCAATCCTGCC

ATCGAGAACGGCAAGGAGAATGGAGTTGCTGTTTCGAACGGCATAACAACACCTCTCATTCCGAACTCTGGCAAGATCATATTCCAGGACAACATCATCAGATTCAACAAGGTG

CCTCTGGTCACACCAAATGGAGACTTACTCGTCAAGGAGCTCTCATTTGAAGTCTGTTCACTTTTTATTCCGTTTTAA

NlABCD3

ATGTCTGCAAGTAATTCAAGGAGCAAGGAAATGAAATCTGCAGCATGTTTGCCATCTATATCACCTATCAGACAAACAAGCAATCTAAAGTGCAAATCTGAAGCTAATAGTAAA

TCAAACAAGAGAAGTTGTGAATCTACATGTAAAGGACATATAGTGTCATTCAGAAATGTCAGTCTTCAATACAGAGGATTACCAAAAATTGTAAGGAATCTAACATTTGAAATTG

ACCGGTTCGAAAAAAATTGGGGTAGTCGGCTCAAGAGATTCAGGAAAAACTGCACTTGTTCTTGCATTGTACAGGCTAACTGAGCTTACAGAGGGATGTATAAAAATTGATGGAGT

TGATATATCAAAAGTAGACAACCATGTGATTCAAAAGAGAATAAAGATTATTCATCAAGATCCAATTATCCTTTCTGGCAGTTTGAGAGCTAATCTAGATCCGTTCAAAGAATTTG

AAGACACCCATTTAATCAATGCACTGGAAAGAGTTGGTTTGAGTGATAAGTTGATAAATGGAAACTCAACTAATCTAGATGCAGAAATACAAACAAACTGCCTTTCTATTCCCGA

AAAGCAGCTTATCCTGATTGCAAGCTCCTTTCTTCACAAGCCAAAGATCTTAATAGTTCAAGAAGCTCAAGAATCGTGGGCAGGAGGAGATGCCGTTAAATTAAGCAAGTCTCTT

GAAGTGATTGAGACTGTTTGCAGAGAGTTTCCAACCTGCACAATTCTTCTATTTATAAATCAACCACAAGCCCTGAAATTATGCAACAAAGTATTGTTCATCGAAAAAGGGAAGG

TTGCAGAGCAAGGCGAACCTCACTCACTTATGAAAACTCAGTCTTCGAAACTGTACCAGTTCATGGCAAAAAAAATTCAATACCAAAGTCCAATGA

NlABCE1

ATGCCTCGCAGTAAAGCAACAGAGGAAACTGACAAATTGACTAGGATTGCCATTGTTAATTCTGACAAATGCAAGCCTAAAAGATGTCGTCAGGAATGCAAAAAGTCCTGTCCT

GTTGTGCGTATGGGCAAACTGTGTATAGAAGTGTCACCAAATGACAAACTCGCATCTATATCCGAAGAACTCTGCATTGGTTGTGGTATTTGTGTAAAGAAATGTCCATTCGAGGC

GATCACAATTATAAACTTGCCGAGCAACTTGGAGAAGGACACGACGCACCGTTACTCCCAGAACTCATTCAAGTTGCACCGGCTGCCGATTCCCAGACCTGGTGAAGTTCTGGGA

TTGGTCGGCACCAACGGAATTGGCAAGTCAACCGCACTCAAAATCCTGGCCGGCAAGCAGAAACCCAATCTGGGACAGTTCACGAACCCACCCGATTGGACGGTAATTCTTAAC

CACTTTCGAGGTTCGGAACTGCAAAACTATTTCACAAAAATCCTGGAGGACGATTTGAAAGCTTTAATAAAGCCGCAGTACGTGGATCAGATACCAAAAGCCGTCAAGGGTACC

GTTCAGCAACTACTAGACAAGAAGAACGAGAGGGACAATCAAAATCTTATCTGTGATTTATTAGATTTACAAAAGATCAGGAACCGGTCGATAGAGGACTTGTCTGGTGGTGAA

CTGCAGCGGTTTGCGTGTGCCATGGTGTGCATCCAAGATGGCGACATATTCATGTTCGATGAGCCGTCCAGCTATTTGGACGTGAAGCAGCGTCTCAAAGCCGCCTCCACTATCCG

ATCCCTCATACAGCCTGATAAGTTCATAATAGTGGTGGAACACGATCTGTCGGTGCTTGACTATTTGTCTGATTTCATCTGCTGCCTGTACGGTGTGCCTGGAGCATATGGAGTGGT

CACTATGCCTTTCTCGGTCAGGGAAGGTATCAACATATTCTTGGACGGATTCGTTCCGACGGAGAACCTTCGTTTTCGCGATGAGTCACTTGTTTTCAAAGTGGCCGAATCTGCCAC

TGAGGAAGAAGTGAAGCGTATGAACCACTACGAGTATCCGGCCATGACTAAAACCATGGGCGACTTCCATCTCGAGGTACGACAGGGTCAGTTCACCGACAGTGAAATCCTGGT

GTTGCTGGGCGAGAACGGCACGGGCAAGACGACATTCATCCGACTGCTGGCCGGCAGAGACCGACCCGACAGCGGCTCGGCAGACGTGCCCGTGCTCAACATCAGCTACAAGC

CACAGAAGATCAGTCCCAAGTACAATGGCCAGGTCCGCTCCCTCCTGCATGACAAGATCAAGGACGCCTACATACATCCACAGTTTGTGACAGACGTGATGAAGCCGATGAAGC

TGGACGACATAATCGACCAGGAGGTGCAGAATCTGTCGGGCGGGGAGCTGCAGCGTGTCGCGATGGCACTCTGCCTCGGCAAGCCGGCTGACGTCTACCTCATCGACGAACCAT

CCGCCTACCTCGACTCCGAACAGCGCTTGGCCTGTGCCAAGGTCATCAAGAGATTCATCCTGCACGCAAAGAAGACAGGCTTCGTGGTGGAGCACGACTTCATAATGGCGACGTA

CCTGGCCGACAGGGTGATCGTGTTCGAGGGCATCGCGTCGGTGAGCACGACGGCGCACTCGCCTCAGTCGCTGCTCGCCGGCATGAACCGCTTCCTCGAACTGCTCGGCATCACT

TTCCGTCGCGACCCCAACAACTTCCGGCCTCGCATCAACAAGGAGAACTCGGTCAAGGATACCGATCAGAAACGAGCTGGACAGTACTTCTTCCTCGAGGATTAA

NlABCF1

ATGACGAAGAAAGGTGGTCAAGGTCATAGTGACCTAGGAGAGAACTTCACAGTTTCGCAAACACAGAAGTCTGTAGGACAGCTTCAAGCTTTAGAAAATGCCGTCGATATAAAG

GTTGAGAACTTCAGTATATCAGCCAAAGGCAACGATTTGTTTGTCAATGCCAATCTGCTGATTGCTCACGGTCGAAGATATGGTCTCGTTGGACCCAATGGTCATGGCAAAACAAC

CCTACTGCGTCACATTGCTTCGCGCGCTTTTGCCATTCCGCCGGGCATTGACATCCTCTACTGCGAACAGGAAGTGGTGGCCGATGATCTGTCGGCAGTCGATTCTGTGCTCAAAGC

TGATGTGAAACGAACCAAACTGTTGGATGAGTGCAAAGACCTCGAAGCCAAACAGGAGAAAGGAGACATGACTGTACAGGAACGTTTGAAAGAGGTGTACGAAGAGTTGAAA

GCCATCGGAGCCGACTCAGCCGAGCCGAGGGCCAGACGAATCCTGGCCGGTCTGGGTTTCTCTAAGCCGATGCAGGACAGAGCCACTAAGAACTTCTCGGGAGGTTGGCGTATG

AGGGTGTCACTGGCCAGGGCCCTTTTCGTCGAACCCACTTTGCTTCTGCTCGACGAACCTACCAACCATCTCGACCTCAACGCTGTCATTTGGCTTGATAATTACCTGCAAGGCTGG

AAAAAGACTCTACTCATAGTTTCTCACGACCAAAGTTTCTTAGACAACGTCTGCAATGAAATAATCCATCTCGACCAGCAGAAACTCTACTACTACAAAGGCAACTACTCTATGTT

CAAGAAGATGTACGTGCAGAAGCGCAAAGAGATGATCAAAGACTACGAGAAACAGGAGAAGAAATTGAAAGAACTGAAGGCCCACGGGCAATCTAAGAAAGCCGCCGAGAA

GAAACAAAAGGAAGCTCTGACCAGGAAACAGGAGAAGAATAAAACGAAACAACAGAAAACAAATGATGACGAGACGGGTCCGATTGAACTTCTGCAGAAACCCAAGGAGTA

TATTGTGAAGTTTTCGTTTCCAGACCCTCCTCCGCTCCAACCGCCCATTTTGGGACTGTATAGTGTGCATTTTGGATATCCGGGGCAACCGAAGTTGTTCACAAATGTTGATTTTGGA

ATAGATATGAACTCTCGTGTCGCCATTGTGGGTCCCAATGGTGTTGGAAAATCGACGTTTCTCAAGCTGCTTACTCTCGATTTACAACCAACAAAGGGCGAGGCTGCCAAAAACC

ATCGATTGCGTATCGGCCGTTTCGACCAGCATTCAGGCGAACACTTGACGGCGGAAGAGACCCCGTCCGAATACCTGATGCGTCTATTCGACCTGCCCTACGAGAAAGCCAGGAA

ACAGCTGGGTACTTTCGGTCTAGTTTCGCATGCGCACACAATCAAGATGAAGGACTTGTCTGGAGGTCAGAAGGCTCGTGTGGCGTTGGCCGAATTGTGCCTAAATGCTCCCGATG

TCCTTATACTCGACGAGCCTACCAACAATCTCGATATTGAGTCGATTGATGCGCTAGCTGAGGCTATCAACGAGTATAAAGGAGGTGTCATAATAGTTTCTCACGACGAGCGACTT

ATCCGAGACACCGACTGTACTCTGTGGGTCATTGAGGATCAGACAATCAATGAAGTTGATGGCGATTTCGATGATTACAGAAAAGAACTTCTAGAGAGTTTAGGGGAAGTGATCA

ATAGTCCAAGTATAGCAGCCAACGCGGCAGTTCAGCAGTAA

NlABCF2

ATGCCGTCTGATGCAAAGAAAAAACAAGCAGCAAAAAAGAAAGAGGCAGCAAAAGCCCGACAAGCAGGACCAGCTAAGAAAACCACGAAAGTGGAGAATGGAGAAACCAA

TGGCGAGAATGGAATTGAAATAAGCGCTGAAGAGCAATTATGTCAGAAATTGGAGGCTGATGCTAAACTAAATGCGGAGGCCAGATCCTGCACAGGAAGTTTGGCTGTCCATCC

ACGCTCTCGAGATATTAAAATTGATAATTTTTCAATAACATTTCATGGCTGTGAGCTGTTGCAAGACTCCATGCTTGAGTTGAATTGTGGACGTCGCTACGGTCTGCTAGGTCTCAA

TGGAAGTGGAAAGTCAACTTTATTGTCTGTTCTTGGTAACAGAGAAGTTCCTATTCAAGACCACATAGATATATTTCATTTAACTAGGGAAATGCCCGCTTCCGATAAAAGTGCTC

TTCAATGTGTGATGGAAGTGGACCAGGAAAGAGTCCGCTTAGAAAGAATGGCCGAGGAATTGATTGCTTGCGAAGATGATGATGCTCAAGAACAGCTAATGGATGTTTACGAGA

GGTTGGATGATATAAGTGCAGATACTGCTGAATCTAGAGCCGCAAATATTCTCAATGGTCTTGGCTTCACTAAAGAAATGCAGCAGAAGAAGACCAAAGACTTTTCAGGTGGTTG

GAGAATGAGAATTGCCCTTGCCAGAGCCTTGTATGTGAAGCCGCATCTTCTGCTGTTGGATGAACCAACCAATCACTTGGATTTGGACGCTTGTGTGTGGCTGGAAGAAGAACTCA

AAACGTATAAAAGAATATTAGTGATTATATCACATTCGCAAGATTTCCTAAATGGAATCTGTACAAATATCATTCATCTGAACAAGAAGCGACTCAAGTATTACACGGGTAATTA

CGATGCGTTTGTGAGAACCCGGCTTGAGCTGTTGGAGAATCAGATGAAGCAATACAATTGGGAACAAGATCAAATCGCACACATGAAGAACTACATTGCCAGGTTTGGTCACGG

AAGTGCCAAGTTGGCGCGCCAGGCTCAAAGTAAGGAGAAAACACTCGCGAAAATGGTCGCTCAGGGTTTAACCGAAAAAGTCACCAACGACAAGATTGTAACCTTCTATTTCCC

TTCTTGCGGCACAATCCCCCCTCCGGTCATCATGGTCCAGAATGTCAGCTTCAGATATAACGAGAGCACACCTTATATTTACAAAAATCTCGAATTTGGAATTGATCTGGACACTC

GGCTGGCTCTTGTGGGACCCAATGGTGCTGGAAAAAGTACCCTGCTGAAACTGCTGTACGGTGATTTGATCCCAACCGAAGGAATGATTAGGAAGAACTCTCATCTACGAATTGC

CCGTTATCACCAGCATTTGCACGAATTGCTGGATCTGGATATTTCTCCCTTGGATTACATGTTGAAGTCTTTCCCCGAGGTCAAAGAAAGGGAAGAAATGCGAAAAATCATCGGAC

GTTACGGTCTCACTGGTCGGCAGCAGGTGTGTCCGATCAGGCAGCTGAGTGACGGACAGCGATGCCGGGTGGTGTTCGCGTGGCTGGCGTGGCAGGCGCCGCATCTGCTGCTGCT

TGACGAACCCACCAACCATCTCGACATGGAGACCATCGACGCCCTCGCCGAAGCCATCAGCGACTTCGAAGGCGGCATGGTCCTCGTCAGTCACGACTTCCGTCTCATTAGCCAG

GTTGCTGAAGAGATTTGGGTGTGCGAGAATGGAACGGTGACAAAGTGGCAAGGAGGCATCAAGAACTACAAGGAGCATCTGCGATCCAAGATTCTGAAAGATAGTCAGGCTAG

AGATGGACCTCAGAAAAAGAAATGA

NlABCG1

ATGGATTATCCACCCGCAGTGGCCAACATAAACCATCTAATCCAGAGGCCACCGGTTGATATAGAGTTCACAGATCTCACCTATACAGTTCCTCATGGGAGAAGTGGTTCAAAGA

TTATCCTTAGAAGTGTGAGTGGTTTATTCAAGTCGGGACAATTGACTGCTATCCTGGGACCCTCAGGAGCCGGGAAAAGCACCCTTCTAAATGTACTAGCAGGATATAAGTGTGC

AGATTCTACCGGCTCCATCCTAGTCAATGGCCGCCCTCGAGCTCTGCAACAGTTTCGCAAATTGTCACGCTACATAATGCAGGAGGACATGCTTCAGCCCCGCCTAACAGTGCAG

GAATCCATGCTGTTTGCTGTCGACTTGAAGCTTGGAACCACCATTTCACGGGAGGAAAAGCTGGATACTATTGACGAGATTCTGAACATGTTGAGGCTTTCAAAAACGAAGAACA

CTCTGTCTGGTCACTTGTCTGGTGGAGAGAAGAAGAGACTGTCCATTGCTCTCGAACTGGTGAACAACCCACCTGTTATCTTCCTTGATGAACCAACAACGGGCCTAGACGACCTG

GCCAGCTCTCAGTGCATATCCCTTCTGAAGGCATTGGCGAGGGGCGGTCGCACAGTCATCTGCTCCATTCACACGCCGAGCGCCCGCCTCTTCTCACTGTTCGACCATGTCTACAT

CGTGTCGGAGGGCCAGTGCGTCTTCCAGGGCCATGGCCACGACATTGTCGCCTTCCTCGCCTCGTTCGGCCTCAACTGTCCCAAGCACTACAATCCCGCCGATTTTATGGTTGAAG

TATCTAGTGGAGAGTATGGTGATTATCTGGAGAGAATGACTAATGCCATTGAGAATGGGAGGTGCTATAAGTGGAATCAGAACAAAGTCACTGACGTCCGATACCAAGCAAATG

AAGAGGAAGAGAATCTAGTCAGTACTGATCTCCATCACATGTACAATTTCGAAAGTTCAGCGTGGTTACAGTTTAGGATATTAATAAGTAGAATGTCTCTACAAGGTAGAAGAGA

TATGGGATATATTATATTGAAATTAGCAATGCATATTTTCATAGGAATGATTATTGGAGGGATGTTTTTCCAAATCGGCAATGATGGCTCGAAGACTATTTTCAATTTTGGTTTCTGT

TTTGTTACGATTATCATTTTCTTGTATATACCAATGATGCCAGCGCTTCTATGGTTTCCTCAAGAAGTGCAGTTATTGAAAAGAGAATTTTTCAATAGATGGTATGATCTGAACCCGT

ATTTTTTCGCCATGACATTCTGTCAGTTGCCGTTGCAAATTGTTTTTGGAATCGGGTATGCACTGTTAACGTACTTCATGACAGATCAGCCAATGGAGTATGAGAGAGTTCTCAAAT

TCATTCTGGTGTGCCTGATGATCTCCATTGTGTCAGAAGCCATGGGCTTGGCAATATCTGCTCGTCTCAATATTGTCAACGGCATTTTCGTGGGACCAGCCGTTTCAGTTCCTTTGAT

GTTACTGGCCGTGTACGGCCTAGGCACGGGGAGCAAATACATCCCTTCGCACATCCGTTTCGCCATGTACTTCAGCTACCTGCGCTACGGACTGGAAGGCCTCATATCGTCCATCT

ACGGCGGCGGACGCACCAAAATGGTCTGTCCCGACTCCGAGATCTACTGCCAACTGCGCGAACCTAAAGCGCTGCTCAAGGAGGTTGGCATGGAGGACGTCAACTATTGGTTAG

ACATTGCTGCGTTGGCAGTTTCTTTCCTGGTCTTCAAGGTCATATGCTATGTGCTCTTGAGGAGGCGTTTGAAGTCCACGCAATCGTTTGGCGCACTCGGGTTTATCGGCAGATTTAT

CAAGACCCATTTCAATCTAGCTGGAAATATAGGAAGGTAA

NlABCG2

ATGACAGCGTCCACATCATCAGCATTGGAGAATATTGTTGAAGTTTTGCCATGTCCAGGAGAGGAGCCGTTAGCGGGGTACAGCGGCGAAAGGGTCACCAAGGGGGCACTAGCG

ACTGCCAGCCGTCATCCACCCGTACCCGTCACAAGGATTCCAACCCGGCTCGGACTGACCACGCTCACCAGAATGGCCAAGCGACCCGCTGTCGACATCGAGTTCCAGGACCTCT

CTTACACAGCTGGAAGTCGGAAGATTTTGAAATCGATATCAGGATGCTTCAAGTCAGGAGAGATGACCGCTATCATGGGTCCTTCGGGAGCTGGAAAGAGTACCTTGATGAATAT

CCTTGTCGGTTATGTAACAAATGGAGTGTCGGGAAGTATTCTGACGAACGGATTTCCACGACAAATCAAGCTGTTTAATAAGCTGTCTTCGTACATAATGCAAGAGGATCTACTAC

AGCCAAACCTCACAGTCAGGGAATCCATGATGATCGCAGCTAGACTCAAACTCGGAAACGAATTGTCTGACAGAGATAAGAATGCTGCAGTGCGAGAAATCCTTGTGACGTTGG

GCTTGACTAAGTGTGCGGATACATTCACAGACAGACTGTCGGGTGGCCAGAGAAAGAGGGTGTCCGTGGGACTCGAACTCGTCAATAACCCGCCAGTTATATTTTTAGATGAACC

AACCACGGGTCTAGACATAGTAGCCATCAATAACTGCATAGAACTGTTGAAGGAGCTGTCAAGCCAGGGCAGAACAATCGTTTGCACCATTCACCAGCCGACTGCTTCCATGTTC

AACATGTTCGACAATGTGTACATGCTTGCCAAGGGCCAATGCATCTACCATGGCACTTCACACCAACTGGTGCCTTTTCTTAGTAATTGCAACCTCGACTGTCCTGCCACCTACAAT

CCAACTGATTTCGTTTTCGAAGTTTTGGAATCAAATGCAGAATTTATCAAAGTAATGAACTCTGAGATACAAAATGGTAGAGTCATCTGGTTGGATCCAACCGATAATCCTGAGCC

AAAGTCAAAATTGTGTAGAAAAGATACACTAGCTGTTATACCCAATGTTATTGGCTCGGATAGTGCAATACACTTCCCGACAACATTTTTCGAACAAGTAACGATATTGCTGAGA

AGAATGATGAAACAGAAATGGAGGAATTCGACTGCCATGAGGTTGCAGATGATCCATCATTTGTTTTCTGGACTGATTGTTGGTTCCATATTCTATGGAATTGGAAATAACGCCAG

CAAGCCGTTTGAAAACTTCAAATTCGTTCTTTGTGTTGCTGTTTTCTTCATGTACACACATGTTATAACACATATCTTGACTTTGCCAAATGAAATCAAAATCATGAAAAGAGAGTA

TTTCAACCGATGGTATGGACTCAAAGCCTATTTCACGGCGCTCACCCTTCATACAGTTCCTACTACCATAATTTTGGGTATGATATTCAACACAATCGTATACATAATGGCGGATGA

ACCATTAGAGCTGCCCAGGTTTATATGGTTCAGTTCATTCACAATCATGGTGGCTCTTGTCTCAGAAGGACTCGGAGTGCTCATCGGATGTAACTTCAACTGCACTAACGGAGCCG

TAGTGGGTCCATCAGTAATGGCACCAATACTTATGATCGCCATTCACGGAATGGGGTACGGATTACACATAAAGCCGTTCATGAAGAGTCTGATGAACATGAGCTTCATTCGCAT

AGCAGTGGTGGGCATAGTGACGAGTCTCTATCAGAATGGGCGTGGCCCGATGGAGTGCAAAGCCCAGGTGCATCCCTACTGCCACTACCGCGACCCCTACATGCTGGTCAGGGA

TCTCGGCATGACCAATCAGAGCTCGGTCAATCAGATTATCGGCCTAGTCGGCTTTCTACTCTTGTTCAGGATGGCCGCCTTTTTGACTCTCAGGTACACACTCATGACTGACATTAG

AAGTCAAGTGTTCGCCTATACTAAGAAAATATTCAAACGCAACAAGGAGAAGCGTATGTTGTTGTCTGATGAGAAGTAG

NlABCG3

ATGACTCATTTACTGGGCATCCAATGCAAAATAATGTCTGGCATCTTCAAGCTATTCAAACTCCAGAATGGGAGCGTCGAAAAAGATATGTCACATAATATCTACACAGTGGACC

TGACCCAGAGTGAGACAGGCATCGTTGCACCAGCCTTCTCGCTCAAACATCTTCCAAAGAGACAGCCTGTTGACATCAAATTCTCTGACTTGAATTACACTGTACCTGACAACTCA

CGAGCCGGTTCGAAAAAAATTCTGGATGATTTACACGGAGAATTCAGGTCTGGAGAGCTGGTGGCAATATTGGGGCCATCAGGCGCTGGCAAAAGTACTCTCTTAAACTCCCTAG

TCGGTTTTGGAAATGAAGGAATGACGGGAACAATATTGGTGAACAATCAGCTGATGGATGCCAGTTCGTTCCGCAAGCTAAGCTGCTACATCATGCAGAAGGGCGAGCTTCTCCC

TTATCTGACAGTCGGTGAGGCCATGATGGTTTCAGCCAATCTCAAACTGGGAGCTTCAGTCAACAACAGGGAGAAAAAAGTTATTATAGATGAGATTCTGACAGCTATGGGACTT

AACCACTGTACGGAAACTCGATGCAAGAATCTGTCGGGTGGTCAGAAAAAGAGACTGCTTGTAGCTGTGGAGCTTGTAGACAATCCACCTGTCATGTTCCTGGATGAGCCAACAA

GTGGCCTGGACAGCTCGTCGAGTGTGCAGTGCGTCGCGCTGCTGAAATTGCTGGCGCAAGGAGGCCGAACAATAGTGTGCACCATACATCAGCCGAACGCGCGCACATTCGAGA

TGTTCGACCAGTTGTACGTGCTTGCACCGGGCCACTGCATCTATGAGGGCCCTGTACGCTCGTTGGTGCCCTTCCTCGCTAGTCATAGCCTCATCTGTCCCAGCTACCATAATCCGG

CAGATTTCGTTATTGAAGTTGCTATGGGACAACATGGACCAGTACAGCCGATTCTTACCAAAGAGATAAAAAGGCACATGAAGGAAAAATTCAAGGAGGAGGAGAACAGAATA

TCTGACAAGATCAACGAAATTGATCAGCCAAACAATAAAAATTCTATTCCAGGTGAAAGATTTAGAGTATTATCCAGTGAGCTCGAGCTATCTCCAAAAGATGAGAATTCATTCA

ATCGCCTTAGGGGCGATGGGGAGTTTGATGTTTGTGAGAGGCCATCAGCTTTATTTCAATTCTATATTCTATTGAAAAGGACATTCACCTCAACTTCCAGAGATCTTCAACTGACCC

AACTGAGATTGGTATCGCATTTTGCGATCGGACTTCTGATTGGCTACCTCTACCTCAACAAAGGCCAGGACGCCAGCAACATCACCAACAATGCTGGCTGCATATTCTTCACAGCC

ATGTTTCTTATGTTTTCATCCATGATGCCCACTATTCTAACATTTCCATTGGAGATGACAGTCTATAAAAGAGAGCATCTGAACAATTGGTACTCACTTGGCCCATTATATTTGGCG

AAAACGTTGGCTGACATTCCATTCCAGGTGGTGTTCACAGTAGTCTATGTGTCAATAGTCTACTATATGACCGACCAGCCACAGGATTTGGAACGGTTCTCAATGTTCTTATTCGTG

AGCATTTTACTGTCTTTGGTGGCATCATGTGTTGGCCTTTTGACTGGGACAGCTCTATCAATAGAAACAGGCACCTACTTCGGACCAATCTCATGCATTCCCTGCGTCCTGTTCTCCG

GCTTTTTTCTGTCACTAGACTCAATTCCGTCCACAATGAAGTGGCTGGGCAGCCTCTCCTATCTGCGCTACGCGTTCGAAGGCTGTATGCTCTCGCTCTACGGCTTCGACAGATCCA

AACTCGACTGCTCTGAGGTCTACTGCCACTTCAGAATGCCATCTCAGTTCCTCAAACACCTCGGCATGCGAGATGCCTCCTATTGGTACGACTGTAACATTCTAATCATTTTCGTTG

TGGTGCTCAGAACCTTGACCTACTTTGTGCTCAAGTATAAAATCAAGACTCCCTCGTCATAG

NlABCG4

ATGGAACTGGAAACAGAACTGAGGTCCACCTCAGCGGGAACCTATTTCTCGAGGCATGCAGCTAGGGAGCCAGTCGAACTCAGCTTCGACAATCTCACCTACTCTGTTTCACAGG

GTTTCCGAAAAGGCTCAAAAACTATACTACACAACATTGGAGGCAGATTTGAATCAGGTCAAATAATTGCTATTATGGGACCTTCAGGAGCAGGAAAATCATCCCTACTGGATCT

CCTATCAGGATACAGGATATCTGGTGTGGCGGGATCTGTCTACGTGAATGATCGATTCCGAGATCTGGATGAATTTCGAAGACTGTCATGTTATATACAGCAGGACGATCGACTGC

AACCTTTGCTGACTGTTGATGAGAACATGTGGGCAGCTGCTGATCTCAAATTGCCTTCAAGTGTCCCCACAAAAGAGAAAACAGCTATTATAGACGAGATTTTAGAGACCTTGAA

ACTCTCAGGCTCTAAAAAGACCAGAGCCGGACAACTTTCTGGAGGACAGAAAAAAAGACTTTCAATCGCATTAGAACTAGTGAACAATCCATTGGTAATGTTTCTGGATGAGCC

AACTACAGGACTTGATAGCTCTTCGTGTATGCAGTGCGTCACATTACTGAAAGAGCTCGCATCTCAAGGTAGAACAATAGTCTGTACAATTCATCAACCGAGTGCTTCCTTGTTCA

TGAAATTCGACCACGTTTATGTCTTGGCGGGTGGAAGGTGCCTTTATCAGGGATCTTCAAGCAATCTTGTTCCATACCTGGCCGATCTATCGCTACCGTGCCCGACCTACCATAATC

CTGCTGATTACATTATTGAATTGGCCTGTGGAGAGCATGGAGAAGACAAAATTGATAAACTAGTGGATGGAACACAGAACGGCAAATGCTATAAATGGTTCACGAATGGAGAAG

TATTGAAATACAACAATAATGCTGCAGCTGATGTCACTTCTATGTCATGTCTACCCATTATGAAAAAATGTGGAGGCTCACTTCAAGTCACATCTCAATGGAATCAAATCAGTGTC

CTTTTGAGAAGAGGATTCATCAAAATGAAAAGAGATCAGACTCTAACTCACATGCGGTTCATGGTGAACGTACTGACTGGGATGATGTTGGGAGCGCTGTTTTTTCAAACTGGCA

ATAAAGGGGAGAGAGTTTTGGATAACTTTAATTTGCTTTTCTCAATCCTCATACACCACACAATGACAACCAAAGTCCTCACTATTTTGACATTTCCGATGGAAATGTCTATATTGA

ACAAGGAATACTTCAATAGATGGTATTCATTGAAGTCATACTACATAGCTACGAATATCCTCGATATACCAGTGCTAACTGTTTGTGCGATCACATTCTCAGCAATAATCTACGCA

ATGAGTGGTCAGCCATTGGATTGGACCAGATTCAGCATGTTCACTGGCATCAGTCTGTTGGTGGTTTATATTTCGCAAAGTCTCGGTTTCATGGTTGGATCAATATTTAATGTGGTG

AATGGAACTTTTGTTGGACCAACAATGCTCGTTCCCATGATGATGTTCTCTGGATTTGGAGTTTCGCTGAGGGATATTCCAGGATACATGAAATGGGGAACAAATCTCAGTTATCT

GCGTTACAGTCTTGAAGGCTATGTGGCAGCTATCTACGGTTTGAACAGACCAATTCTGCCCTGCACAAGCTATTACTGCCATTACAAATACCCGAAAAAGTTCATGTCGGAAGTTG

CTATGAATGGAGATCAGTTCTGGTTGGATGTTTATGCTCTTCTTTTCACTTTATTCTTGACAAGGGTAGCTGCTTACATTCTTCTCAGATGGAGAATAAGGCAATGA

NlABCG5

ATGGTGGGTCGCCAACAGAGGGATATGGAGAGGAGGTACTCCATAGCAGAAGTTCCTTCGGAGCTGAGTGGAATGCCTCCACCAGGGTTGATGCCTTCAGCCTCTGAGGATCTCC

ATGCTTGGTCTATCTACAGGCAAAATCTGAACTCAGATTTCACAGACTCGGCCCTGGGCTCCAGTGAGAAGTCGCCTCTACCTTACGGCAACTTCCAACTCAGGGAATCAACGGT

GCAATCTATCCTCAGTCATCCCCGCTATGGACCCAAATCAGCGCTCGGTTCCAACATGTACACGTACCTGAAGTTCGGCCTACCTCGGGTGTTTCCGCCGAATGGTGTGAGGGGCG

GAAGAGACGGCAGCAGCGGCTACGACTCCAGTGATGATGGTGGCGGTGGAGGGGGGAACGGTACTGCAGCCAGACCGCGACCTAGGGCTAGACATGCAAGTCAGCAACATTTG

CACGCCCCACCCGGATACTATCTACGCGCCAGGAGTGATCCTGATTTCCGGAACACGCCGTATCATGGGCCAAGCATGCCCCTGAGACAACAGATGGGTGGCCCCGGGGGCGGG

GGTGGAATGCCGCCTCCCCCCCACCAGCAGCACCCAGCCGGGGGCCGTGGCAAGAGTGTGAGTGAGGCCAACCTGCTCGCACCAGAACTGCTGATGAGGCATAATGCGGCTCCC

TATGAACACAGGCGCAGTGTACATGATCTGCGGGGCGCAATCGCCTACTCAGAGCTCGGGGGACCACCGCCGCATGTATTGGTGCATAATGCTAGACACGGAGGTCGTCCAGCTT

CCGTGGCAGTGGTGGGAACAGGACACCACCACCCGGCAGTGTTGCACCGCCACGGCAGTCACTCTGTGTTGGACGGCGGAGTGGGGGGTGGTGCCGCCCCCAGCATGCTCAGTG

GCATGGCACCCCCCTCCTCACGAGCCCACTCAGCCCTCCGCTCTCATGACGCACCGCCAGGCATGTCCTTCCAGGTGCATCGTGGGGAGGCGTTTAGTGGAGCCTATCCGCATTTG

CAGGTTCGAGGTCTAGATGTAGACGGCAAGAACAACGAACCTCTGCTCCAGTCGGTTTCGTTCGAAGCCAAAGCTGGAGAAATCTTAGCAGTCATGGCAACACAAGTTGACGAA

GGTCGTGCCATTCTGGACATCCTGTCGGGTACAAGACGGGCGAGAACCATCCACATAGTGCTGAACGGACAGAGCATCAGCCAGCGAGTGCTGAGGAAGAGGGTGGCCTATGTG

AGGAGTGACTGCACCCTGGCGGGGAGTCTTAGTGTGTCGCAAACGCTTGCATTCTATTCAAGACTCAGGAGGCCTCCCAGGGGTCCCACCAAAGTATCTTCCACCGATCAGATGG

ATCTGCTAATAGAGGAGTTGGGCCTGACTCAGGTGCTAGACACAAAAGTGGCAAGCCTGACCGACTCGGAAGCGCAAAGACTGAGCTTGGCCTGCCATCTGGTGTCCGACGCCG

AAATTCTGCTTCTGGATCGCCCCACGCGGTCCATGGACATTTTCGACACTTTCTTCCTCGTCGAGTTTCTACGACAGTGGGCTGGAGGTAGCAGTACAGGTGGTCTAGTAGGCAGA

ATAGTGGTGCTGACCATCCAGCCTCCAACCTACGAGATCTTCACGATGGTGTCGCGGGTGCTGCTGCTCTCTGGTGGCAGAATGATGTACTCGGGCCGAAGACGAGACATGTTGC

CATATTTCTCCGCCGCTGATTATCCCTGTCCTGCCTTCAAGAATCCTTCCGATTATTACCTTGACCTGGTAACCCTGGATGACCTGTCTGCAGAGGCGATGCTGGAGTCGTCGCAAC

GCATCGAGCAGTTGGCAGAACTGTTTCGGCGCCGACAAGAGCCCCTCTCCGACCCGGGACCACCGCAGGCCCTGCCTGGCAAGACCAGGACTGCCAACCTATGCTCACAGGCTG

TTGCTTTGCTCATGAGACAATTGATCTACTCACAGCCGACCAGCCTGACCAACTGGTTGACTCATGTTCTTCTCGCTGCCATACTTTCACTTATTGTTGGTGCTATATTTTGGGATGT

GCCCAAGTCTGATCCGCAACTTCTCTATGCTGACAGGATTGGATTCCATTATACAATGATGTGCGTTGCTTCACTGCCCATTCTACTGATGCTAACTCTGAGCGACGCTCGCAGTTC

TGAGAGAGCAGCTTCCGAGATGGATATCAGAGATGGTCTCTATTCGAGGCTCATTTTTATCATTATAACAGCTATTATCAGCTTTCCAGCCGTGCTGTTTGTTTGGCTTGCTTATATA

ATTCCAGCCTACGCGATGACTGCATTATATAATCAGGGCTCACAAACACCCAACGGATTCCACATCTATATAAGCACAATGCTGGTACACATGATGTGCCTCTACTACACACTGA

GGCTCATCAGCCAACTGTGGCGGTCCCGGCGGACGGCGGCCATCGCTTCCGGTCTGGTGTTGGTGGTGTTCTCGCTGGTGTCCGGATATCCGGTGTACCTGGCGGATGTGCCCCCC

TGGCAGGCCAACTACTTCGGCCTGGTGTCTCCCGTGAGATGGATGATGCCCAGTCTCCTAGCCAGAGAGTACTCCCCGGTCACTCTGGCCGCCATTGCTTCACAGATGATTTGCAA

CAATCGACAGGTCCAGCAGCAAGACATCATAGTCCAACTGCCCTGTCCTATACCGAACGGCACTGCTGCTCTCTCTTTCTACGGTCTCTCTCCCAAGTCAGCTGTACCCTTCAACTG

GACGCAAGTACTGCCCTACTGGCCGCCAGTGATTATTGCCCTGGCTATGGCTGTTCTGCATACTGTCATCTTCCTCTTCAGATCCCCGACTCCGGCGTGGAAGAAAGAAGATAAAC

TGAAAAGATATATCTATCATCCTCACTAG

NlABCG6

ATGGTGGGTCGCCAACAGAGGGATATGGAGAGGAGGTACTCCATAGCAGAAGTTCCTTCGGAGCTGAGTGGAATGCCTCCACCAGGGTTGATGCCTTCAGCCTCTGAGGATCTCC

ATGCTTGGTCTATCTACAGGCAAAATCTGAACTCAGATTTCACAGACTCGGCCCTGGGCTCCAGTGAGAAGTCGCCTCTACCTTACGGCAACTTCCAACTCAGGGAATCAACGGT

GCAATCTATCCTCAGTCATCCCCGCTATGGACCCAAATCAGCGCTCGGTTCCAACATGTACACGTACCTGAAGTTCGGCCTACCTCGGGTGTTTCCGCCGAATGGTGTGAGGGGCG

GAAGAGACGGCAGCAGCGGCTACGACTCCAGTGATGATGGTGGCGGTGGAGGGGGGAACGGTACTGCAGCCAGACCGCGACCTAGGGCTAGACATGCAAGTCAGCAACATTTG

CACGCCCCACCCGGATACTATCTACGCGCCAGGAGTGATCCTGATTTCCGGAACACGCCGTATCATGGGCCAAGCATGCCCCTGAGACAACAGATGGGTGGCCCCGGGGGCGGG

GGTGGAATGCCGCCTCCCCCCCACCAGCAGCACCCAGCCGGGGGCCGTGGCAAGAGTGTGAGTGAGGCCAACCTGCTCGCACCAGAACTGCTGATGAGGCATAATGCGGCTCCC

TATGAACACAGGCGCAGTGTACATGATCTGCGGGGCGCAATCGCCTACTCAGAGCTCGGGGGACCACCGCCGCATGTATTGGTGCATAATGCTAGACACGGAGGTCGTCCAGCTT

CCGTGGCAGTGGTGGGAACAGGACACCACCACCCGGCAGTGTTGCACCGCCACGGCAGTCACTCTGTGTTGGACGGCGGAGTGGGGGGTGGTGCCGCCCCCAGCATGCTCAGTG

GCATGGCACCCCCCTCCTCACGAGCCCACTCAGCCCTCCGCTCTCATGACGCACCGCCAGGCATGTCCTTCCAGGTGCATCGTGGGGAGGCGTTTAGTGGAGCCTATCCGCATTTG

CAGGTTCGAGGTCTAGATGTAGACGGCAAGAACAACGAACCTCTGCTCCAGTCGGTTTCGTTCGAAGCCAAAGCTGGAGAAATCTTAGCAGTCATGGCAACACAAGTTGACGAA

GGTCGTGCCATTCTGGACATCCTGTCGGGTACAAGACGGGCGAGAACCATCCACATAGTGCTGAACGGACAGAGCATCAGCCAGCGAGTGCTGAGGAAGAGGGTGGCCTATGTG

AGGAGTGACTGCACCCTGGCGGGGAGTCTTAGTGTGTCGCAAACGCTTGCATTCTATTCAAGACTCAGGAGGCCTCCCAGGGGTCCCACCAAAGTATCTTCCACCGATCAGATGG

ATCTGCTAATAGAGGAGTTGGGCCTGACTCAGGTGCTAGACACAAAAGTGGCAAGCCTGACCGACTCGGAAGCGCAAAGACTGAGCTTGGCCTGCCATCTGGTGTCCGACGCCG

AAATTCTGCTTCTGGATCGCCCCACGCGGTCCATGGACATTTTCGACACTTTCTTCCTCGTCGAGTTTCTACGACAGTGGGCTGGAGGTAGCAGTACAGGTGGTCTAGTAGGCAGA

ATAGTGGTGCTGACCATCCAGCCTCCAACCTACGAGATCTTCACGATGGTGTCGCGGGTGCTGCTGCTCTCTGGTGGCAGAATGATGTACTCGGGCCGAAGACGAGACATGTTGC

CATATTTCTCCGCCGCTGATTATCCCTGTCCTGCCTTCAAGAATCCTTCCGATTATTACCTTGACCTGGTAACCCTGGATGACCTGTCTGCAGAGGCGATGCTGGAGTCGTCGCAAC

GCATCGAGCAGTTGGCAGAACTGTTTCGGCGCCGACAAGAGCCCCTCTCCGACCCGGGACCACCGCAGGCCCTGCCTGGCAAGACCAGGACTGCCAACCTATGCTCACAGGCTG

TTGCTTTGCTCATGAGACAATTGATCTACTCACAGCCGACCAGCCTGACCAACTGGTTGACTCATGTTCTTCTCGCTGCCATACTTTCACTTATTGTTGGTGCTATATTTTGGGATGT

GCCCAAGTCTGATCCGCAACTTCTCTATGCTGACAGGATTGGATTCCATTATACAATGATGTGCGTTGCTTCACTGCCCATTCTACTGATGCTAACTCTGAGCGACGCTCGCAGTTC

TGAGAGAGCAGCTTCCGAGATGGATATCAGAGATGGTCTCTATTCGAGGCTCATTTTTATCATTATAACAGCTATTATCAGCTTTCCAGCCGTGCTGTTTGTTTGGCTTGCTTATATA

ATTCCAGCCTACGCGATGACTGCATTATATAATCAGGGCTCACAAACACCCAACGGATTCCACATCTATATAAGCACAATGCTGGTACACATGATGTGCCTCTACTACACACTGA

GGCTCATCAGCCAACTGTGGCGGTCCCGGCGGACGGCGGCCATCGCTTCCGGTCTGGTGTTGGTGGTGTTCTCGCTGGTGTCCGGATATCCGGTGTACCTGGCGGATGTGCCCCCC

TGGCAGGCCAACTACTTCGGCCTGGTGTCTCCCGTGAGATGGATGATGCCCAGTCTCCTAGCCAGAGAGTACTCCCCGGTCACTCTGGCCGCCATTGCTTCACAGATGATTTGCAA

CAATCGACAGGTCCAGCAGCAAGACATCATAGTCCAACTGCCCTGTCCTATACCGAACGGCACTGCTGCTCTCTCTTTCTACGGTCTCTCTCCCAAGTCAGCTGTACCCTTCAACTG

GACGCAAGTACTGCCCTACTGGCCGCCAGTGATTATTGCCCTGGCTATGGCTGTTCTGCATACTGTCATCTTCCTCTTCAGATCCCCGACTCCGGCGTGGAAGAAAGAAGATAAAC

TGAAAAGATATATCTATCATCCTCACTAG

NlABCG7

ATGAAGATCGATGAAAGTGAGTGCTGTGAGATGAACCAGATTGTTTCTGCTGACAGGAAGGGCTCGGTGAAGGTGAACATCCAGCCATGCCAGCAGGCGCGCACCCTCACCCAT

CTGCCCAAGCGGCCGCCCGTCGATATCGTCTTCGAGGACCTCACCTACTCCGTCTCCGAGGGCAGGAAGAACAAAACAAAGAAGATCTTGAAATCTGTAAGCGGCAGACTTTAC

TCTGGCGAACTTACTGCCATTATGGGGCCCTCAGGGGCCGGAAAGTCAACTTTGCTCAACATTTTGACTGGTTACAAGACTTCGGGAATGAAGGGTTCAATCAGAATCAACGGAG

AAGAAAGAAATTTGAGCCAATTTGCAAAATTGTCGGCCTACATCATGCAAGACAACCAACTTCACGGCAACCTCTATGTTGAAGAAGCAATGCATGTCGCCGCCAATCTCAAATT

GGGCAACGAACATAGCAAAGAGGAAAAATTGGATGTGATCCAGGAAATTCTGGAAACATTGGGACTACAAGAACACAGGCGCACCCTCACATGCAACCTGTCTGGTGGACAAA

AGAAGCGTCTCTCAATTGCTCTCGAATTGGTCAACAATCCTCCAATCATGTTCTTCGACGAACCTACTAGTGGTCTGGACAGCTCATCGTGCTTCCAGTGCGTGGCTCTGCTGAAGT

CGCTGTCGAGCGAAGGTCGCACGATAATCTGCACGATCCATCAGCCGAGCGCCCGCCTCTTCGAGATGTTCGACCACCTGTACACGCTGGCCGACGGCCAATGCGTCTATCAGGG

CTCCACCGCCCAGCTGGTGCCCTGGCTCAAGACGCTCAACCTCGTCTGTCCCAGCTACCACAACCCAGCTTCCTTCATTATTGAAGTATCTTGCGGTGAACACGGCGAAAATGTTC

GTAAACTGATGGCAGCAATCAACAATGGCAAAAATGACATCAGAACTGGCAAGCCATTCCCGAAAGTAGATTTGATGGCTCTGAACAACTCCAACATGGAGAAACAGTCCAAC

CTGAGTCAGGAAACTGACAATCTGCTGGCCAACGGAGATGCCACTTCCGACTCCAACCAGCCTCTTTCTGAGGGAAGCAACTGCACCAATAACATGCTGCTAGCTTATGCTACCA

ATGACATTGCCAAGGATTCTCAATCTCATTCTGATGGGAAAGGAGGAGTTGTGATTCCAGTGGATTTGGGTGACAGCGAGAAGGGCAAGTGTGACAACGTTTCCACGAGTTTGTT

GGAAACGTCACTTCCATTGTCCCAAAAGAGATACGGCACATCGGAATTCAATCAGTTCTGGATCGTTTTGAAGAGAACTTTGCTCTTCTCACGCAGAGATTGGACACTTATGTACT

TGAGATTGTTTGCTCACATTCTGGTTGGCTTTCTGATTGGCGCTCTCTACTACGACATTGGAAATGACGGAGCCAAAGTGCTCAGTAACCTTGGATTCTTGTTCTTCAACATGCTCTT

CCTTATGTACACATCGATGACCATCACTATTCTCTCCTTTCCTCTTGAGATGCCTGTGCTGATAAAAGAAAACTTTAATAGATGGTACTCGCTACGATCCTACTATCTCGCCATAAC

TGTTTCTGACATACCATTCCAGGCTGTATTCTGCGTACTGTATGTGTCAATTGTGTACTACTTGACATCGCAGCCGCAAGACCTATCTCGTTTTGGAATGTTCTTGGGAGCTTGTCTG

CTCATCTCGTTTGTCGCGCAGAGTGTTGGACTGGTCGTGGGAGCCGCTATGAATGTTCAGAACGGTGTATTTTTGGCTCCTGTGATGTCGGTGCCCTTCCTACTCTTCTCCGGATTCT

TTGTGAGTTTCGACGCTATACCGGTCTATTTGAGATGGATAACATATCTCAGTTACATCAGATACGGATTCGAGGGAACTGCACTTGCTACCTACAGTTTCAACCGAACAAATCTT

AAATGTTTCCAGGTATACTGCCACTTCAAGGACCCCAACACCACACTGGAAGAGCTCGACATGAAATCTGCCAGCTTCGAACTAGACATCATTGCTTTAATTGTAATATTCTTTTT

CTTGAGAATCTCAGCATACTTATTCCTTAGGTGGAAACTGATGTCGTCTCGTTAA

NlABCG8

ATGGAGGGTATCAACTCGAATAAAACTAAACACGACATGGAGCTCTACAATAACAATGACTACGATCAGTTCAGCTTCCCCAAACGACCCACAGTGGATATTAATTTTCAGGAC

ATCACGTATACTGTGAATACAATGACAGCAAAAAAGGAGATTCTGCACGGTGTGAGTGGAGAGTTCAGGTCTGGAGAGTTGACCGCCATCATGGGGCCTTCGGGTGCGGGAAAG

AGCACCCTACTCAACATTCTGGCTGGATTTACGTTGAAAGGCTGCCAAGGAAGTATATGCATCAATGGTATTAACAGGAAAAGTCGAATTGAACAATTCCTGAAGATGTCCTGCT

ACATTACACAAGATGACGAACTGAGGCCTCTATTGACTGTCAGAGAAGCTATGATGCTAGCAGCGCATTTGAAACTCGGTTTCACTCGATCCAACCCTGACAAGTCCAACCTGGT

ATCATATATATTGGGTCTTTTGGGACTAAAAAAACATGAGAATACTAGGACATCTCGATTATCTGGCGGACAACGTAAAAGGTTATCAATTGCTCTCGAGCTGCTCACCAACCCTC

CAATCCTGTTCCTTGATGAGCCAACAACGGGACTAGACAGTGTGTCAACCACATCGTGTGTTTCGCTGCTCAAGAATCTGGCGGCTGAGGGACACACGATCGTCTGCACAATCCA

CCAGCCGACTGCCTCGATATTCGAGATGTTTGACCACCTGTATGCAATTGCAGACGGCGACTGCATCTACCAAGGCTCCTGCAGCAATCTGCTGCCGTTCCTGTCCTCATTAAGTCT

GCATTGTCCCAAATACCACAATCCTGCAGACTTTTTAATCGAGGTGGCAATTGGTGAATACGACACAAATGTGAAGACGATAGCTGCAGCGGCAGCTAAGCATGGTCGCCAAGA

GTCCACACCTTATTCCGAAGAAATAATCAAAGATGAAAATGGTCTGCCAGTTAGAGTATTGCAAACCAAAAAGTCGCCAGCTTTTGACATAATTGAGTACACTTCATGTTTAGCA

GAGCCGGCTCCGTTATGGTATCAAGTATTTCATCTTCTCCATAGAAACATAATTATCACACGTAGGTCAAAGTTGCCGCTTGCCTTACGAATTTTTATGCACTTTGTGATCTCAGTA

ATGTTTGGAATCATTTACAATAATGTTGGCAACAACGCAAACTCTGTATTTGGAAATTACATTTACGTTTATGGAACGAACTTGTTTTTGCATTATACCGGACAAATGGCAGTTACT

TTGTCGTTTCCATTGGAGTTCAAAGTGCTGAGGAGAGAGCATTTCAATAGATGGTATTCTCTGATTCCTTACTGCATAGCCACGCTACTAATCGAGATACCGTTTCAGATTGTTTGT

GTTGTCGTGTACTTGGTGCCTAGCTACCTGTTGACTGGACAGCCCCTGGAGTGGGTCAGGTTCTTGATGTTTCTAATGTTCACGGTTGCAGTTTGTCTGACTGCGCAAGCGTGTGGAT

TTTTGGTTGGAGCCACAACGCCAGTAACGTTGGCTGTCTTCATTGGACCAGTGATCACTGTATTCCTATCAGTGTTCGGATTCGCAATGAAGTACAGCGACATTCCGTCCTACCTGC

GGGTCTTCTATCACATATCATACTTCCGATCGTCCTTCCAAGGATCACTGATGAGCTTGTACGGTAACAACCGGTCCTACCTGCCGTGTCTGAAGAACGGGTTTCACGGCCGTAAC

GGCTACTGCCACTACACGCATCCGACAAAGTTTCTCAGGGAAATGGAGTTCGAAGAACCAAACCCTGTGTTCGATGTCAGTTACATTGTTTCAGTTTGTTTACTGGTGTACACATGT

ACAGCGACTGCTATCTGGTATAGATTGAACAAAAGGTGA

NlABCG9

ATGGACGTGGAATTCGAGGATTTGACAGTCAGAGTGAATAGCAGTTGGTTTAATAGAGATTCTGGAAGAAGAATTTTGAAAGGAGTGAGTGGCAAATTTAAAGCTGGTCAGCTAT

CTGCCATACTTGGACCTTCAGGCGCCGGAAAAAGTTCTCTACTCAATGCAATATCTGGTTACAGATCGCAAGGCGTGAGTGGCCGTTTGAGACTGAATGGAGTGGCAAGAGATGA

AGCCAGGTTCAGAAAGATGTCGTGCTATATCCAGCAGGAGGACCTTTTGCAGCCAATGCTCACTCTCCAGGAAGTCATGAACTTTGCAGCCTTACTCAAACTTCCGCCAGGAACT

GGATACAAGCAGAGAAGAGTTGTGGTGAATGATATTCAAGGAATTTTGGGCTTGACTGAATGTAGACATACAAGAACTGAGGCGCTGTCTGGTGGCCAGAAAAAGAGACTATCT

ATAGCCCTAGAGCTGATCAACAACCCTCCAGTCTTATTTCTAGACGAGCCTACGAGTGGCTTGGACAATGTGTCGACGTCGTACACTCTGCGTCTGCTGCGAACCCTGGCCCACCA

GGGCAGAACGATAGTGTGCACCATACATCAGCCAAGTGCCAGCCTCTTCCAGATGTTCGACCACGTGTATGTGCTGGCCTCGGGGCTCTGTGTGTACCAGGGCGTAACAGGCGAG

CTCGTTCCCTTCCTATCCTCGGTCGGTTTACACTGTCCCAGACATTACAACCCGGCTGACTTCGTAATTGAAATGACTGACGGAGATGACGAGGATAATATCAAAAGATTATCATC

AGCGATAAAAAATGGAAAAGTTGTCCAACTGACGTCTGCTGATGCCAAAAAGTCAATTCCAGATTTTTCAAATCTACCAATAGAAGGGCTACCACTTGAAGAGAAAATAGTTGC

AGTCACTGGTTCTGGAGAGAAATACGTAGACATGGACAACGGCATCTGTCTCACTTGCAGGGCAGACTCCTCTTCTTGGTTAGAATTCTGTACGCTTTTCCGAAGGATGTTCCTTCA

AATTATGAGAAATAAGACTGGTCTAAAAATCCAATTTTACCATCACTTGGTGTGTAGTCTGGCTGTGGGCATTGTATTCTGGGGCAAGGCTCGCGACGGAAATCAGTTCTTCAACC

ATATGAAGTTCTGCATGGGCATCATTCTATTCCACGCCTACACTCAGTGTATGGTACCTGTTCTTACCTTTCCATTCGAAGTGAAACTTTTGAAGAAAGAACACTTCAACCGATGGT

ATCGACTCACGCCCTACTACATGGCTCTACAACTCTCCAAAGTTCCAACCATGACGATATTCAGCCAGTTGTTCCTGACAATAGTGTACGTGATGTCAGGTCTACCAATGGAGTTC

TACAGGTTTTTCGTTTTCTCAGTGGTTGGCGTCATGACTGCATTTGTGGCTGAGGGATGGGGTCTTGCCATTGGATCTGTATTCAATGTCACTAACGGCAGCGCAGTGGGACCGATG

ACAATTGCGCCGTTTCTCGGATTCGCGATCTACGGCTTCGACTTTGCCCGAGATATTCCGGCCTGGTTCATGCCCATCCTCAAGCTGAGCTTCCTCAGATCGGGCGTCATCGCTCTC

ATCATCGTGGTGTTCGGCATGAACAGGGGTTTCCTCGACTGCAACCACGAGATGTACTGCCACTTTAAGAATCCTCGCATCATCATCTACTACCTGGACATCGAAGGCGTATCGCC

CTGGCAGGAGATACTCGGCATGTTTGCCATGCTCCTCTTCTTCAGAATTGTCTGCTTTTTTGGACTAAAATGGCGGCTCAGAACTTGA

NlABCG10

ATGGTGGGTCGCCAACAGAGGGATATGGAGAGGAGGTACTCCATAGCAGAAGTTCCTTCGGAGCTGAGTGGAATGCCTCCACCAGGGTTGATGCCTTCAGCCTCTGAGGATCTCC

ATGCTTGGTCTATCTACAGGCAAAATCTGAACTCAGATTTCACAGACTCGGCCCTGGGCTCCAGTGAGAAGTCGCCTCTACCTTACGGCAACTTCCAACTCAGGGAATCAACGGT

GCAATCTATCCTCAGTCATCCCCGCTATGGACCCAAATCAGCGCTCGGTTCCAACATGTACACGTACCTGAAGTTCGGCCTACCTCGGGTGTTTCCGCCGAATGGTGTGAGGGGCG

GAAGAGACGGCAGCAGCGGCTACGACTCCAGTGATGATGGTGGCGGTGGAGGGGGGAACGGTACTGCAGCCAGACCGCGACCTAGGGCTAGACATGCAAGTCAGCAACATTTG

CACGCCCCACCCGGATACTATCTACGCGCCAGGAGTGATCCTGATTTCCGGAACACGCCGTATCATGGGCCAAGCATGCCCCTGAGACAACAGATGGGTGGCCCCGGGGGCGGG

GGTGGAATGCCGCCTCCCCCCCACCAGCAGCACCCAGCCGGGGGCCGTGGCAAGAGTGTGAGTGAGGCCAACCTGCTCGCACCAGAACTGCTGATGAGGCATAATGCGGCTCCC

TATGAACACAGGCGCAGTGTACATGATCTGCGGGGCGCAATCGCCTACTCAGAGCTCGGGGGACCACCGCCGCATGTATTGGTGCATAATGCTAGACACGGAGGTCGTCCAGCTT

CCGTGGCAGTGGTGGGAACAGGACACCACCACCCGGCAGTGTTGCACCGCCACGGCAGTCACTCTGTGTTGGACGGCGGAGTGGGGGGTGGTGCCGCCCCCAGCATGCTCAGTG

GCATGGCACCCCCCTCCTCACGAGCCCACTCAGCCCTCCGCTCTCATGACGCACCGCCAGGCATGTCCTTCCAGGTGCATCGTGGGGAGGCGTTTAGTGGAGCCTATCCGCATTTG

CAGGTTCGAGGTCTAGATGTAGACGGCAAGAACAACGAACCTCTGCTCCAGTCGGTTTCGTTCGAAGCCAAAGCTGGAGAAATCTTAGCAGTCATGGCAACACAAGTTGACGAA

GGTCGTGCCATTCTGGACATCCTGTCGGGTACAAGACGGGCGAGAACCATCCACATAGTGCTGAACGGACAGAGCATCAGCCAGCGAGTGCTGAGGAAGAGGGTGGCCTATGTG

AGGAGTGACTGCACCCTGGCGGGGAGTCTTAGTGTGTCGCAAACGCTTGCATTCTATTCAAGACTCAGGAGGCCTCCCAGGGGTCCCACCAAAGTATCTTCCACCGATCAGATGG

ATCTGCTAATAGAGGAGTTGGGCCTGACTCAGGTGCTAGACACAAAAGTGGCAAGCCTGACCGACTCGGAAGCGCAAAGACTGAGCTTGGCCTGCCATCTGGTGTCCGACGCCG

AAATTCTGCTTCTGGATCGCCCCACGCGGTCCATGGACATTTTCGACACTTTCTTCCTCGTCGAGTTTCTACGACAGTGGGCTGGAGGTAGCAGTACAGGTGGTCTAGTAGGCAGA

ATAGTGGTGCTGACCATCCAGCCTCCAACCTACGAGATCTTCACGATGGTGTCGCGGGTGCTGCTGCTCTCTGGTGGCAGAATGATGTACTCGGGCCGAAGACGAGACATGTTGC

CATATTTCTCCGCCGCTGATTATCCCTGTCCTGCCTTCAAGAATCCTTCCGATTATTACCTTGACCTGGTAACCCTGGATGACCTGTCTGCAGAGGCGATGCTGGAGTCGTCGCAAC

GCATCGAGCAGTTGGCAGAACTGTTTCGGCGCCGACAAGAGCCCCTCTCCGACCCGGGACCACCGCAGGCCCTGCCTGGCAAGACCAGGACTGCCAACCTATGCTCACAGGCTG

TTGCTTTGCTCATGAGACAATTGATCTACTCACAGCCGACCAGCCTGACCAACTGGTTGACTCATGTTCTTCTCGCTGCCATACTTTCACTTATTGTTGGTGCTATATTTTGGGATGT

GCCCAAGTCTGATCCGCAACTTCTCTATGCTGACAGGATTGGATTCCATTATACAATGATGTGCGTTGCTTCACTGCCCATTCTACTGATGCTAACTCTGAGCGACGCTCGCAGTTC

TGAGAGAGCAGCTTCCGAGATGGATATCAGAGATGGTCTCTATTCGAGGCTCATTTTTATCATTATAACAGCTATTATCAGCTTTCCAGCCGTGCTGTTTGTTTGGCTTGCTTATATA

ATTCCAGCCTACGCGATGACTGCATTATATAATCAGGGCTCACAAACACCCAACGGATTCCACATCTATATAAGCACAATGCTGGTACACATGATGTGCCTCTACTACACACTGA

GGCTCATCAGCCAACTGTGGCGGTCCCGGCGGACGGCGGCCATCGCTTCCGGTCTGGTGTTGGTGGTGTTCTCGCTGGTGTCCGGATATCCGGTGTACCTGGCGGATGTGCCCCCC

TGGCAGGCCAACTACTTCGGCCTGGTGTCTCCCGTGAGATGGATGATGCCCAGTCTCCTAGCCAGAGAGTACTCCCCGGTCACTCTGGCCGCCATTGCTTCACAGATGATTTGCAA

CAATCGACAGGTCCAGCAGCAAGACATCATAGTCCAACTGCCCTGTCCTATACCGAACGGCACTGCTGCTCTCTCTTTCTACGGTCTCTCTCCCAAGTCAGCTGTACCCTTCAACTG

GACGCAAGTACTGCCCTACTGGCCGCCAGTGATTATTGCCCTGGCTATGGCTGTTCTGCATACTGTCATCTTCCTCTTCAGATCCCCGACTCCGGCGTGGAAGAAAGAAGATAAAC

TGAAAAGATATATCTATCATCCTCACTAG

NlABCG11

ATGACGGAGGAACGTGCTGTTTTACTACACTTGCCTCCCAGCAAACCTATTCACATATCGTTCACTGACATCACACTCACCGTCGACTTGGGCACTGTACGAAAAACGAAAAAGC

AAGTGCTGAAAGGTCTTGCTGGGAGTTTCAACTCAGGAGAGCTGACGGCCATAATGGGTCCATCGGGTGCTGGGAAATCATCACTACTCAACATCCTAACCGGCTTTCAGAAACA

AGGCATGACTGGTACAATAACAACGAGCGGAGCTGGAAAAATAGAGAACTATTTCAAAGATGGCGTCAACACAAAACAGTCCTGTTACATCATGCAAGATGATCAACTGAACC

CTCTGTTCTCAGTTTTTGAAATAATGTCGATGGCTGCTGATTTAAAATTGAGTCCAGCTATATCACAGAAATCGAAGATACTTATTATTGATGATATTTTGGAGACAATTGGTCTGA

TGGGTTGTAAGTACACACGGTGTGGCAGATTATCGGGAGGCCAGAAGAAAAGGTTGTCCATAGCTCTGGAACTTGTTGACAATCCTCCGATCATGTTTCTAGATGAACCAACGAC

AGGATTGGACAGCTCAAGCACGGTTCAACTGGTTTCTCTGCTAAAATGTTTGGCAAGAGGCGGAAGAAATATAATTTGCACAATCCATCAGCCGAGCGCAACAATCTTTGAAATG

TTCGACCATGTTTACCTGATCAATGGCGGACGATGTGTGTACCAGGGTTCCAGCATCAACTTGGTCAAGTTTCTGCAGTCAATCAACATTCCTTGTCCCAAGTACCATAATCCTGCC

GATTTTGTAATGGACGTGATAAGGTCTACCTCACGAGGTGTTCGTCTGCGAGTTCTGCCTGAAGACCTACAAGTACAAGCACACCCTGGCGAGGCACCAGAAGATAGAGTGCGGC

CGCAATCCGCAGCTGGAGTGCACCTACTGCAACAAGAAGCTCATGCACAAACATAA

NlABCG12

ATGTGTGCCAAACGTCATGGACAAGTTACCATCGATTTCGAGAATTTGTCTTACAGTGTACCTGAGACAAATAAAAAATATCCTGACCGTGTTCTTCTACACAACGTTAGTGGACA

CTTCCGTCCGAAGCATTTAGTGGCCTTAATTGGTCCATCAGGAGCTGGTAAAACTACCCTTCTAAATGTAATATCTGGTTCAAAGGCAGTAGACAATTCGCGAGTGTCTGGGAAGA

TACTCGTAAATGGAAAAGATAGAAATTTGCAAAAGTTTAAGAAACAATCCTGTTATATTACACAGGAATGGTCTCTACTGAACCAGCTGACTGTAGAAGAGACTTTGGAAATTGC

CGCTAGATTCAAGCTGCCAAGCAATATCAGTGAAATTGATCGGAAAAGTTCGATCAATGAAGTTGTTGAAATTTTGAGACTGAATGGATCTAGGAACACTCTGGTAAAAAATCTT

TCAAATGGTCAGAAGAAACGAATTTCAATTGGTGTTGAACTTATGAATAATCCACCTGTTTTGTTCGTGGATGAGCCAACAAGTGGGTTAGACAGTTCATCAGCTCTGCAAGTGGT

TAATCACCTTCAGAGTCTGGCATTGGACGGCCGAACTGTGATAGTAGTGATACATCAGCCGAGCTCCAAAGTGTTCCAGCTGTTCCATGATGTCTACCTGCTGTCTGATGGTGAAT

GCCTGTACAATGGTCCCTCCGAGCACTTGGTTGCTGCCCTCTCGTCAGCTGGCTTCAATTGTCCACAGTATTACAGCAAGTCTGATTTCGCGATCGAAGTCGCCAGCGCAGAGGTA

GAAGGAGATGTAAGATTATTAAAGATGGAAACAAAGAAACGCTATGAAATGAAAGAAGGAAAATTTTATGAGAATGAAATACCTGCAAAGCGAATTTCAGAAGCGAAGAATG

CAAATGAGCACACAACAATGATGATCGGTGAAGATGACGATGATGATTCCTATAATAACTCTTTGAGAGGTTATCCAGTTTCAAAACTTCAACAGTTCTGGATACTCTTCAAACG

ATGCACTCTTTGTACGAATAGAGATATGTATTTATCTCGAACTCGATTAATTACGCACATTTTGGTTGGCATCATGCTCGGCTGTCTCTTCTACAACTTTGGAAATGACGCCGATAA

AGTAATAGGAAACTATTCCTTCTATTTCTTCGCCGTATTATTCATCTACTTCAGCAGTACTATGCCGGCCATAATGACATTTCCGGTTGAAGCCAACGTATTCCTTAGAGAGCATTC

AAACAATTGGTACTCTCTTACCGTCTATTTCTTTGCAAAAGTATTAGCGGATCTACCCTTACAGATAATCTGTCCGACTTTATTTTTGGTGATTGGTTACTCCATGACAGGCCAGCCA

ATGGAATTACAGAGATTCTCCATGATTTGGTTTGTGATGGTTCTACTGTCCATCCTTGGCCAGTCGTTCGGCAATGCGGCTGGTGCTCTGCTCAATGTCGAGCTGGGCATCTTCATTG

TGCCGTCGATTGCGATGCCTCTGACGCTGCTGTCGGGTTTCTTCCTGCAGCCGAAGGACCTGTCGGCGACGGTGAAGACGCTTAGTTGCGGCAGCTACTTCAAGTACGCATTCGAA
GCGATCGCCGTGTCGGCCTTCGGCTACGACCGTGGTCGGCTGCCCTGCTCGCAGCCCTACTGCCACTACCGCAGCCCGGCCAAGTTCCTCGCCGACATCGGCATCGATGACTATCA
CTACCTGCAACGCGTCACCGTCGTCCTCGTTTGGGTCCTAGTCACGCAAATGGCGCTCTACTGCACGCTCACCATCAAGGTCTACCAGGTCAAGATCAACACTGCTCTGCGCAGGC
TCATGAGATGA

NlABCG13

ATGATAATTTCCAAGGATCTATTCCTGCTGGGTGACCAAGACTATCTGCGTCTTCCCAAGGACGAGAAGCGGACCATGGTGCGCCCGACCTCGGCCAAAACTCAAAGGGTGGAAT
CGCATTTACCATGTCTTCAGCACCATCACTTGCCTCACAGACCACTGGAGCTTGTCTTCACCAACGTATCCTACGTTGTGGACAAAAAAGCTATCCTGAAGGACATCAGTGGTGTT
GTCAAACCAGGAGAGCTGTTGGCTGTCATGGGCCCTTCAGGCTGCGGCAAAACGACCCTTCTAAATTGCCTGGCCGGCCGATTAAAGTTAGATTCCGGAAATATTCGTCTGAACA
AAGAACGCCTTAACAAGCGCTGGAAAAGGCGGATATGCTACGTTTTGCAGCAAGATATTTTCTTCCCTGATCTCACACTTCGTCAGACCCTAGAGTATGCAGCGATGTTACGGTTA
CCTGATTCACTATCTCACGCCCAAAAAATGCAATACGTTGATCACATAATTGATGTTTTGGATCTTACGAATTGCCAAGAAACAATTATAGGAGATTACATAAAAAGAGGACTGT
CCGGGGGAGAGAAGAAGCGCGCCAACATTGCCTGTGAACTACTAACCAATCCCTCATTGATGCTCCTCGATGAGCCAACTTCAGGACTGGATTCACATTCTGCTTACAATTTAAT
GTTGTCATTAAAAAAGTATGCGGAAAAAGAAGGAAAAACAGTGGTTGTCACTGTTCATCAACCCTCTTCTCAAATATTCCACATGTTCGACAGACTATTATTACTTTGCAATGGAG
AGACTGCCTATTTTGGGGACGTAAATAAAGTTGTTGACTTTTTCAACAACGTTGGACTGACGATGATGCCTCATTACAACCCTGCTGACTTTATTTTGGAGCAAGTAAAAGGAAGT
GAAGAAATGAAAGAGAAAATCATCACAGCGGCGAGAGAAGCCAGATTTCGACCCAACTATCCCCAAGAACTGATGCCGGAATATTTCAACCAATCAATGTATCTAAATAACTTT
CACGAAAGTCATTTACATTCGAATGGTCACATAGGAGGAGTACGTTGTCAATGCCAGCGAGAATTATGGAACAATTCAAGGCACTCGACATCACAGAATCTACCCTCTTCCGAAA
TGTGTGTCCCGGTCGCCGTTGCGATAGGCGAAACAACCGAATCGCAGGGCCATGTTTACACAACTATTGCAGTGAAAGAAGAAGAGGGTAAAACCCTGTGGCAGGATACAGCCA
GTCACGCGTCTTCCTCGGTTAGCAGTTCTGACGACGATGTGTCGTGGCAGTGGCCCACTTGTTTCTGGACTCAATTCAAAGTACTGAGCAGAAGAAATTTCCAAGAAGCAAGACCC
AGAATGCTTTCAACTTTGAACTGGGTGCAAACGGTAGCTTTAGGAGTGATGGCTGGACTTCTCTGGTTTCAACTGGAGAGGAAAGAGGAGTCACTACATGACATCCAGGGATGGA
TGTTCTTCTCAACGACGTATTGGATGTTGTTCGCTCATTTTGGAGCTCTATCGTCATTTCCACCTGAAAGAGAAGTGATCAACAAAGAACGTCTGTCTGGAGCATATCGACTGTCGG
CCTATTACCTGGCGAAAATGGTGGGCGAATTGCCGTTGACCATCACGCTGCCAGCAGTCTATCACATCATATCCTACCCAATGCTTGGCTTCCACAGTCCTACCGTGTTTGCTACAC
TGCTGGGATTCTTGCTTCTCAACACCATTGTAGCTCAGAGTGTTGGATTCTTTGTCGGCGCATGCTGCATGGACATGCAGGTGTCGATAACGATCAGTGCCCTCTACACGCTGGCCA
CGCAACTGTTTGGCGGTTACCTGGCCACCAACATCCCGCCCTGGCTCAAGTGGATGCAGTATTTGTCCATGGTCCATTACGCCTATCAGAATATGCAGATTGTCGAGTTCAGCGAA
GGAGAGTGGATCAAATGCGCGCCGCAATCGAAGTTCGACGTGTGCCTCAACAACTCGACGACGCACATCCCCGTGGCGTCAATCCTGGAGGTGCAGGGTGCCAGCCTGCCGCTC
TGGGCCAACACCCTCGTGCTGCTCCTTTTCCTGCTCATATTCCGCGTGCTTGGCTACATCGTGCTCAGATACTTCCGTCGACCCAAGTGA

NlABCG14

ATGATTGGCAACGACTATAGTTTGGAGCTGTGTAATATTTTTCACACTGGACAGGTTGAGCCAGGCTCATGTTTGCAGAGAATATTTGGCAGCGTGCAGACTGGCCTGATTCTGAA
AGATGTTTCTCTCGAAGTGAGAGCGGGAGAGGTCCTCGCAGTTCTTGGATCGAAAGGAAGTGGTAAACGAGCTCTTCTGGAAGTGATATCTAGGAGAAGTCGTGGTCCCACCAGA
GGACAAATCCTCCTCGATGGAGCACCAATGACCTTGAGTTTGTACCAGAAGAATTGCGGCTACGTCAGTCACAGAGTTGACCTTATTCCGTCACTAAATGTGGAGCAAACTCTAC
ACTATGCGGCCAACCTCACTATTGGATCACAGGTATCACGGTACGTGAGAAGCTCTCGAGTTCGTCAAGTGTTGGCAGATTTGGCTTTGAGTCAAGTGGCTCGGCGAAGTGTGGCA
AGTCTCACACTCAGTGAATACAGGCGCCTTGCCATTGGTATACAACTCGTCAAGGATCCTGTTCTGCTGCTATTGGACGAGCCGACCGCTAACCTTGACCCTCTGTCGACCTACCT
GATAGTGTCGATGCTATCCTCACACGCCAGACGGAGGGGGAGGGCGGTGGTGTTGACCATGGAGAAGCCGCGATCCGACGTGTTCCCCTTCCTCGACAGAGCCGCCTACCTCTGT
TTGGGGGATCTCGTCTACGCGGGACCCACTCGCCTCATGCTCGAGTACTTCAGGGCTATCGGCTTCCCCTGTCCTGATCTCGAGAATCCTCTCATGTATTACTTATGCTTATCAACA
GTGGACCGACGTTCGAGAGAACGCTTCATCGAGTCCAACACCCAGATCATAGCTCTTGTGGAGAAGTTCAAACTGGAAGGAGGTCCCTACAGGAAATCATCAGCCGGAGCGGGG
GGAGCGGGGCACGTTCTTCTGGGTGCGGGGGAGTCACCCCCTTCCCATAAAATGCCCCTCACCACCCTCGGCAAGCCAGGGGCTCTGCAACTTGGTTTCACACTCTATCAACGCCT
GCTAGCATCCACATTCAATCTGTCCTCGATCGCAGCCCAAAACCTCTTTCTACACCTGGCACTGTTTCCCCTCATCTGCACAGTCCTCTGGTTCTTCTACCGGGACATCAAGCACCA
GGACGGACCTTTCACGTTCCAATCACTCAACGGATTCCTGCTCAACTGCCTCATCACTTCCAGTGCATGCGCAGTTGTCAAAACTGCCTGCACATTCCCCATACACCGGACCCGAT
TTTACCAGGAGGCTCACGAAGGACTATATTCTGGCCCATTATTTTTGCTTAGTTTTAACCTATATTCCTTACCGTTTTCGATATTAACTGTAGCTATCGGGTCAAGGATATTGTTTGA
AGCTACAGGTCTAACTTCTAGTGTAGACTGGTTCTTATTTGCGGCGATACTCTGCTCCTGTTATCTACTAGCGGAGCAACAAACGATCGCTCTATTAATGGTGATTAAAGGATCATT
CATCGCCGCGATAACCAGCTTATACCTGGGCACAATATTCATCATCCTCAGTAGTGGAGCTTTGAGATCGTATGCTAGTCTACCAGAATGGTTGCTCTACCTAACCTACGCGTCAC
AAACGCGCTACTCCAGCGCGTTTTTGTCGCGTCAGCTTTTCGGGTCCGTTTACACCGCGTTGCCCGCTAACTGTACAGCGCGATTTCCCGTCAACGACGCGTTTCTATGTCGTTACA
AGGACAGTACTGCGTACCTGGCTGAACGCTTCGGTCGAGGCAGTTCAGTTTTCAACATAAATGACATGTTAGATTCCGATTTCAATCTCAGTTTATCGTATGCTTTTCCAGTCGGTT
TTGTTTTGTTGAACTGTATCCTGTATCTGATACCTTTACCCTCGTTTATAAAAGCTAAGTTTAGGGATTGA

NlABCG15

ATGGCTTTTCTGGTAGCGCGCGGCAGCGACCTTGGAGGAGGAGCAACAGATGGACATGAGCAGCAGTCGCCGGTGGCTGCACACATCAGATCACAGCTAGCATCTGACGCGGTC
ACTGCCTCCAGCAACGCCAACGCCAACACAATCATGAAGCCTGATGTACTCCATGATGCCCCCCACGATGACTGGAAGGTCCACGAGCCCTTGGCGCCCCTATGGGTGGAATCG

CATTTACCATGTCTTCAGCACCATCACTTGCCTCACAGACCACTGGAGCTTGTCTTCACCAACGTATCCTACGTTGTGGACAAAAAAGCTATTCTGAAGGACATCAGTGGTGTTGTC

AAACCAGGAGAGCTGTTGGCTGTCATGGGCCCTTCAGGCTGCGGCAAAACGACCCTTCTAAATTGCCTGGCCGGCCGATTAAAGTTAGATTCCGGAAATATTCGTCTGAACAAAG

AACGCCTTAACAAGCGCTGGAAAAGGCGGATATGCTACGTTTTGCAGCAAGATATTTTCTTCCCTGATCTCACACTTCGTCAGACCCTAGAGTATGCAGCGATGTTACGGTTACCT

GATTCACTATCTCACGCCCAAAAAATGCAATACGTTGATCACATAATTGATGTTTTGGATCTTACGAATTGCCAAGAAACAATTATAGGAGATTACATAAAAAGAGGACTGTCCG

GGGGAGAGAAGAAGCGAGCCAACATTGCCTGTGAACTACTAACCAATCCCTCATTGATGCTCCTCGATGAGCCAACTTCAGGACTGGATTCACATTCTGCTTACAATTTAATGTTG

TCATTGAAAAAGTATGCGGAAAAAGAAGGAAAAACAGTGGTTGTCACTGTTCATCAACCCTCTTCTCAAATATTCCACATGTTCGACAGACTATTATTACTTTGCAATGGAGAGA

CTGCCTATTTTGGGGACGTAAATAAAGTTGTTGACTTTTTCAACAACGTTGGACTGACGATGATGCCTCATTACAACCCTGCTGACTTTATTTTGGAGCAAGTAAAAGGAAGTGAA

GAAATGAAAGAGAAAATCATCACAGCGGCGAGAGAAGCCAGATTTCGACCCAACTATCCCCAAGAACTGATGCCGGAATATTTCAACCAATCAATGTATCTAAATAACTATCAC

GAAAGTCATTTACATTCGAATGGTCACATAGGAGGAGTACGTTGTCAATGCCAGCGAGAATTATGGAACAATTCAAGGCACTCGACATCACAGAATCTACCCTCTTCCGAAATGT

GTGTCCCGGTCGCCGTTGCGATAGGCGAAACAACCGAATCGCAGGGCCATGTTTACACAACTATTGCAGTGAAAGAAGAAGAGGGTAAAACCCTGTGGCAGGATACAGCCAGTC

ACGCGTCTTCCTCGGTTAGCAGCTCTGACGACGATGTGTCGTGGCAGTGGCCCACTTGTTTCTGGACTCAATTCAAAGTACTGAGCAGAAGAAATTTCCAAGAAGCAAGACCCAG

AATGCTTTCAACTTTGAACTGGGTGCAAACGGTAGCTTTAGGAGTGATGGCTGGACTTCTCTGGTTTCAACTGGAGAGGAAAGAGGAGTCACTACATGACATCCAGGGATGGATG

TTCTTCTCAACGACGTATTGGATGTTGTTCGCTCATTTTGGAGCTCTATCATCGTTTCCACCTGAAAGAGAAGTGATCAACAAAGAACGTCTGTCTGGAGCATATCGACTGTCGGCC

TATTACCTGGCAAAAATGGTGGGCGAATTGCCGTTGACCATCACGCTGCCAGCAGTCTATCACATCATATCCTACCCAATGCTTGGCTTCCACAGTCCTACCGTGTTTGCTACACTG

CTGGGATTCTTGCTTCTCAACACTATTGTAGCTCAGAGTGTTGGATTCTTTGTCGGCGCATGCTGCATGGACATGCAGGTGTCGATAACGATCAGTGCCCTCTACACGCTGGCCACG

CAACTGTTTGGCGGTTACCTGGCCACCAACATCCCGCCCTGGCTCAAGTGGATGCAGTATTTGTCCATGGTCCATTACGCCTATCAGAATATGCAGATTGTCGAGTTCAGCGAAGG

AGAGTGGATCAAATGCGCGCCGCAATCGAAGTTCGACGTGTGCCTCAACAACTCGACGACGCACATCCCCGTGGCGTCAATCCTGGAGGTGCAGGGTGCCAGCCTGCCGCTCTG

GGCCAACACCCTCGTGCTGCTCCTCTTCCTGCTCATATTCCGCGTGCTTGGCTACATCGTGCTCAGATACTTCCGTCGACCCAAGTGA

NlABCH1

ATGTTCGACAATAACAACGTGATGAGCTACTCTCAGGGGGACATGGAGCTGAATTGTCCCCAGCCCATGGTGGCTGTGAATGGAGCATTCAAGAGATACACCTCCAAAAGTCCA

ATCTTGCTGCGAGGATTTAGTATGACTGTACCTACTGGAGCTATATACGGACTGCTGGGACCGAGTGGCTGTGGCAAAACTACTCTACTCAATTGTATTGTTGGCAACAGGCGGCT

GGATAGCGGGCAAGTCACGTTGGGCATCACCAAACGCAAGGAGCTCGGATACATGCCACAGGAGGTAGCACTGAACGAGGAATTCACGATAGAGGAGACTTTCTTCTTCTATGG

GAAACTTTTCGACATGAGCAGAGCGCAAGTGAAAATGCGCATGAAGGAATTGTTGACATTTTTCGATCTGCCAACAGAGGGCTTCATTGGTGAGTTAAGTGGTGGTCAACAGAGA

AGAGTTTCATTCGCAGTAGCTTTATTACACAATCCTCAACTCCTCATCTTGGATGAACCAACGGTCGGTGTTGATCCTGTCCTGTGTAGTAGGATATGGGATGCGCTTGTTAAAATG

GCTTTAGAAGAAAAGAAAACTATTATTATCACAACACATTACATTGAGGAGGCTCGGCAATGCCAACTAATTGGATTGATGAGAGGCGGAGTTCTCCTTGCTGAAGAGCCTCCCT

TGACTCTGATGGCATCTCATAATTGTAACACACTGGAGCAAGTGTTCCTCGAGCTAAGTCAAAAACAGCACGAAGCGTCAAACAAATCAAACGAAGATTTAACAGAGAGTGATA

CTGATTATAAAAAGATGGCAAATCAACATTTTCAACCTCCTCTAATATCTAGTCAAATGTGGAAGAAATCCAGATTCTCAGCTCAGTTATTTAAAAATGTCGTATGGATGAAACGA

AATGTGCCAATAATGATGTTCCTGCTGTTCCTGCCATTCCTGCTATTCACAATCTACTGTTCAGTGTTCGGCAAGACACCGACTGGCTTGTCGCTGGGAATTGTTAGCGAGGAATTG

GGCAGTTTGGCCGACTGTCAGGCGAACTCCACAGAGCAGGCCTTCAACTACACCGACTTCAACTGCTATTTCGACAAACCACTGAGTTGTCTCTACTTCGAGCACCTCAAGGAAA

ACGATTTCAATCTGATAGAGTACAACAAGCTTGAAACTGGGCAAACAGCTCTGGAGAGGAATAAGGTATGGGGTCTCCTGCATTTCACCAGGAACTACACGATAGCCGTCTTGG

AGAGAATTGATTACGGCTTCGACACCGCTGAATCAGTTGTCAGAGATGGCACTGTCGATGTTAGACTCGATATGTCAGATCATGTCATTGGAACCAGCCTGCAAAGGATGCTGCT

GACCACCTATAACAACTTCATTGGAGAAGTTTATGAAGCATGTGGAAGTATTGCCGAGGCAGGAAAGATTCCAATCAAGTATAATGAACCAATCTACGGTGGAAGTGACCCTCTT

TTTTCATACACCACGTTACCTGGATACTTGATGTCATTCAGCTTTTATCTGCCTATGCTCTTTACATCAGGCGCTATCATGCTGGAGAAACTGAGTGGTCTTATGGAGAGGAAAATG

ATTGCAGGAATGACAATGCTGGAAATAGTTACCGCTCATGCAGTTGTACAGATCTTCATCCTCTTCTGTCAAACTGGAATCATTTTCTACACAGCTTATGGCATTTTCGATAATCCA

GTTGAAGGGGACCTCTACCTCCTGATTCTCCTCACATTCATCAATGAAATTGCTGGGATGTGCTATGGTTTCATGCTCAGTGAAATTCATGACTCAGATACAAATTGCTCTTATGCT

GGAACGGGAACAGTATTAGCACTGTTCATGACTACAGGCACTCTGTGGCCAATGGAAGGCGCGCACCCAATCATGCGTGCAGCCATGTGGGTTCTTCCAGTGAACGCGGCAGTC

ACCAGCTACCACAGCATAGCACTGCGAGGCTACAGCATCTCAAACCCAGTCGTCTACAGGGGATTCCTGTCAACACTGGCCTGGTGCGCCTTCTTTGCTATCGGTGCCATCCTCAT

CAACAGAGTCAGAAAAGGCGTCAGATAG
